# Development of a rapid and accurate CRISPR/Cas13-based diagnostic test for GII.4 norovirus infection

**DOI:** 10.3389/fmicb.2022.912315

**Published:** 2022-08-24

**Authors:** Lei Duan, Xiaohan Yang, Wenli Zhan, Yuan Tang, Mengru Wei, Keyi Chen, Pan Liu, Jia Xie, Changbin Zhang, Hongyu Zhao, Mingyong Luo

**Affiliations:** Medical Genetic Centre, Guangdong Women and Children Hospital, Guangzhou, China

**Keywords:** CRISPR/Cas13a, assay, gastroenteritis, norovirus, GII.4 genotype

## Abstract

Genogroup II genotype 4 (GII.4) norovirus causes acute gastroenteritis in children, and its infection is more severe than that of other genotypes. Early and precise detection and treatment are critical for controlling its spread and reducing the severity of infection. In this study, a rapid and efficient isothermal assay for the GII.4 norovirus detection (GII.4-CRISPR detection) was developed based on the CRISPR/Cas13a system. The assay can be applied without expensive instrumentation, and the results can be read *via* both fluorescence and lateral flow strip (LFS). The analytical sensitivity of this assay was 5 copies/reaction, and there was no cross-reaction with other genotypes of norovirus or other clinically common pathogens. There was a coincidence rate of 100% between our assay and commercial quantitative polymerase chain reaction. GII.4-CRISPR detection improves upon the shortcomings of some previously established molecular methods of detection, particularly with regard to accessibility. It provides an alternative tool for outbreak control and early diagnosis of GII.4 norovirus infection.

## Introduction

Norovirus is one of the predominant causes of acute gastroenteritis worldwide, with approximately 699 million people infected annually (Ahmed et al., 2014; Van Trang et al., 2014; Hamzavi et al., 2018). Norovirus belongs to the family *Caliciviridae* and is a single-stranded positive-sense RNA virus with a gene length of approximately 7.5 kb. The norovirus VP1 gene can be divided into 10 genogroups (GI–GX) and at least 49 genotypes (Chhabra et al., 2019). More than 90% of norovirus infections are caused by genogroup II (GII) norovirus, and GII genotype 4 (GII.4) norovirus has been the predominant strain over the last two decades, with a new GII.4 strain appearing every 2–4 years (Bull and White, 2011; van Beek et al., 2018; Cannon et al., 2021).

Acute gastroenteritis caused by this virus is normally self-limiting, though the virus is highly infectious, with only a small viral load enabling transmission. Norovirus outbreaks usually appear in semi-closed settings, such as kindergartens, schools, medical facilities, and military camps (Robilotti et al., 2015; Parra, 2019). Moreover, infection can cause serious symptoms in groups with low immunity, including children, the elderly, and immunocompromised individuals (Robilotti et al., 2015). The majority of severe infections are caused by GII.4 norovirus, which is associated with a higher rate of hospitalization and mortality than the other genotypes (Desai et al., 2012). In addition, pediatric patients are more commonly infected with GII.4 norovirus and tend to have more severe diarrhea and vomiting (Duan et al., 2021; Haddadin et al., 2021). Therefore, in the absence of specific drugs and vaccines, rapid and accurate detection is critical to control the spread of GII.4 norovirus and to reduce social and economic impacts.

Currently, norovirus is primarily detected using molecular analyses. Quantitative real-time polymerase chain reaction (qPCR) has been widely used in clinical laboratories and is regarded as the gold standard for norovirus detection. qPCR has higher sensitivity and specificity than electron microscopy and immunoassays (Vinje, 2015). However, it requires expensive and sophisticated thermal cyclers to complete the assay, which is not feasible for smaller, more basic laboratories. As alternatives, methods involving isothermal amplification technology have also been applied for the detection of norovirus, such as nuclear acid sequence-based amplification (Patterson et al., 2006), loop-mediated isothermal amplification (Luo et al., 2014), and recombinase polymerase amplification (RPA; Han et al., 2020; Jia et al., 2020). Unlike qPCR, isothermal amplification techniques do not require the use of sophisticated thermal cyclers, as in qPCR, which allows for simplification of the procedure and improved accessibility.

**Figure 1:**
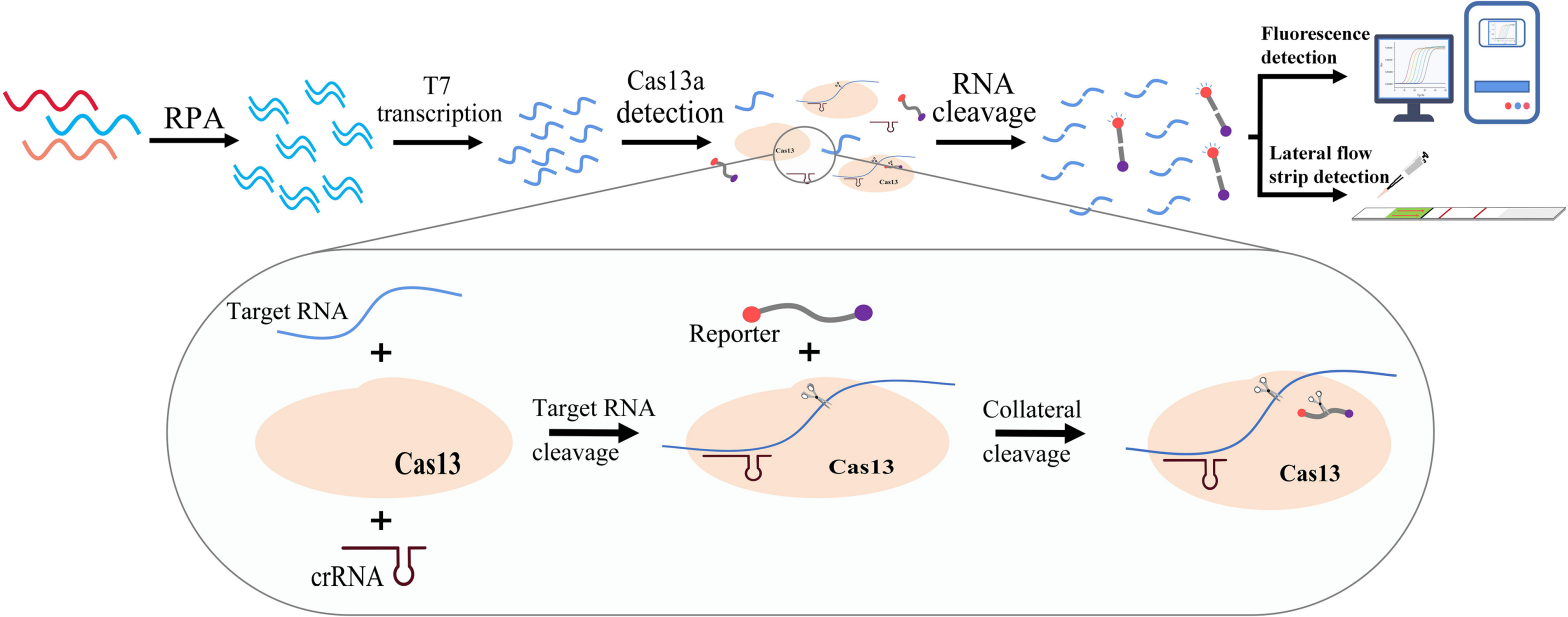
Schematic of the principle of the GII.4-CRISPR detection. The sample is amplified by RPA, and then a large amount of target RNA is produced by T7 transcriptase. The CRISPR/Cas13 system can recognize and cleave the target RNA, and simultaneously activate its collateral-cleavage activity to cleave the RNA reporter. The detection result can be confirmed by monitoring the signal of the RNA reporter.

The clustered regularly interspaced short palindromic repeats (CRISPR)-associated protein (Cas) system is an acquired immune system in some bacteria and archaea that can protect against viral and plasmid invasion by recognizing specific nucleic acid sequences (Deveau et al., 2010). Its ability to identify and cleave target nucleic acids rapidly, efficiently, and specifically is perfectly suited for genetic engineering. In recent years, the CRISPR/Cas system has contributed to rapid progress in the field of molecular detection, and assays based on Cas9, Cas12, Cas13, and Cas14 have been developed (Zhu et al., 2020). One of these assays is known as the Sherlock detection platform, developed based on the CRISPR/Cas13 system, which can detect target RNA with excellent accuracy (Abudayyeh et al., 2016). It has been developed for a variety of pathogens (Gootenberg et al., 2018; Joung et al., 2020), such as Zika virus, dengue virus, and COVID-19. In this study, given the widespread prevalence and severe symptoms of GII.4 norovirus infection, we developed a rapid and efficient isothermal assay for the diagnosis of GII.4 norovirus infection based on the CRISPR/Cas13a system (GII.4-CRISPR detection) by modification of the Sherlock detection platform (Kellner et al., 2019). This assay combines RPA T7 transcription and the CRISPR/Cas13a system, which works at low temperatures and can quickly detect GII.4 norovirus (Figure 1). Our work provides a simple and effective novel method for the early detection of GII.4 norovirus infection, which may in turn facilitate improved treatment outcomes.

## Materials and methods

### Clinical sample collection and RNA extraction

In this study, 7 Norovirus samples of GII.4 and other genotypes, including stool swabs and vomit swabs, were collected from patients under 5 years of age who attended the Guangdong Women and Children Hospital in Guangdong Province, China, between 2020 and 2021. In addition, 5 clinical samples from patients with rotavirus (RV), enterovirus (EV), adenovirus (AdV), respiratory syncytial virus (RSV), and Epstein–Barr virus (EBV) infections were also collected for the evaluation of this assay. This study was approved by the Ethics Board of the Guangdong Women and Children Hospital. All patient information was anonymized before the analysis, and informed consent was obtained. All samples were stored at −80°C until further analyses.

Samples positive for norovirus were confirmed using the Diagnostic Kit for Norovirus RNA (Land Medical, Wuhan, China) on the ABI 7500 Fast Real-Time PCR Platform (Applied Biosystems, Foster City, CA, United States). The norovirus genotype was then determined by sequencing a fragment located in the ORF1-ORF2 junction as has been described previously (Cannon et al., 2017).

Before the assay, RNA was extracted from clinical samples using the QIAamp Mini Virus RNA Kit (Qiagen, Hilden, Germany) as per the manufacturer’s protocol. The complementary DNA (cDNA) of RNA virus samples was synthesized using the PrimerScript™ 1st Strand cDNA Synthesis Kit (TaKaRa, Dalian, China) before detection.

### Primer and CRISPR RNA (crRNA) design

A local database was constructed based on the norovirus VP1 genes obtained from GenBank to identify specific primers and crRNA (Supplementary Table 1). After multiple alignments using MEGA X, the conserved sequence was identified in the VP1 gene near the ORF1-ORF2 junction. Primers were designed for this region according to the TwistAmp® Basic Kit (TwistDx, Cambridge, England). In order to transcribe the RPA product into target RNA, which was then recognized by LwaCas13a, a T7 promoter sequence (GAAATTAATACGACTCACTATAGGG) was added to the 5′ end of the forward primer. The spacer region of crRNA requires a strict match with the target RNA; therefore, the most conserved sequence in the target RNA was selected as the target site of Cas13/crRNA. The primers, crRNA, and reporter for this assay are listed in Table 1. The primers were synthesized by Sangon Biotech (Shanghai, China), and the crRNA and reporter were synthesized by Bio-LifeSci (Guangzhou, China).

**Table 1:** Primer and probe sequences used for GII.4-CRISPR detection.

Name	Sequence (5′−3′)
Forward primer	TAATACGACTCACTATAGGGCTGGCTCCCAATTTTGTGAATGAAGATGGCG
Reverse primer	CTCCAAAGCCATAACCTCATTGTTGACCTCTG
crRNA	GAUUUAGACUACCCCAAAAACGAAGGGGACUAAAACACGAGGUUGGCUGCGGACCCAUCAGAUG
Flu-RNA reporter	FAM-rUrUrUrUrUrU-BHQ1
LF-RNA reporter	FAM-rUrUrUrUrUrU-Biotin

FAM, Carboxyfluorescein; dSpacer BHQ, Black Hole Quencher.

### GII.4 norovirus standard plasmid construction

In order to optimize reaction conditions and verify sensitivity, we constructed a standard plasmid containing the target sequence of this assay, which was obtained from clinical samples infected with GII.4 norovirus (Supplementary Figure 1). A 572 bp fragment located in the ORF1-ORF2 junction of GII.4 norovirus was amplified by PCR (Cannon et al., 2017), and the product was purified and cloned into the pESI-T simple vector to construct a recombinant plasmid. The plasmid was quantified using a NanoDrop 2000 spectrophotometer (Thermo Fisher Scientific, Waltham, MA, United States) for subsequent steps.

### GII.4-CRISPR detection

GII.4-CRISPR detection combines RPA amplification, T7 transcription, and the CRISPR/Cas13a system into a single reaction, and the results can be read *via* fluorescence (GII.4-CRISPR-Flu detection) and lateral flow strip (LFS; GII.4-CRISPR-LFS detection). A reaction volume of 50 μl was used for GII.4-CRISPR-Flu detection, containing 29.5 μl RPA rehydration buffer (TwistAmp® Basic kit, TwistDX, United Kingdom), 2.4 μl forward primer (10 μm), 2.4 μl reverse primer (10 μm), 0.25 μl LwaCas13a (10 μm; Bio-LifeSci), 0.125 μl crRNA (10 μm; Bio-LifeSci), 1.5 μl T7 RNA polymerase (50 U/μl; Takara, Beijing, China), 2 μl ribonucleoside triphosphate (rNTP) mix (50 mm), 2 μl RNase inhibitor (40 U/μl; Takara, Beijing, China), 0.5 μl MgCl_2_ (500 mm), 1 μl fluorescent RNA reporter (10 μm), and 0.825 μl RNase-free ddH_2_O. The reaction mixtures were vortexed and spun briefly, followed by addition of 5 μl of template and 2.5 μl of magnesium acetate (280 mm) to the tube cap. This mixture was again vortexed and centrifuged, before being placed in an ABI7500 Fast Real-Time PCR platform at 37°C. The fluorescence intensity was measured every minute.

Only few adjustments were made to the GII.4-CRIPSR-Flu detection protocol for GII.4-CRIPSR-LFS detection. Volumes of LwaCas13a and cRNA were increased to 0.5 and 0.25 μl, respectively. Additionally, 1 μl of fluorescent RNA reporter (10 μm) was changed to 1 μl of LFS-RNA reporter (10 μm), and the reaction mixture was incubated at 37°C. On completion of the reaction, 20 μl of product was pipetted into a 100 μl Hybridetect Assay Buffer (Milenia Biotec GmbH, Germany) and mixed thoroughly, and the LFS was inserted into the solution.

### Evaluation of the sensitivity and specificity of GII.4-CRISPR detection

In evaluating the sensitivity of GII.4-CRISPR detection, ten-fold serial dilutions ranging from 10^5^ to 10^0^ copies/μL of the GII.4 norovirus standard plasmid were used as templates to determine the limit of detection (LOD). In addition, the specificity was evaluated using clinical samples from patients with norovirus (GII.4, GII.2, GII.3, GII.6, GII.13, and GII.17) and other pathogens that cause symptoms similar to norovirus or are frequently detected in clinical practice (RV, EV, AdV, RSV, and EBV). All assays were replicated thrice, and statistical analyses were performed using GraphPad Prism 8 software (GraphPad Software, La Jolla, CA, United States).

### Validation using patient samples

To validate the clinical performance of GII.4-CRISPR detection, 65 clinical samples of patients with suspected norovirus infection were selected randomly from patients attending Guangdong Maternal and Child Health Hospital between 2020 and 2021. Furthermore, qPCR was performed in parallel with GII.4-CRISPR detection and the positive samples were genotyped by sequencing as has been described (Duan et al., 2021).

## Results

### Time optimization of GII.4-CRISPR detection

**Figure 2:**
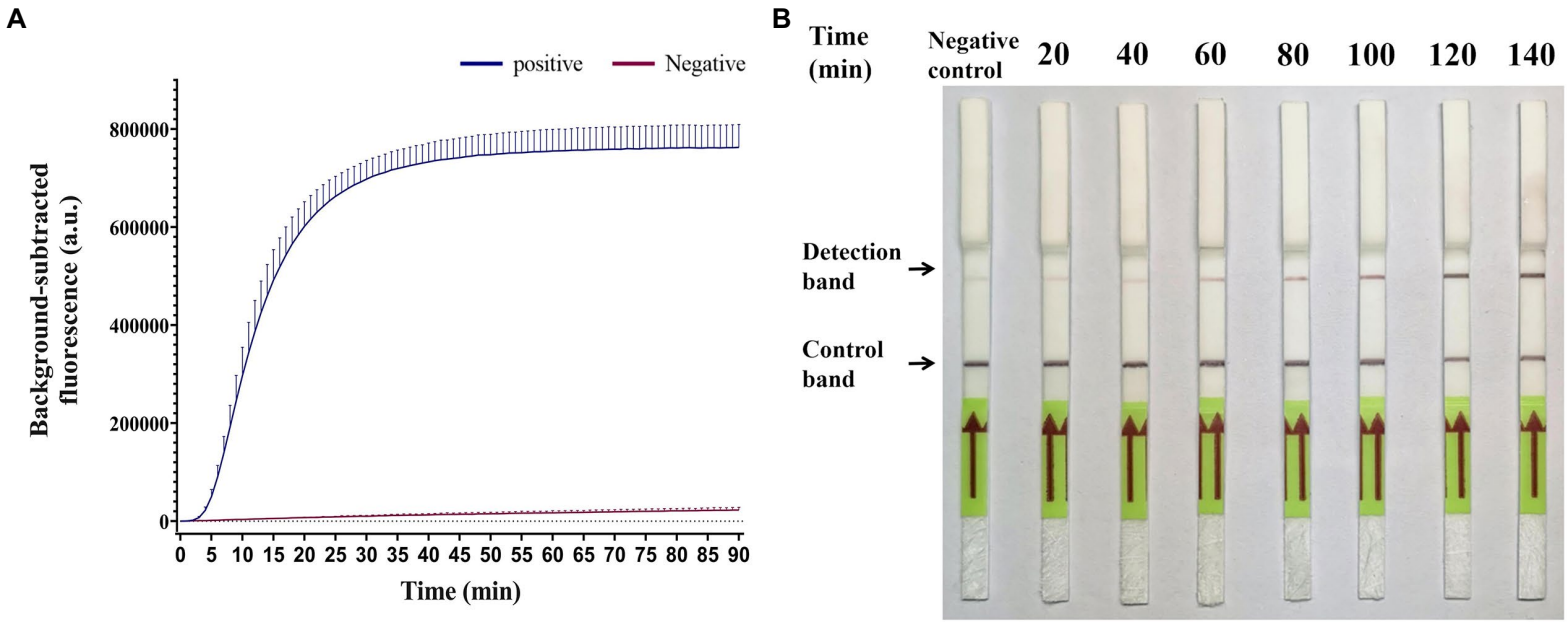
Time optimization of the GII.4-CRISPR detection. **(A)** The background-subtracted fluorescence intensity of GII.4-CRISPR-Flu detection varies with time. **(B)** The color intensity of the GII.4-CRISPR-LFS detection changes in different reaction times.

To determine the optimal time for GII.4-CRISPR detection, a GII.4 norovirus standard plasmid at a concentration of 10^5^ copies/μl was used as the positive control, and an equivalent volume of water was used as the negative control. As shown in Figure 2A, the effective fluorescence signal began to be monitored at approximately 5 min, and at approximately 40 min, the reaction reached a plateau. Thus, GII.4-CRISPR-Flu detection was completed after 40 min. As shown in Figure 2B, the color intensity of the detection band gradually increased with time in the first 2 h; however, no significant increase was observed after 2 h. Hence, the completion time of GII.4-CRISPR-LFS detection was 2 h.

### Sensitivity of GII.4-CRISPR detection

**Figure 3:**
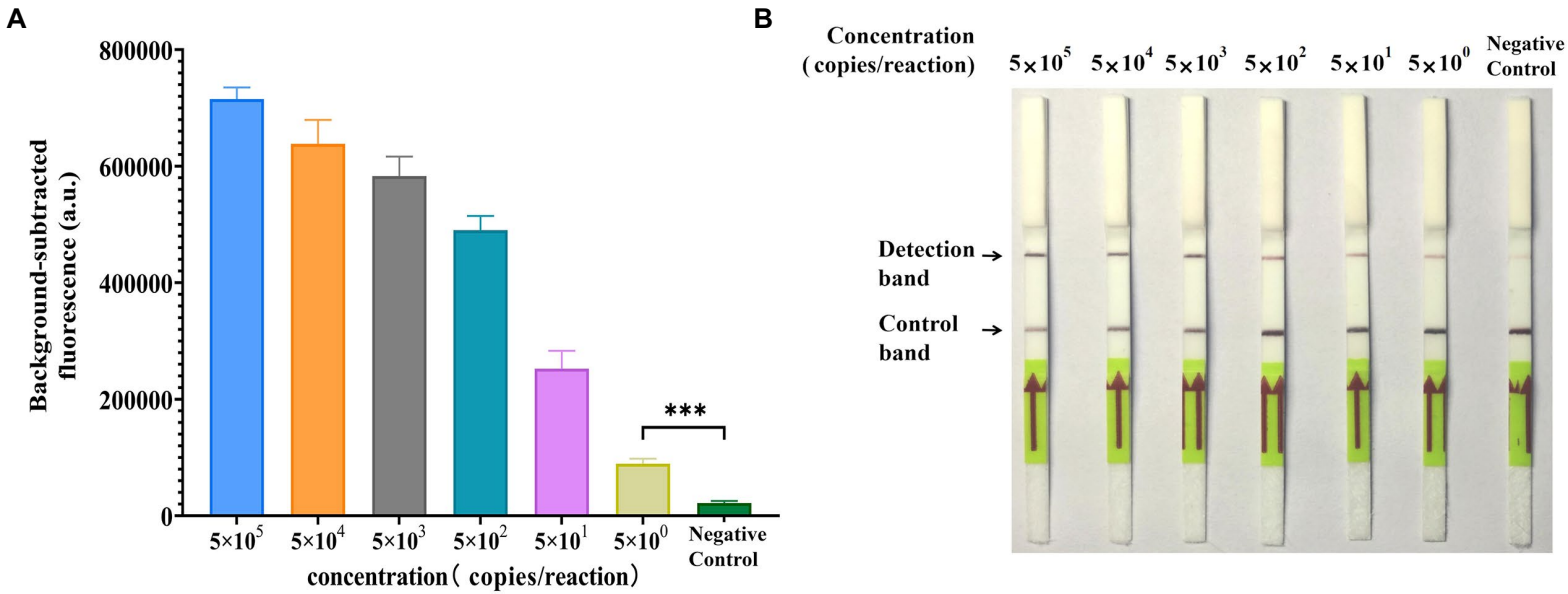
Sensitivity of the GII.4-CRISPR detection. **(A)** The histogram of background-subtracted fluorescence intensity at the end of the detection (40 min). Values of the graphs represent the mean ± SD (*n* = 3); ***p < 0.001. **(B)** The result of GII.4-CRISPR-LFS detection after 2 h reaction for the sensitivity determination.

To verify the sensitivity of GII.4-CRISPR detection, ten-fold serially diluted GII.4 norovirus standard plasmid at a concentration range of 10^5^ to 10^0^ copies/μl was used to determine the LOD. As shown in Figure 3A, a significant fluorescence signal was observed when the concentration exceeded 5 × 10^0^ copies/reaction. In Figure 3B, the detection band was observed on the LFS when the concentration exceeded 5 × 10^0^ copies/reaction during GII.4-CRISPR-LFS detection. These results indicate that the LOD of GII.4-CRISPR detection can reach 5 × 10^0^ copies/reaction for both fluorescence and LFS.

### Specificity of GII.4-CRISPR detection

**Figure 4:**
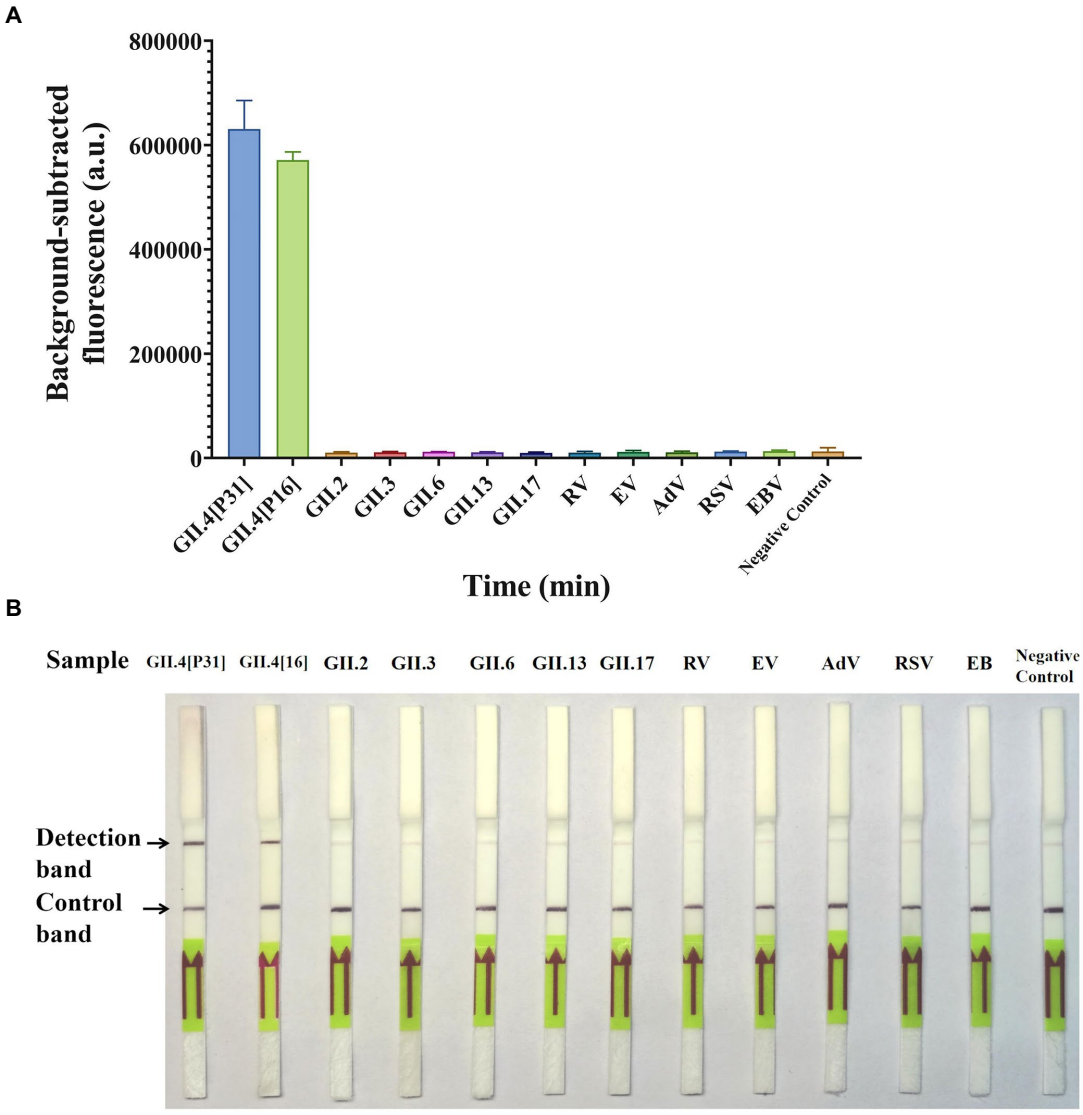
Specificity of GII.4-CRISPR detection for 13 virus species. **(A)** Background-subtracted fluorescence intensity of GII.4-CRISPR-Flu detection, values represent the mean ± SD (*n* = 3). **(B)** The result of GII.4-CRISPR-LFS detection after 2 h reaction for the sensitivity determination. GII.4[P31] and GII.4[P16] are two variants of GII.4 norovirus with differences in the RdRp gene, which belong to [P31] and [P16], respectively.

The specificity of GII.4-CRISPR detection was evaluated using different genotypes (GII.4[P31], GII.4[P16], GII.2, GII.3, GII.6, GII.13, and GII.17) of norovirus and other clinically common virus species (RV, EV, AdV, RSV, EB, and CMV). Figure 4A shows that the fluorescence signal of GII.4-CRISPR-Flu detection was only observed in the GII.4 norovirus samples (both GII.4[P31] and GII.4[P16] strains), but not in the other species. Similarly, as shown in Figure 4B, consistent results were observed using GII.4-CRISPR-LFS detection. Thus, GII.4-CRISPR detection can specifically detect GII.4 norovirus without cross-reactivity with other virus species.

### Validation using clinical samples

To validate the clinical performance of the GII.4-CRISPR detection, 65 samples from patients with suspected norovirus infections were randomly selected. All samples were tested using qPCR and GII.4-CRISPR detection. qPCR analysis showed that 24 samples were positive for GII.4 norovirus, 4 samples were positive for noroviruses of other genotypes (3 of GII.2 and 1 of GII.17), and 37 samples were negative for norovirus. Meanwhile, all 24 GII.4 norovirus-positive samples detected by qPCR were also detected by GII.4-CRISPR detection (GII.4-CRISPR-Flu detection and GII.4-CRISPR-LFS detection), while all other samples tested negative (Table 2; Supplementary Figure 2). These results indicate that GII.4-CRISPR detection has comparable performance with that of qPCR, and it can be performed at a lower temperature and in less time.

**Table 2:** Comparison of clinical performance between GII.4-CRISPR detection and qPCR.

		qPCR			Total
		GII.4 positive	Non-GII.4 positive	Negative	
GII.4-CRISPR-Flu detection	Positive	24	0	0	65
	Negative	0	4^*^	37	
GII.4-CRISPR-LFS detection	Positive	24	0	0	
	Negative	0	4	37	

* The 4 non-GII.4 positive samples included 3 samples of GII.2 and a sample of GII.17.

## Discussion

GII.4 norovirus is the predominant strain of norovirus worldwide. Infection with this strain causes more severe symptoms than with other strains, which leads to higher rates of hospitalization and mortality, especially among children (Desai et al., 2012; Cannon et al., 2021; Duan et al., 2021; Haddadin et al., 2021). Developing rapid and efficient methods for the detection of GII.4 norovirus is important to prevent its spread and allow early intervention to alleviate severe illness (Liu and Moore, 2020; Wang et al., 2021). PCR testing for norovirus has been widely used in clinical diagnosis and is considered the gold standard for norovirus detection (Vinje, 2015), but it is time-consuming and requires sophisticated equipment that hampers its further utilization. In this study, we developed a novel nucleic acid isothermal assay based on the RPA and CRISPR/Cas13a systems, which has high sensitivity and specificity for the detection of GII.4 norovirus. The GII.4-CRISPR detection had an LOD up to 5 copies/reaction, and it could accurately and specifically detect GII.4 norovirus, with no cross-reaction with other norovirus genotypes and other clinically common virus species.

In recent years, several groups have developed detection methods for GII norovirus based exclusively on RPA, and all have shown relatively high sensitivity (Moore and Jaykus, 2017; Ma et al., 2018; Han et al., 2020; Jia et al., 2020). Moore et al. developed an RPA assay for GII.4 norovirus that can identify two GII.4 norovirus strains that have slight differences in target sequences (Moore and Jaykus, 2017). Their assay, however, identified GII.3 norovirus inconsistently, therefore compromising specificity. This may have been caused by mismatches in RPA amplification. Boyle et al. found that RPA primers and probes could tolerate up to nine base mismatches with the target sequence (Boyle et al., 2013), which reduces the specificity of the target sequence. Therefore, we introduced the CRISPR/Cas13a system to overcome this limitation. In the CRISPR/Cas13a system, only when the crRNA is precisely complementary to the target RNA can Cas13a be activated to cleave the RNA probe. Abudayyeh et al. showed that the CRISPR/Cas13a system can only tolerate up to one mismatch between the crRNA and the target (Abudayyeh et al., 2016). Furthermore, Gootenberg introduced synthetic mismatches in the crRNA/target duplex for virus detection, which could identify specific strains of the Zika and dengue viruses (Gootenberg et al., 2017). This strict complementarity is sufficient to enable a highly specific detection of GII.4 norovirus by GII.4-CRISPR detection. In this study, a norovirus VP1 gene database downloaded from GenBank was constructed to identify the specific primers and crRNA. Our results showed that the primers and crRNA designed in this assay had specific detection performance for GII.4 norovirus and had no cross-reaction with other viruses.

Qian et al. have also described a CRISPR-based method for detecting GII.4 norovirus (Qian et al., 2021). The assay they developed was based on the CRISPR/Cas12 system and involved dividing RPA amplification and CRISPR detection into separate tubes, which complicated the assay process and increased the chances of nucleic acid contamination. In this study, we used the CRISPR/Cas13a system and combined the two-step reaction into a single tube, which helped to simplify the procedure and minimize the chances of nucleic acid contamination, and this is consequently more promising for general application. Moreover, the assay was performed at a lower temperature, and thus did not require sophisticated thermal cyclers. Furthermore, the results of this assay can be read using fluorescent signals (GII.4-CRISPR-Flu detection) as well as LFS (GII.4-CRISPR-LFS detection). This enables our method to be applied in both well-equipped clinical laboratories for fast fluorescence detection and in some low-resource settings, such as basic laboratory settings or rural health clinics, for visual detection using the equipment-independent LFS method. In particular, GII.4-CRISPR-LFS detection is ideally suitable for in-field and point-of-care testing, which provide timely detection and early diagnosis of GII.4 norovirus infection. This is expected to greatly enhance effective control of the spread of outbreaks.

In conclusion, we have established a rapid, highly sensitive, and specific GII.4 norovirus assay based on CRISPR/Cas13a. It can be applied without expensive instrumentation, and the results can be read *via* both fluorescence and LFS. This novel method provides a new strategy for outbreak control and the early diagnosis of GII.4 norovirus infection.

## Data availability statement

The original contributions presented in the study are included in the article/Supplementary material, further inquiries can be directed to the corresponding author.

## Author contributions

LD, XY, and ML designed the experiments and wrote the manuscript. WZ, JX, YT, and MW performed the experiments. PL, CZ, and HZ analyzed the data. All authors contributed to the article and approved the submitted version.

## Funding

This work was supported by the Guangzhou Science, Technology and Innovation Commission (grant number 201904010452). The funders had no role in study design, data collection, data analysis, data interpretation, writing of the report, or decision to submit for publication.

## Conflict of interest

The authors declare that the research was conducted in the absence of any commercial or financial relationships that could be construed as a potential conflict of interest.

## Publisher’s note

All claims expressed in this article are solely those of the authors and do not necessarily represent those of their affiliated organizations, or those of the publisher, the editors and the reviewers. Any product that may be evaluated in this article, or claim that may be made by its manufacturer, is not guaranteed or endorsed by the publisher.

## Abbreviations

GII: genogroup II
GII.4: GII genotype 4
qPCR: Quantitative real-time polymerase chain reaction
RPA: Recombinase polymerase amplification
CRISPR: Clustered regularly interspaced short palindromic repeats
Cas: CRISPR-associated protein
RV: Rotavirus
EV: Enterovirus
AdV: Adenovirus
RSV: Respiratory syncytial virus
EBV: Epstein–Barr virus
CMV: Cytomegalovirus
LFS: Lateral flow strips
LOD: Limit of detection
