# Supplementary material for: Development of a rapid and accurate CRISPR/Cas13-based diagnostic test for GII.4 norovirus infection
Source: Front Microbiol. 2022 Aug 24;13:912315. doi: 10.3389/fmicb.2022.912315 (PMC9449452; doi:10.3389/fmicb.2022.912315)
Supplement: Supplementary file 1 [file Data_Sheet_1.docx]

Supplementary Material


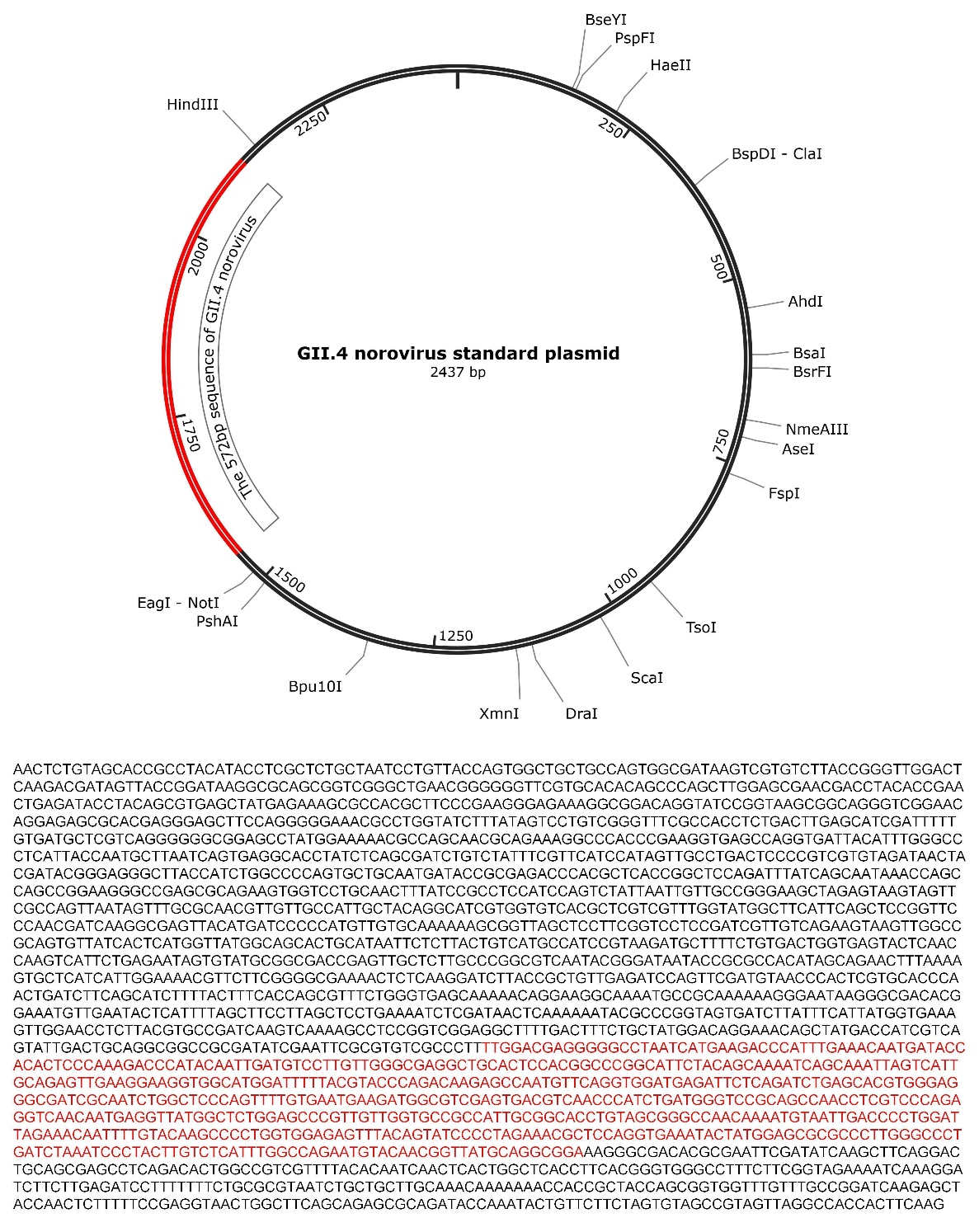


**Supplementary Figure 1** Schematic of the principle of the GII.4 norovirus standard plasmid. The red line represents the position of the 572 bp sequence fragment of GII.4 norovirus in the plasmid, and the sequence information of the recombinant plasmid is below the schematic.


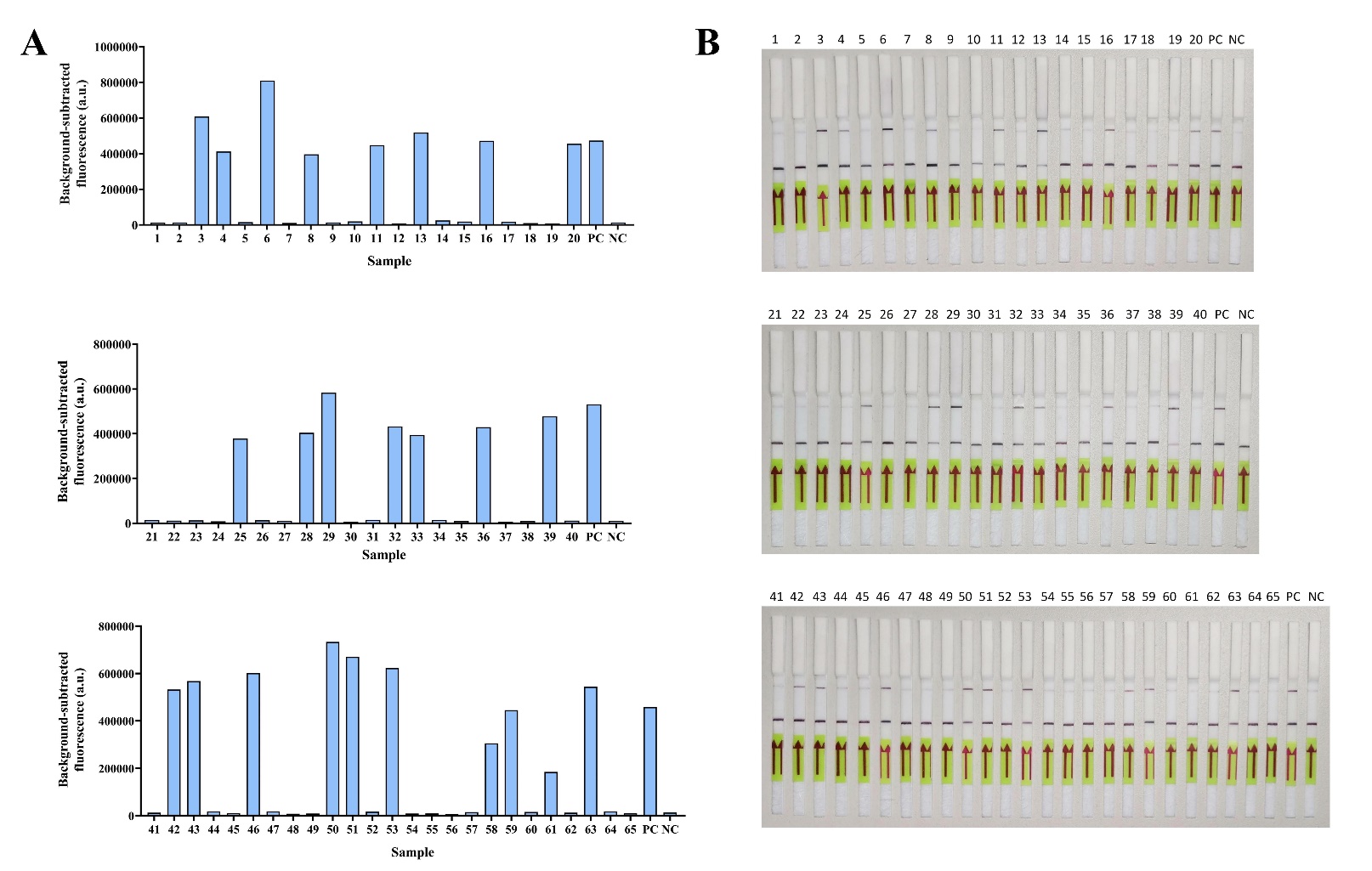


**Supplementary Figure 2** The results of the clinical performance validation experiments for GⅡ.4-CRISPR-Flu detection (A) and GⅡ.4-CRISPR-LFS detection (B). PC, positive control; NC, negative control.

**Supplementary Table 1** The sequences for constructing the local database.

| Name | Sequence |
| --- | --- |
| FJ537134.1 USA/1974/GII.4(P39) | ATGAGGACCCATCTGAAACAATGATACCACATTCCCAAAGGCCCACGCAGTTGATGTCTTTGCTGGGTGAGGCTGCACTGCACGGCCCAGCATTCTACAGCAAGATCAGTAAACTAGTCATCACAGAGTTAAAGGAAGGTGGCATGGATTTTTACGTGCCCAGACAAGAGCCGATGTTCAGGTGGATGAGATTCTCAGACCTGAGCACGTGGGAGGGCGATCGCAATCTGGCTCCCAGTTTTGTGAATGAAGATGGCGTCGAGTGACGCCAACCCATCTGATGGGTCCGCAGCCAACCTCGTCCCAGAGGTCAACAATGAGGTTATGGCTTTGGAGCCTGTTGTTGGTGCTGCCATTGCAGCACCTGTGGCAGGCCAACAAAATATAATTGACCCCTGGATTAGAAATAATTTTGTACAAGCCCCTGGTGGAGAGTTCACAGTGTCCCCTAGAAACGCTCCAGGTGAGATACTATGGAGCGCGCCCTTGGGCCCTGATTTGAACCCCTATCTTTCTCACCTGTCCAGAATGTACAATGGTTATGCAGGTGGTTTTGAAGTGCAAGTGATCCTCGCAGGGAACGCGTTCACCGCCGGGAAAGTCATATTTGCAGCAGTTCCACCAAATTTTCCAACTGAAGGTCTAAGCCCCAGCCAGGTTACTATGTTCCCCCATATAATTGTAGATGTTAGGCAATTGGAACCTGTATTGATCCCCCTACCTGATGTTAGGAATAATTTCTATCATTATAATCAAGCACATGATTCTACTCTCAAATTGATAGCAATGTTGTACACACCACTCAGAGCTAATAATGCCGGGGATGATGTCTTTACAGTCTCTTGTCGAGTCCTCACGAGACCATCCCCCGATTTTGATTTTATATTCCTGGTGCCACCCACAGTTGAATCAAGAACTAAACCATTTACTGTCCCAATCTTAACTGTTGAGGAAATGTCCAATTCAAGATTCCCCATTCCTTTGGAAAAGCTGTACACGGGTCCTAGCAGTGCTTTTGTTGTCCAACCACAAAATGGCAGATGCACGACTGATGGCGTGCTCTTAGGCACTACTCAGCTGTCAGCTGTCAATATCTGCAACTTCAGGGGGGATGTCACCCGTGTTGGGATCAGCCATGATTATACAATGAATTTGGTGTCCCAAAATTGGAATAATTATGATCCAACAGAAGAAATTCCAGCTCCCCTGGGAACACCAGACTTTGTAGGAAAGATCCAAGGTTTGCTCACCCAAACCACAAGAGCGGACGGCTCGACCCGCGCCCACAAAGCTACAGTGAGCACTGGGAGTGTCCACTTCACTCCAAAGCTGGGTAGTGTTCAATTCACCACCGATACGAACAATGATTTCCAAACTGGCCAAAACACAAAATTTACCCCAGTTGGTGTCATCCAAGACGGTGATCACCATCAGAATGAACCCCAACAATGGGTACTCCCAAATTACTCAGGCACATCTGGTCACAATGTGCATCTGGCCCCTGCCGTTGCCCCCACTTTCCCGGGTGAGCAGCTTCTTTTCTTTAGGTCCACTATGCCCGGGTGTAGCGGGTACCCCAACATGAATTTGGATTGCCTACTCCCCCAGGAGTGGGTGCTGCACTTTTACCAGGAAGCAGCCCCAGCACAATCCGATGTGGCCCTGCTGAGATTTGTGAATCCAGACACAGGTAGGGTTCTGTTTGAGTGCAAACTCCATAAGTCAGGCTATATCACAGTGGCTCATACTGGCCCGTATGATTTGGTTATCCCCCCCAATGGTTACTTCAGATTTGATTCTTGGGTCAACCAGTTCTACACACTCGCCCCCATGGGAAATGGAACGGGGCGCAGGCGTGCTTTATAATGGCTG |
| FJ537136.1 USA/1988/GII.4 Camberwell | ATGAAGACCCATCTGAAACAATGATACCACACTCCCAAAGACCCATACAACTAATGTCTTTACTGGGCGAGGCCGCACTCCACGGCCCGGCATTCTACAGCAAAATTAGCAAGCTAGTCATTGCAGAGCTGAAGGAAGGTGGCATGGATTTTTACGTGCCCAGGCAAGAACCAATGTTCAGATGGATGAGATTCTCAGATCTGAGCACGTGGGAGGGCGATCGCAATCTGGCTCCCAGTTTTGTGAATGAAGATGGCGTCGAGTGACGCCAACCCATCTGATGGGTCCGCAGCCAACCTCGTCCCAGAGGTCAACAATGAGGTTATGGCTCTGGAGCCCGTTGTTGGTGCTGCTATTGCAGCACCTGTAGCGGGCCAACAAAATATAATTGACCCCTGGATTAGAAACAATTTTGTACAAGCCCCTGGTGGTGAGTTTACAGTGTCCCCTAGAAACGCTCCAGGTGAGATACTATGGAGCGCGCCCTTGGGCCCTGATTTGAACCCCTATCTTTCTCATCTGTCCAGAATGTACAATGGTTATGCAGGTGGTTTTGAAGTGCAAGTAATTCTCGCGGGGAACGCGTTCACCGCCGGGAAAGTTATATTTGCGGCAGTTCCACCAAACTTTCCAACTGAAGGCTTAAGCCCCAGCCAGGTCACTATGTTCCCCCATATAATTGTAGATGTTAGGCAATTGGAACCTGTGTTGATCCCCCTACCTGATGTTAGGAATAATTTCTATCATTATAATCAATCACATGATTCTACCCTTAAGCTGATAGCAATGTTGTATACACCACTCAGAGCTAATAATGCCGGGGATGATGTCTTCACAGTCTCTTGTCGAGTTCTCACGAGGCCATCCCCCGATTTTGATTTTATATTCCTTGTGCCACCCACAGTTGAATCAAGAACCAAACCATTCACTGTCCCAATCTTAACTGTTGAGGAAATGTCTAATTCAAGATTCCCCATTCCTTTGGAAAAGTTATATACGGGTCCTAGCAGTGCTTTTGTTGTCCAACCACAAAATGGCAGATGTACGACTGATGGCGTGCTCTTAGGCACTACCCAGCTGTCAGCTGTCAATATCTGTAACTTCAGGGGGGATGTCACCCACATTGTGGGCAGCCATGATTATACAATGAATCTGGTATCCCAAAATTGGAGCAATTATGACCCAACAGAAGAAATCCCAGCCCCCCTGGGAACACCAGATTTCGTGGGAAAGATCCAAGGCCTGCTCACCCAGACCACAAGAGCGGATGGCTCGACCCGTGCCCACAAGGCTACAGTGAGCACTGGGAGCGTCCACTTCACTCCAAAGCTGGGTAGTGTTCAATTCACCACTGACACAAACAATGATTTCCAAACTGGCCAAAACACGAAATTCACACCAGTTGGCGTCATCCAAGACGGTGATCACCACCAGAATGAACCCCAACAATGGGTGCTCCCAAATTACTCAGGTAGAACTGGTCATAATGTGCACCTGGCCCCTGCCGTTGCCCCCACTTTTCCGGGTGAGCAACTCCTTTTCTTCAGGTCCACTATGCCCGGATGTAGCGGGTACCCCAACATGAACTTGGATTGTCTACTCCCCCAGGAATGGGTGCTGCACTTCTACCAGGAAGCAGCTCCAGCACAATCCGATGTGGCTCTGCTGAGATTTGTGAATCCAGACACAGGTAGGGTCCTGTTTGAGTGCAAGCTCCATAAGTCAGGCTATATCACAGTGGCTCACACCGGCCCGTATGATTTGGTTATCCCCCCCAATGGTTATTTTAGATTTGATTCCTGGGTCAACCAGTTCTACACACTTGCCCCCATGGGAAATGGAACGGGGCGCAGGCGTGCATTATAATGGCTG |
| AY502020.1 USA/2002/GII.4 Farmington Hills | ATGAAGACCCATCTGAAACAATGATTCCACACTCCCAAAGACCCATACAGTTGATGTCCTTACTGGGAGAGGCCGCACTCCACGGCCCAGCATTCTACAGCAAAATCAGCAAGTTAGTCATTGCAGAGCTAAAAGAAGGTGGCATGGATTTTTACGTGCCCAGGCAAGAGCCAATGTTCAGATGGATGAGATTCTCAGATCTGAGCACGTGGGAGGGCGATCGCAATCTGGCTCCCAGTTTTGTGAATGAAGATGGCGTCGAATGACGCCAACCCATCTGATGGGTCCACAGCCAACCTCGTCCCAGAGGTCAACAATGAGGTTATGGCTTTGGAGCCCGTTGTTGGTGCCGCTATTGCGGCACCTGTAGCGGGCCAACAAAATGTAATTGACCCCTGGATTAGAAATAATTTTGTACAAGCCCCTGGTGGAGAGTTTACAGTGTCACCTAGAAACGCTCCAGGTGAAATACTATGGAGCGCGCCCTTGGGCCCTGATCTGAATCCCTACCTTTCTCATTTGGCCAGAATGTACAATGGCTATGCAGGTGGCTTTGAAGTGCAGGTAATCCTTGCGGGGAATGCGTTCACCGCCGGGAAAATCATATTTGCAGCAGTCCCACCAAATTTCCCAACTGAAGGCTTGAGCCCCAGCCAGGTCACTATGTTCCCCCATATAATAGTAGATGTTAGGCAACTGGAACCTGTGTTGATCCCCTTACCTGATGTTAGGAACAATTTCTATCACTACAACCAATCAAATGACCCCACCATTAAATTGATAGCAATGCTGTACACACCACTTAGGGCTAATAATGCTGGGGAAGATGTCTTCACAGTCTCTTGTCGAGTCCTCACGAGGCCATCCCCCGATTTTGATTTCATATTTTTGGTGCCACCCACAGTTGAGTCAAGAACTAAACCATTCACCGTCCCAATTTTAACTGTTGAGGAAATGACCAATTCAAGATTCCCCATTCCTTTGGAAAAGTTGTTCACGGGTCCTAGCGGTGCCTTTGTTGTTCAACCACAAAATGGCAGGTGCACGACTGATGGCGTGCTCTTAGGCACTACCCAACTGTCTCCTGTCAACATCTGCACCTTCAGAGGGGATGTCACCCACATTGCAGGTACTCATAATTACACAATGAATTTGGCTTCTCAAAATTGGAACAATTATGACCCAACAGAAGAAATCCCAGCCCCTCTGGGGACCCCAGATTTCGTGGGAAGGATCCAAGGCATGCTCACCCAAACTACAAGGGGGGATGGCTCTACCCGCGGCCACAAAGCTACAGTGAGCACTGGGGATGTCCACTTCACTCCAAAGCTGGGCAGTATTCAGTTCAACACTGATACAAACAATGATTTTGAAACTGGCCAAAACACGAAATTCACCCCAGTCGGTGTTGTCCAGGATGGTAATGGTACCCACCAAAATGAACCTCAACAATGGGTGCTCCCAAGCTACTCAGGTAGAACTGGTCATAATGTACACCTAGCCCCCGCCGTAGCCCCCACTTTTCCGGGTGAGCAACTTCTTTTCTTCAGGTCCACTATGCCCGGATGCAGCGGGTACCCCAACATGAATTTGGATTGTCTACTCCCCCAGGAATGGGTGCAGCACTTCTACCAAGAGGCAGCTCCAGCACAATCTGATGTGGCTCTGCTAAGATTTGTGAATCCAGACACAGGTAGGGTTCTATTCGAGTGCAAGCTTCATAAATCAGGCTATGTCACAGTGGCTCACACTGGCCAGCATGATTTGGTTATCCCCCCCAATGGCTACTTTAGATTTGATTCTTGGGTCAACCAATTCTACACACTTGCCCCCATGGGAAATGGGACGGGGCGTAGACGTGCATTATAATGGCTG |
| JX445152.1 CAN/2004/GII.4 Farmington Hills | ATGAAGACCCATCTGAAACAATGATTCCACACTCCCAAAGACCCATACAGTTGATGTCCTTACTGGGAGAGGCCGCACTCCACGGCCCAACATTCTACAGCAAAATCAGCAAGTTAGTCATTGCAGAGCTAAAAGAAGGTGGCATGGATTTTTACGTGCCCAGGCAAGAGCCAATGTTCAGATGGATGAGATTCTCAGATCTGAGCACGTGGGAGGGCGATCGCAATCTGGCTCCCAGTTTTGTGAATGAAGATGGCGTCGAATGACGCCAACCCATCTGATGGGTCCACAGCCAACCTCGTCCCAGAGGTCAACAATGAGGTTATGGCTTTGGAGCCCGTTGTTGGTGCCGCTATTGCGGCACCTGTGGCGGGCCAACAAAATGTAATTGACCCCTGGATTAGAAATAATTTTGTACAGGCCCCTGGTGGAGAGTTTACAGTATCCCCTAGAAACGCTCCAGGTGAAATACTATGGAGCGCGCCCTTGGGCCCTGATCTGAATCCCTACCTTTCTCATTTGGCCAGAATGTACAATGGCTATGCAGGTGGCTTTGAAGTGCAGGTAATCCTTGCGGGGAATGCGTTCACCGCCGGGAAAATCATATTTGCAGCAGTCCCACCAAATTTCCCAACTGAAGGCTTGAGCCCCAGCCAGGTCACTATGTTCCCCCATATAATAGTAGATGTTAGGCAACTGGAACCTGTGTTGATCCCCTTACCTGATGTTAGGAACAATTTCTATCACTACAACCAGTCAAATGACCCCACCATTAAATTGATAGCAATGCTGTACACACCACTTAGGGCTAATAATGCTGGGGAAGATGTCTTCACAGTCTCTTGTCGAGTCCTCACGAGGCCATCCCCTGATTTTGATTTTATATTTTTGGTGCCACCCACAGTTGAGTCAAGAACTAAACCATTCACCGTCCCAATTTTAACTGTTGAGGAAATGACCAATTCAAGATTCCCTATTCCTTTGGAAAAGTTGTTCACGGGTCCTAGCGGTGCCTTTGTTGTTCAACCACAAAATGGCAGGTGCACGACTGATGGCGTACTCTTAGGCACCACCCAACTGTCTCCTGTCAACATCTGCACCTTCAGAGGGGATGTCACCCACATTGCAGGTACTCATAATTACACAATGAATTTGGCTTCTCAAAATTGGAACAATTATGACCCAACAGAAGAAATCCCAGCCCCTCTGGGGACCCCAGATTTCGTGGGAAGGATCCAAGGCATGCTCACCCAAACTACAAGGGGAGATGGCTCTACCCGCGGCCACAAAGCTACAGTGAGCACTGGGGATGTCCACTTCACTCCAAAGCTGGGCAGTATTCAATTCAACACTGATACAAACAATGATTTTGAAGCTGGCCAAAACACGAAATTCACCCCAGACGGTGTTGTCCAGGATGGTAATGGTACCCACCAAAATGAACCCCAACAATGGGTGCTCCCAAGCTACTCAGGTAGAACTGGTCATAATGTACACCTAGCCCCCGCCATAGCCCCCACTTTTCCGGGTGAGCAACTTCTTTTCTTCAGGTCCACTATGCCCGGATGCAGCGGGTACCCCAACATGAATTTGGATTGTCTACTCCCCCAAGAATGGGTGCAGCACTTCTACCAAGAGGCGGCTCCAGCACAATCTGATGTGGCTCTGCTAAGATTTGTGAATCCAGACACGGGTAGGGTTCTATTTGAGTGCAAGCTTCATAAATCAGGCTATGTCACAGTGGCTCACACTGGCCAGCATGATTTGGTTATCCCCCCCAATGGCTACTTTAGATTTGATTCCTGGGTCAACCAATTCTACACACTTGCCCCCATGGGAAATGGGACGGGGCGTAGACGTGCATTATAATGGCTG |
| EF684915.2 AUS/2006/GII.4 Den Haag | ATGAAGATCCATCTGAATCAATGATTCCACACTCTCAAAGACCCATACAATTGATGTCCTTACTGGGAGAGGCCGCACTCCACGGCCCAACATTCTACAGTAAAATCAGCAAATTAGTCATTGCAGAGCTAAAAGAAGGTGGTATGGATTTTTACGTGCCCAGGCAAGAGCCAATGTTCAGATGGATGAGATTCTCAGATCTGAGCACGTGGGAGGGCGATCGCAATCTGGCTCCCAGTTTTGTGAATGAAGATGGCGTCGAATGACGCCAACCCATCTGATGGGTCCGCAGCCAACCTCGTCCCAGAGGTCAACAATGAGGTTATGGCTTTGGAGCCCGTTGTCGGTGCCGCTATTGCGGCGCCTGTAGCGGGCCAACAAAATGTAATTGACCCCTGGATTAGAAATAATTTTGTACAAGCCCCTGGTGGAGAGTTCACAGTATCCCCTAGAAACGCTCCAGGTGAAATACTATGGAGCGCGCCCTTAGGCCCTGATCTGAATCCCTACCTATCTCATTTGGCCAGAATGTATAATGGTTATGCAGGTGGTTTTGAAGTGCAGGTGATCCTCGCGGGGAACGCGTTCACCGCCGGAAAAATTATATTTGCAGCAGTCCCACCAAATTTTCCAACTGAAGGCTTGAGCCCCAGTCAGGTCACTATGTTCCCCCACATAATAGTAGATGTTAGGCAATTGGAACCTGTGTTGATCCCCTTACCTGATGTTAGGAATAACTTCTATCATTATAATCAGTCAAATGATTCTACCATTAAATTGATAGCAATGCTGTATACACCACTTAGGGCTAATAATGCTGGGGAAGATGTCTTCACAGTCTCTTGTCGAGTCCTCACGAGGCCATCCCCTGATTTTGACTTTATATTTTTGGTGCCACCTACAGTTGAGTCAAGAACCAAACCATTTACTGTCCCAATCTTAACTGTTGAAGAAATGACCAATTCAAGATTCCCCATTCCTTTGGAAAAGTTATTCACGGGTCCCAGCGGTGCCTTTGTTGTTCAACCACAAAATGGCAGATGCACGACTGATGGCGTGCTCTTAGGCACTACCCAACTGTCTCCTGTCAACATCTGCACCTTCAGAGGGGATGTCACCCACATTGCAGGTTCTCGTAATTACACAATGAATTTGGCTTCTCTAAAATGGAACAAATATGACCCAACAGAAGAAATTCCAGCCCCTCTAGGAACTCCAGATTTCGTGGGAAAGATCCAAGGTGTGCTCACTCAAACCACAAAGGGAGATGGCTCGACCCGTGGCCATAAAGCTACAATTTACACTGGGAGTGCCCCCTTTACCCCAAAGCTGGGCAGTGTTCAATTCAGTACTGATACAGAAAATGATTTTGAAACTCACCAAAACACAAAATTCACCCCAGTCGGTGTTACCCAGGATGGTAGCACCACCCACCGAAATGAACCCCAACAATGGGTGCTCCCAAGTTACTCAGGTAGAAATGTCCATAATGTACACCTAGCCCCTGCTGTAGCCCCCACTTTTCCGGGTGAACAACTTCTTTTCTTCAGGTCCACTATGCCCGGATGCAGCGGGTATCCCAACATGGATTTGGATTGCCTACTCCCCCAGGAGTGGGTGCAGCACTTCTACCAAGAGGCAGCTCCAGCACAATCTGATGTGGCTCTATTGAGATTTGTGAATCCAGACACGGGTAGGGTCCTGTTTGAGTGCAAACTTCATAAATCAGGCTATGTCACAGTGGCTCACACCGGCCAGCATGACTTGGTCATCCCCCCCAATGGCTATTTTAGGTTTGATTCCTGGGTTAATCAATTCTACACACTTGCCCCCATGGGAAATGGAACGGGGCGTAGACGTGCTTTATAATGGCTG |
| JN400616.1 CHN/2008/GII.4 Den Haag | ATGAAGATCCATCTGAATCAATGATTCCACACTCTCAAAGACCCATACAGTTGATGTCCTTACTGGGAGAGGCCGCACTCCACGGCCCAACATTCTACAATAAAATCAGCAAATTAGTCATTGCAGAGTTAAAAGAAGGTGGTATGGATTTTTACGTGCCCAGGCAAGAGCCAATGTTCAGATGGATGAGATTCTCAGATCTGAGCACGTGGGAGGGCGATCGCAATCTGGCTCCCAGTTTTGTGAATGAAGATGGCGTCGAATGACGCCAACCCATCTGATGGGTCCGCAGCCAACCTCGTCCCAGAGGTCAACAATGAGGTTATGGCTTTGGAGCCCGTTGTCGGTGCCGCTATTGCGGCGCCTGTAGCGGGCCAACAAAATGTAATTGACCCCTGGATTAGAAATAATTTTGTACAAGCCCCTGGTGGAGAGTTCACAGTATCCCCTAGAAACGCTCCAGGTGAAATACTATGGAGCGCACCCTTAGGCCCTGATCTGAATCCCTACCTATCTCATTTGGCCAGAATGTATAATGGTTATGCAGGTGGTTTTGAAGTGCAGGTGATCCTCGCGGGGAACGCGTTCACCGCCGGAAAAATTATATTTGCAGCAGTCCCACCAAATTTTCCAACTGAAGGCTTGAGCCCCAGCCAGGTCACTATGTTCCCCCACATAATAGTGGATGTTAGGCAATTGGAACCTGTGTTGATCCCCTTACCTGATGTTAGGAATAACTTCTATCATTATAATCAATCAAATGATTCTACCATTAAGTTGATAGCAATGCTGTATACACCACTTAGGGCTAATAATGCTGGGGAAGATGTCTTCACAGTCTCTTGTCGAGTCCTCACGAGGCCATCCCCTGATTTTGATTTTATATTTTTGGTGCCACCTACAGTTGAGTCAAGAACTAAACCATTTACTGTCCCAATCTTGACTGTTGAGGAAATGACCAATTCAAGATTCCCCATTCCTTTGGAAAAGTTGTTCACGGGTCCCAGCAGTGCCTTTGTTGTTCAACCACAAAATGGCAGGTGCACGACTGATGGCGTGCTCTTAGGCACCACCCAACTGTCTCCTGTCAACATCTGCACCTTCAGAGGGGATGTCACCCACATTGCAGGTTCTCGTAATTACACAATGAATTTGGCTTCTCTAAATTGGAACAATTATGACCCAACAGAAGAAATTCCAGCCCCTCTAGGAACTCCAGATTTCGTGGGAAAGATCCAAGGTGTGCTCACTCAAACCACAAAGGGAGATGGTTCGACCCGTGGCCATAAAGCTACAGTTTACACTGGGAGTGCCCCCTTTACTCCAAAGCTGGGCAGTGTTCAATTCAGCACTGATACAGAAAATGATTTTGAAACTCACCAAAACACAAAATTCACCCCAGTCGGTGTCATCCAGGATGGTGGCACCACCCACCGAAATGAACCCCAACAATGGGTGCTCCCAAGTTATTCAGGTAGAGATGTCCCTAATGTGCACCTAGCCCCTGCTGTAGCCCCCACTTTCCCGGGTGAACAACTTCTTTTCTTTAGGTCTACTATGCCCGGATGCAGCGGGTATCCCAACATGGATTTGGATTGCCTACTCCCCCAGGAGTGGGTGCAGCACTTCTACCAAGAGGCAGCTCCAGCACAATCTGATGTGGCTCTATTGAGATTTGTGAATCCAGACACGGGTAGGGTCCTGTTTGAGTGCAAACTTCATAAATCAGGCTATGTCACAGTGGCTCACACCGGCCAGCATGATTTGGTCATCCCCCCCAATGGTTATTTTAGGTTTGATTCCTGGGTTAATCAATTCTACACACTTGCCCCCATGGGAAATGGAACGGGGCGTAGACGTGCTTTATAATGGCTG |
| KC894942.1 CHN/2011/GII.4 Den Haag | ATGAAGATCCATCTGAATCAATGATTCCACACTCTCAAAGACCCATACAATTGATGTCCTTACTGGGAGAGGCCGCACTCCACGGCCCAACATTCTACAACAAAATCAGCAAATTAGTCATTGCAGAGTTAAAAGAAGGTGGTATGGATTTTTACGTGCCCAGGCAAGAGCCAATGTTCAGATGGATGAGATTCTCAGATCTGAGCACGTGGGAGGGCGATCGCAATCTGGCTCCCAGTTTTGTGAATGAAGATGGCGTCGAATGACGCCAACCCATCTGATGGGTCCGCAGCCAACCTCGTCCCAGAGGTCAACAATGAGGTTATGGCTTTGGAGCCCGTTGTCGGTGCCGCTATTGCGGCGCCTGTAGCGGGCCAACAAAATGTAATTGACCCCTGGATTAGAAATAATTTTGTACAAGCCCCTGGTGGAGAGTTCACAGTATCCCCTAGAAACGCTCCAGGTGAAATACTATGGAGCGCACCCTTAGGCCCTGATCTGAATCCCTACCTATCTCATTTGGCCAGAATGTATAATGGTTATGCAGGTGGTTTTGAAGTGCAGGTGATCCTCGCGGGGAACGCGTTCACCGCCGGAAAAATTATATTTGCAGCAGTCCCACCAAATTTTCCAACTGAAGGCTTGAGTCCCAGCCAGGTCACTATGTTCCCCCACATAATAGTGGATGTTAGGCAATTGGAACCTGTGTTGATCCCCTTACCTGATGTTAGGAATAACTTCTATCACTATAATCAATCAAATGATTCTACCATTAAGTTGATAGCAATGCTGTATACACCACTTAGGGCTAATAATGCTGGGGAAGATGTCTTCACAGTCTCTTGTCGAGTCCTCACGAGGCCATCCCCTGATTTTGATTTCATATTTTTGGTGCCACCTACAGTTGAGTCAAGAACTAAACCATTTACTGTCCCAATCTTGACTGTTGAGGAAATGACCAATTCAAGATTCCCCATTCCTTTGGAAAAGTTGTTCACGGGTCCCAGCAGTGCCTTTGTTGTTCAACCACAAAATGGCAGATGCACGACTGATGGCGTGCTCTTAGGCACCACCCAACTGTCTCCTGTCAACATCTGCACCTTCAGAGGGGATGTCACCCACATTGCAGGTTCTCGTAATTACACAATGAATTTGGCTTCTCTAAATTGGAACAATTATGACCCAACAGAAGAAATTCCAGCTCCTCTAGGAACTCCAGATTTCGTGGGAAAGATCCAAGGTGTGCTCACTCAAACCACTAAGGGGGATGGTTCGACCCGTGGCCATAAAGCTACAGTTTACACTGGGAGTGCCCCCTTTACTCCAAAGCTGGGCAGTGTTCAATTCAGTACTGATACAGAAAATGATTTTGAAACTCACCAAAACACAAAATTCACCCCAGTCGGTGTCATCCAGGATGGTGGCACCACCCACCGAAATGAACCCCAACAATGGGTGCTCCCAAATTATTCAGGTAGTGATGTCCCTAATGTACACCTAGCCCCTGCTGTAGCCCCCAATTTCCCGGGTGAACAACTTCTTTTCTTTAGGTCTACTATGCCCGGATGCAGCGGGTATCCCAACATGGATTTGGATTGCCTACTCCCCCAGGAGTGGGTGCAGCACTTCTACCAAGAGGCAGCTCCAGCACAATCTGATGTGGCTCTATTGAGATTTGTGAATCCAGACACGGGTAGGGTCCTGTTTGAATGTAAACTCCATAAATCAGGCTATGTCACAGTGGCTCACACCGGCCAGCATGATTTGGTCATCCCCCCCAATGGTTATTTTAGGTTTGATTCCTGGGTTAATCAATTCTACACACTTGCCCCCATGGGAAATGGAACGGGGCGCAGACGTGCTTTATAATGGCTG |
| KC631814.1 USA/2011/GII.4 Yerseke | ATGAAGACCCATCTGAAACAATGATTCCACACTCCCAAAGACCCGTACAATTGATGTCCCTACTGGGGGAGGCCGCTCTTCACGGCCCAGCATTCTACAGCAAAATCAGCAAGTTAGTCATTGCAGAGCTAAAAGAAGGTGGCATGGATTTTTACGTGCCCAGACAAGAGCCAATGTTCAGATGGATGAGATTCTCAGATCTTAGCACGTGGGAGGGCGATCGCAATCTGGCTCCCAGTTTTGTGAATGAAGATGGCGTCGAGTGACGCCAACCCATCTGATGGGTCCACAGCCAACCTCGTCCCAGAGGTCAACAATGAGGTTATGGCTTTGGAGCCCGTTGTTGGTGCCGCTATTGCGGCACCTGTAGCGGGCCAACAAAATGTAATTGACCCCTGGATTAGAAATAATTTTGTACAAGCCCCTGGTGGAGAGTTTACAGTATCCCCTAGAAACGCTCCAGGTGAAATACTATGGAGCGCGCCCTTGGGCCCTGATTTGAATCCCTACCTTTCCCATTTGGCCAGAATGTACAATAGTTATGCAGGTGGTTTTGAAGTGCAGGTAATCCTCGCGGGGAACGCGTTCACCGCCGGAAAAATCATATTTGCAGCAGTCCCACCAAATTTCCCAACTGAAGGCTTGAGCCCCAGCCAGGTCACTATGTTCCCCCATATAATAGTAGATGTTAGGCAACTGGAACCTGTGTTGATCCCCTTACCCGATGTTAGGAATAATTTCTATCACTACAATCAATCAAATGACCCCACCATCAAATTGATAGCAATGTTGTATACACCACTTAGGGCTAATAATGCTGGGGACGATGTCTTCACAGTTTCTTGTCGGGTTCTCACGAGACCATCCCCCGATTTTGATTTCATATTTTTAGTGCCACCCACAGTTGAGTCAAGAACTAAACCATTCTCTGTCCCAATTTTAACTGTTGAGGAGATGACCAATTCAAGATTCCCCATTCCTTTGGAAAAGTTGTTCACGGGTCCCAGCAGTGCCTTTGTTGTTCAACCACAAAATGGCAGGTGCACGACTGATGGCGTGCTCCTAGGTACTACCCAACTGTCTCCTGTCAACATCTGCACCTTCAGAGGGGATGTCACCCACATTGCAGGTACTCAAGAATACACAATGAATCTGGCCTCCCAAAACTGGAACAATTATGATCCAACAGAAGAAATCCCAGCCCCTCTGGGAACTCCAGATTTCGTGGGTAAGATCCAAGGCGTGCTCACCCAAACCACAAGGAGGGATGGCTCGACCCGCGGTCACAAAGCCACAGTGAGCACTGGAAGTGTCCACTTCACCCCAAAGCTGGGCAGAATTCAATTCTCCACTGATACAAGCAATGATTTTGAAACTGGCCAAAACACGAGATTCACCCCAGTCGGTGTTGTCCAGGATGGTAGCACCACCCACCAAAATGAACCCCAACAATGGGTACTCCCAAATTATTCAGGTAGAGATAGCCACAATGTGCACCTAGCCCCTGCTGTGGCCCCCTCTTTTCCGGGTGAGCAACTTCTTTTCTTCAGGTCCACTATGCCCGGATGCAGCGGGTATCCCAACATGAATTTGGATTGCCTACTCCCCCAGGAGTGGGTGCAGCACTTCTACCAAGAGGCAGCCCCAGCACAATCTGATGTGGCTCTGCTAAGATTTGTGAATCCAGACACAGGTAGGGTTCTGTTTGAGTGTAAGCTTCATAAATCAGGCTATGTCACAGTGGCTCACACTGGCCAACATGATTTAGTTATCCCCCCCAATGGCTATTTTAGGTTTGATTCCTGGGTCAACCAGTTCTACACACTTGCCCCCATGGGAAATGGAACGGGGCGTAGACGTGCATTATAATGGCTG |
| JQ613571.1 AUS/2010/GII.4 New Orleans | ATGGAGACCCATCTGAAACAATGATTCCACACTCCCAAAGACCCATACAATTGATGTCCCTACTGGGGGAGGCCGCTCTCCACGGCCCAGCATTTTACAGCAAAATTAGCAAATTGGTCATTGCAGAGCTAAAAGAAGGTGGCATGGATTTTTACGTGCCCAGACAAGAGCCAATGTTCAGATGGATGAGATTCTCAGATCTGAGCACGTGGGAGGGCGATCGCAATCTGGCTCCCAGTTTTGTGAATGAAGATGGCGTCGAGTGACGCCAACCCATCTGATGGGTCCACAGCCAACCTCGTCCCAGAGGTCAACAATGAGGTTATGGCTTTGGAGCCCGTAGTTGGTGCCGCCATTGCGGCACCTGTAGCGGGCCAACAAAATGTAATTGACCCCTGGATTAGAAACAATTTTGTACAAGCCCCTGGTGGAGAGTTTACAGTATCCCCTAGAAACGCTCCAGGTGAAATACTATGGAGCGCGCCCTTAGGCCCTGATTTGAATCCCTACCTTTCCCATTTGGCCAGAATGTACAATGGTTATGCAGGTGGTTTTGAAGTGCAGGTAATCCTCGCGGGGAACGCGTTCACCGCCGGGAAAATCATATTTGCAGCAGTCCCACCAAATTTCCCAACTGAAGGTTTGAGCCCCAGCCAGGTCACTATGTTCCCCCATATAATAGTAGATGTTAGGCAATTGGAACCTGTGTTGATTCCCTTACCCGATGTTAGGAACAATTTCTACCATTATAATCAATCAAATGACCCCACCATCAAATTGATAGCAATGTTGTACACACCACTTAGGGCTAATAATGCCGGGGACGATGTCTTCACAGTTTCCTGTCGGGTTCTCACGAGACCATCCCCCGACTTTGATTTCATATTTTTGGTGCCACCCACAGTTGAATCAAGAACTAAACCATTCTCTGTCCCAGTTTTAACTGTTGAGGAGATGACCAATTCAAGGTTCCCCATTCCTTTGGAAAAGTTGTTCACAGGCCCCAGTAGTGCCTTTGTTGTTCAACCACAAAACGGCAGGTGCACGACTGATGGCGTGCTCCTAGGTACTACCCAACTGTCTCCTGTCAACATCTGCACCTTCAGAGGGGATGTCACCCATATCCCAGGCAGTCGTAACTACACAATGAATTTGGCCTCCCAAAATTGGAACAGTTACGACCCAACAGAAGAAATCCCAGCCCCTCTAGGAACTCCAGATTTCGTGGGGAAGATTCAAGGTGTGCTCACCCAAACTACAAGGACAAATGGCTCGACCCGCGGCCACAAAGCTACAGTGTACACTGGGAGCGCCGACTTTTCTCCAAAACTGGGTAGAGTTCAATTTGCCACTGACACAGACAATGATTTTGAAACTAACCAAAACACAAAGTTCACCCCAGTCGGTGTTATCCAGGATGGTAGTACTACCCCCCGAAATGAACCCCAACAATGGGTGCTCCCAAGTTACTCAGGCAGGAACATCCATAATGTGCACCTGGCCCCCGCTGTAGCCCCCACTTTCCCGGGCGAACAGCTCCTCTTCTTCAGATCTACTATGCCCGGATGCAGCGGGTACCCCAACATGGATTTGGACTGTCTGCTCCCCCAGGAATGGGTGCAGTATTTCTACCAGGAGGCAGCCCCAGCACAATCTGATGTGGCTCTGTTAAGATTTGTGAATCCAGACACAGGTAGGGTTTTGTTTGAGTGTAAGCTCCATAAATCAGGCTATGTTACAGTGGCTCACACTGGCCAACATGATTTGGTTATCCCCCCCAATGGTTATTTTAGATTTGATTCCTGGGTCAACCAGTTCTACACACTTGCCCCCATGGGAAATGGGACGGGGCGTAGACGTGCATTATAATGGCTG |
| KP784695.1 ZAF/2012/GII.4 New Orleans | ATGGAGACCCATCTGAAACAATGATTCCACACTCCCAAAGACCCATACAATTGATGTCCCTATTGGGGGAGGCCGCTCTCCACGGCCCAGCATTTTACAGCAAAATCAGTAAATTGGTCATTGCAGAGCTAAAAGAAGGTGGTATGGATTTTTACGTGCCCAGACAAGAGCCAATGTTCAGATGGATGAGATTCTCAGATCTGAGCACGTGGGAGGGCGATCGCAATCTGGCTCCCAGTTTTGTGAATGAAGATGGCGTCGAGTGACGCCAACCCATCTGATGGGTCCACAGCCAACCTCGTCCCAGAGGTCAACAATGAGGTTATGGCTTTGGAGCCCGTGGTTGGTGCCGCTATTGCGGCACCTGTAGCGGGCCAGCAAAATGTAATTGACCCCTGGATTAGAAACAATTTTGTACAAGCCCCTGGTGGAGAGTTTACAGTATCCCCTAGAAACGCTCCAGGTGAAATACTATGGAGCGCGCCCTTAGGCCCTGATCTGAACCCCTACCTTTCCCATTTGGCCAGAATGTACAATGGTTATGCAGGTGGTTTTGAAGTGCAGGTAATCCTCGCGGGGAATGCGTTCACCGCCGGGAAAATCATATTTGCAGCAGTCCCACCAAATTTCCCAACTGAAGGTTTGAGCCCCAGCCAAGTCACTATGTTCCCCCACATAATAGTAGATGTTAGGCAATTGGAACCTGTGTTGATCCCCTTACCCGATGTTAGGAATAATTTCTACCATTATAATCAGTCAAATGACTCCACCATCAAATTGATAGCAATGTTGTACACACCACTTAGGGCTAACAATGCCGGGGACGATGTCTTCACAGTCTCTTGTCGAGTTCTCACGAGACCATCCCCCGATTTTGATTTCATATTTTTGGTGCCACCCACAGTTGAATCAAGAACTAAACCATTTTCTGTCCCAGTTTTAACTGTTGAGGAGATGACCAATTCAAGGTTCCCCATTCCTTTGGAAAAGTTGTTCACGGGTCCCAGTAGTGCCTTTGTTGTTCAACCACAAAATGGCAGGTGCACGACTGATGGCGTGCTCCTAGGTACTACCCAACTGTCTCCTGTCAACATCTGCACCTTCAGAGGGGATGTCACCCACATTTCAGGCAGTCGTAACTACACAATGAATTTGGCCTCCCAAAATTGGAACAGTTACGATCCAACAGAAGAAATCCCGGCCCCCCTAGGAACTCCAGATTTCGTGGGGAAGATCCAAGGTGTGCTCACCCAGACCACAAGGACAGATGGCTCGACCCGCGGCCACAAAGCTACAGTGTACACTGGGAGCGCCGACTTTTCTCCAAAACTGGGTAGGGTTCAATTTGCTACTGACACAGACAATGATTTTGTAACTAATCAAAACACAAAGTTCACCCCAGTCGGTGTTATCCAGGATGGTAATACTACCCCCCGAAATGAACCCCAACAATGGGTGCTCCCAAGTTACTCAGGTAGAAACACTCATAATGTGCACCTGGCCCCCGCTGTAGCCCCCACTTTCCCGGGCGAGCAGCTCCTCTTCTTCAGATCTACCATGCCCGGATGCAGCGGGTACCCCAACATGGATTTGGACTGTCTGCTCCCCCAGGAATGGGTGCAGTATTTCTACCAGGAGGCAGCCCCAGCACAATCTGATGTGGCACTATTAAGATTTGTGAATCCAGACACAGGTAGGGTTTTGTTTGAGTGTAAGCTTCATAAATCAGGCTATGTTACAGTGGCTCACACTGGCCAACATGATTTAGTTATCCCCCCCAATGGTTATTTTAGATTTGATTCCTGGGTCAACCAGTTCTACACACTTGCCCCCATGGGAAATGGGACGGGGCGTAGACGTGCATTATAATGGCTG |
| MT026582.1 USA/2019/GII.4 Sydney(P4) | ATGGAGACCCATCTGAAACGATGATTCCACATTCCCAAAGACCCATACAATTGATGTCCCTACTGGGGGAGGCCGCTCTCCACGGCCCAGCATTTTACAGTAAAATTAGCAAATTGGTCATTGCAGAGCTAAAAGAAGGTGGTATGGATTTTTACGTGCCCAGACAAGAGCCAATGTTCAGATGGATGAGATTCTCAGATCTGAGCACGTGGGAGGGCGATCGCAATCTGGCTCCCAGTTTTGTGAATGAAGATGGCGTCGAGTGACGCCAACCCATCTGATGGGTCCGCAGCCAACCTCGTCCCAGAGGTCAACAATGAGGTTATGGCTCTGGAGCCCGTTGTTGGTGCCGCTATTGCGGCACCTGTAGCGGGCCAACAAAATGTAATTGACCCCTGGATTAGAAACAATTTTGTACAAGCCCCTGGTGGAGAGTTTACAGTGTCCCCCAGAAACGCTCCAGGTGAAATACTATGGAGCGCACCCTTAGGCCCTGATTTAAATCCCTACCTATCCCACTTGGCCAGAATGTATAATGGTTATGCAGGTGGTTTTGAAGTGCAGGTGATTCTCGCGGGGAACGCGTTCACCGCCGGGAAGGTCATATTTGCAGCAGTCCCACCAAATTTTCCAACTGAAGGCTTGAGCCCCAGCCAGGTCACTATGTTCCCCCATATAGTAGTGGATGTTAGGCAACTAGAACCTGTGTTGATTCCCTTACCCGATGTTAGGAATAATTTCTACCATTACAATCAATCAAATGATCCCACCATTAAGTTGATAGCAATGTTATATACACCACTTAGGGCTAATAATGCTGGGGATGATGTCTTCACAGTTTCTTGCCGAGTTCTCACGAGGCCATCCCCCGATTTTGATTTCATATTTCTAGTGCCACCCACAGTTGAGTCTAGAACCAAACCATTTTCTGTCCCAGTTTTAACTGTTGAGGAGATGACCAATTCAAGATTCCCCATTCCTTTGGAAAAGTTGTTCACGGGTCCCAGCAGTGCCTTTGTTGTTCAACCACAAAACGGTAGGTGCACGACTGATGGCGTGCTCCTAGGCACCACCCAATTGTCTCCTGTCAACATCTGCACCTTCAGAGGGGATGTCACCCACATTGCAGGTAGTCGTAACTATACAATGAATTTGGCTTCTCAAAATTGGAACAATTATGACCCAACAGAAGAAATCCCAGCCCCTCTAGGAGCTCCAGATTTTGTGGGGAAGATTCAAGGCATGCTCACCCAAACCACAAGGGCAGATGGCTCAACACGCGGCCACAAGGCTACGGTGTACACTGGGAGCGCCGACTTCGCTCCAAAACTGGGCAGAGTTCAATTTGAAACTGACACAGACCATGATTTTGAAGCTAACCAAAACACAAAGTTCACCCCAGTCGGTGTCATCCAAGATGGTAGCACCACCCACCGAAATGAACCCCAACAGTGGGTGCTCCCAAGTTACTCAGGCAGAAATACTCATAATGTTCATCTGGCCCCCGCTGTGGCCCCCACTTTTCCGGGTGAACAACTTCTCTTCTTTAGATCCACCATGCCCGGATGTAGCGGGTATCCCAACATGGATTTGGACTGTTTGCTCCCCCAGGAATGGGTGCAGTACTTCTACCAAGAGGCAGCCCCAGCACAATCTGATGTGGCCCTGCTAAGATTTGTGAATCCAGACACAGGTAGGGTTTTGTTTGAATGTAAACTCCATAAATCAGGCTATGTCACAGTGGCTCACACTGGCCAACATGATTTGGTCATCCCCCCCAATGGTTATTTTAGGTTTGATTCCTGGGTCAACCAGTTCTACACGCTTGCCCCCATGGGAAATGGAACGGGGCGTAGACGCGTAGTATAATGGCTG |
| JX459908.1 AUS/2012/GII.4 Sydney(P31) | ATGAAGATCCATTTGAAACAATGATACCACACTCCCAAAGACCCATACAATTGATGTCCTTGCTGGGCGAGGCTGCACTCCACGGCCCGGCATTCTATAGCAAAATTAGCAAATTAGTCATTGCAGAGTTGAAGGAAGGTGGCATGGATTTTTACGTGCCCAGACAAGAGCCAATGTTCAGATGGATGAGATTCTCAGATCTGAGCACGTGGGAGGGCGATCGCAATCTGGCTCCCAGTTTTGTGAATGAAGATGGCGTCGAGTGACGCCAACCCATCTGATGGGTCCGCAGCCAACCTCGTCCCAGAGGTCAACAATGAGGTTATGGCTCTGGAGCCCGTTGTTGGTGCCGCCATTGCGGCACCTGTAGCGGGCCAACAAAATGTAATTGACCCCTGGATTAGAAATAATTTTGTACAAGCCCCTGGTGGAGAGTTTACAGTATCCCCTAGAAACGCTCCAGGTGAAATACTATGGAGCGCGCCCTTGGGCCCTGATCTAAATCCCTACCTATCCCATTTGGCCAGAATGTACAATGGTTATGCAGGTGGTTTTGAAGTGCAGGTAATTCTCGCGGGGAACGCGTTCACCGCCGGGAAGGTCATATTTGCAGCAGTCCCACCAAATTTTCCAACTGAAGGCTTGAGCCCCAGCCAGGTCACTATGTTCCCCCATATAGTAGTAGATGTTAGGCAACTAGAACCTGTGTTGATTCCCTTACCCGATGTTAGGAATAATTTCTATCATTATAATCAATCAAATGACCCTACCATTAAGTTGATAGCAATGTTGTATACACCACTTAGGGCTAATAATGCTGGGGATGATGTCTTCACAGTTTCTTGCCGAGTTCTCACGAGACCATCCCCCGATTTTGATTTCATATTTTTAGTGCCACCCACAGTTGAGTCAAGAACTAAACCATTCTCTGTCCCAGTTTTAACTGTTGAGGAGATGACCAATTCAAGATTCCCCATTCCTTTGGAAAAGTTGTTCACGGGTCCCAGCAGTGCCTTTGTTGTCCAACCACAAAACGGCAGGTGCACGACTGATGGCGTGCTCCTAGGCACCACCCAACTGTCTCCTGTCAACATCTGCACCTTCAGAGGAGATGTCACCCACATCACAGGTAGTCGTAACTACACAATGAATTTGGCTTCTCAAAATTGGAACGATTATGACCCAACAGAAGAAATCCCAGCCCCTCTAGGAACTCCAGATTTTGTGGGGAAGATTCAAGGCGTTCTCACCCAAACCACAAGGACAGATGGCTCAACACGCGGCCACAAAGCTACAGTGTACACTGGGAGCGCCGACTTTGCTCCAAAACTGGGTAGAGTTCAATTTGAAACTGACACAGACCGTGATTTTGAAGCTAACCAAAACACAAAGTTCACCCCAGTCGGTGTCATCCAAGATGGTGGCACCACCCACCGAAATGAACCCCAACAGTGGGTGCTCCCAAGCTACTCAGGCAGAAATACTCATAATGTGCATCTGGCCCCCGCTGTAGCCCCCACTTTTCCGGGTGAGCAACTTCTCTTCTTCAGATCCACCATGCCCGGATGCAGCGGGTACCCCAACATGGATTTGGACTGTCTGCTCCCCCAGGAATGGGTGCAGTACTTCTACCAAGAGGCAGCCCCAGCACAATCTGATGTGGCTCTGCTAAGATTTGTGAATCCAGACACAGGTAGGGTTTTGTTTGAGTGTAAGCTTCATAAATCAGGCTATGTTACAGTGGCTCACACTGGCCAACATGATTTGGTTATCCCCCCCAATGGTTATTTTAGGTTTGATTCCTGGGTCAACCAGTTTTACACGCTTGCCCCCATGGGAAATGGAACGGGGCGTAGACGTGCAGTATAATGGCTG |
| KM258128.1 CHN/2012/GII.4 Sydney(P31) | ATGAAGATCCATTTGAAACAATGATACCACACTCCCAAAGACCCATACAATTGATGTCCTTGCTGGGCGAGGCTGCACTCCACGGCCCGGCATTCTATAGCAAAATTAGCAAATTAGTCATTGCAGAGTTGAAGGAAGGTGGCATGGATTTTTACGTGCCCAGACAAGAGCCAATGTTCAGATGGATGAGATTCTCAGATCTGAGCACGTGGGAGGGCGATCGCAATCTGGCTCCCAGTTTTGTGAATGAAGATGGCGTCGAGTGACGCCAACCCATCTGATGGGTCCGCAGCCAACCTCGTCCCAGAGGTCAACAATGAGGTTATGGCTCTGGAGCCCGTTGTTGGTGCCGCCATTGCGGCGCCTGTAGCGGGCCAACAAAATGTAATTGACCCCTGGATTAGAAATAATTTTGTACAGGCCCCTGGTGGAGAGTTTACAGTATCCCCTAGAAACGCTCCAGGTGAAATACTATGGAGCGCGCCCTTGGGCCCTGATCTAAATCCCTACCTATCCCATTTGGCCAGAATGTACAATGGTTATGCAGGTGGTTTTGAAGTGCAGGTAATTCTCGCGGGGAACGCGTTCACCGCCGGGAAGGTCATATTTGCAGCAGTCCCACCAAATTTTCCAACTGAAGGCTTGAGCCCCAGCCAGGTCACTATGTTCCCCCATATAGTAGTAGATGTTAGGCAACTAGAACCTGTGTTGATTCCCTTACCCGATGTTAGGAATAATTTCTATCATTACAATCAATCAAATGACCCCACCATTAAGTTGATAGCAATGTTGTATACACCACTTAGGGCTAATAATGCTGGGGATGATGTCTTCACAGTTTCTTGCCGAGTTCTCACGAGACCATCCCCCGATTTTGATTTCATATTTCTAGTGCCACCCACAGTTGAGTCAAGAACTAAACCATTCTCTGTCCCAGTTTTAACTGTTGAGGAGATGACCAATTCAAGATTTCCCATTCCTTTGGAAAAGTTGTTCACGGGTCCCAGCAGTGCCTTTGTTGTCCAACCACAAAACGGCAGGTGCACGACTGATGGCGTGCTCCTAGGCACCACCCAACTGTCTCCTGTCAACATCTGCACCTTCAGAGGAGATGTCACCCATATCACAGGTAGTCGTAACTACACAATGAATTTGGCTTCTCAAAATTGGAACAATTATGACCCAACAGAAGAAATCCCAGCCCCTCTAGGAACTCCAGATTTTGTGGGGAAGATTCAAGGCGTGCTCACCCAAACCACAAGGACAGATGGCTCAACACGCGGCCACAAAGCTACAGTGTACACTGGGAGCGCCGACTTTGCTCCAAAACTGGGTAGAGTTCAATTTGAAACTGACACAGACCATGATTTTGAAGCTAACCAAAACACAAAGTTCACCCCAGTCGGTGTCATCCAAGATGGTGGCACCACCCACCGAAATGAACCCCAGCAGTGGGTGCTCCCAAGTTACTCAGGCAGAAACACTCATAATGTGCATCTGGCCCCCGCTGTAGCCCCCACTTTTCCGGGTGAGCAACTTCTCTTCTTCAGGTCCACCATGCCCGGATGCAGCGGGTACCCCAACATGGATTTGGACTGTCTGCTCCCCCAGGAATGGGTGCAGTACTTCTACCAAGAGGCAGCCCCAGCACAATCTGATGTGGCTCTGCTAAGATTTGTGAATCCAGACACAGGTAGGGTTTTGTTTGAGTGTAAGCTTCATAAATCAGGCTATGTTACAGTGGCTCACACTGGCCAATATGATTTGGTTATTCCCCCCAATGGTTATTTTAGGTTTGATTCCTGGGTCAACCAGTTTTACACGCTTGCCCCCATGGGAAATGGAACGGGGCGTAGACGTGCAGTATAATGGCTG |
| KT202793.1 CHN/2013/GII.4 Sydney(P31) | ATGAAGATCCATTTGAAACAATGATACCACACTCCCAAAGACCCATACAATTGATGTCCTTGCTGGGCGAGGCTGCACTCCACGGCCCGGCATTCTATAGCAAAATTAGCAAATTAGTCATTGCAGAGTTGAAGGAAGGTGGCATGGATTTTTACGTACCCAGACAAGAGCCAATGTTCAGATGGATGAGATTCTCAGATCTGAGCACGTGGGAGGGCGATCGCAATCTGGCTCCCAGTTTTGTGAATGAAGATGGCGTCGAGTGACGCCAACCCATCTGATGGGTCCGCAGCCAACCTCGTCCCAGAGGTCAACAATGAGGTTATGGCTCTGGAGCCCGTTGTTGGTGCCGCCATTGCGGCACCTGTAGCGGGCCAACAAAATGTAATTGACCCCTGGATTAGAAATAATTTTGTACAAGCCCCTGGTGGAGAATTTACAGTATCCCCTAGAAACGCTCCAGGTGAAATACTATGGAGCGCGCCCTTGGGCCCTGATCTAAATCCCTACCTATCCCATTTGGCCAGAATGTACAATGGTTATGCAGGTGGTTTTGAAGTGCAGGTAATTCTCGCGGGGAACGCGTTCACCGCCGGGAAGGTCATATTTGCAGCAGTCCCACCAAATTTTCCAACTGAAGGCTTGAGCCCCAGCCAGGTCACTATGTTCCCCCATATAGTAGTAGATGTTAGGCAACTAGAACCTGTGTTGATTCCCTTACCCGATGTTAGGAATAATTTCTATCATTACAATCAATCAAATGACCCCACCATTAAGTTGATAGCAATGTTGTATACACCACTTAGGGCTAATAATGCTGGGGATGATGTCTTCACAGTCTCTTGCCGAGTTCTCACGAGACCATCCCCCGATTTTGATTTCATATTTCTAGTGCCACCCACAGTTGAGTCAAGAACTAAACCATTCTCTGTCCCAGTTTTAACTGTTGAGGAGATGACCAATTCAAGATTCCCCATTCCTTTGGAAAAGTTGTTCACGGGTCCCAGCAGTGCCTTTGTTGTCCAACCACAAAACGGTAGGTGCACGACTGATGGCGTGCTCCTAGGCACCACCCAACTGTCTCCTGTCAACATCTGCACCTTCAGAGGAGATGTCACCCATATCACAGGTAGTCGTAACTACACAATGAATTTGGCTTCTCAAAATTGGAGCAATTATGACCCAACAGAAGAAATCCCAGCCCCTCTAGGAACTCCAGATTTTGTGGGGAAGATTCAAGGCGTGCTCACCCAAACTACAAGGACAGATGGCTCAACACGCGGCCACAAAGCCACAGTGTACACTGGGAGCGCCGACTTTGCTCCAAAACTGGGTAGAGTTCAATTTGAAACTGACACAGACCGTGATTTTGAAGCTAACCAAAACACAAAGTTCACCCCAGTTGGTGTCATCCAAGATGGTGGCACCACCCACCGAAATGAACCCCAACAGTGGGTGCTCCCAAGTTACTCAGGCAGAAATACTCCTAATGTGCATCTGGCCCCCGCTGTAGCCCCCACTTTTCCGGGTGAGCAACTTCTCTTCTTCAGATCCACCATGCCCGGATGCAGCGGGTACCCCAACATGGATTTGGACTGTCTGCTCCCCCAGGAATGGGTGCAGTACTTCTACCAAGAGGCAGCCCCAGCACAATCTGATGTGGCTCTGCTAAGATTTGTGAATCCAGACACAGGTAGGGTTTTGTTTGAGTGTAAGCTTCATAAATCAGGCTATGTTACAGTGGCTCACACTGGCCAACATGATTTGGTTATCCCCCCCAATGGTTATTTTAGGTTTGATTCCTGGGTCAACCAGTTTTACACGCTTGCCCCCATGGGAAATGGAACGGGGCGTAGACGTGCACTATAATGGCTG |
| MW661254.1 CAN/2016/GII.4 Sydney(P31) | ATGAAGACCCCAATGAGACAATGATACCCCATTCTCAAAGACCCATACAGCTCATGGCACTGCTTGGTGAAGCCTCTCTTCACGGACCCTCTTTCTACAGTAAAATCAGTAAATTGGTCATAACTGAACTCAAAGAAGGTGGGATGGACTTTTACGTGCCAAGGCAGGAACCCATGTTCAGGTGGATGAGGTTTTCTGACTTGAGCACGTGGGAGGGCGATCGCAATCTGGCTCCCAATTTTGTGAATGAAGATGGCGTCGAGTGACGCCAACCCATCTGATGGGTCCGCAGCCAACCTCGTACCAGAGGTCAACAATGAGGTTATGGCTTTGGAGCCCGTTGTTGGTGCCGCTATTGCGGCACCTGTAGCGGGCCAACAAAATGTAATTGACCCCTGGATTAGAAATAATTTTGTACAAGCCCCTGGTGGGGAGTTTACAGTATCCCCTAGAAACGCTCCAGGTGAAATACTATGGAGCGCGCCCCTAGGCCCTGACCTAAATCCCTACCTATCCCATTTGGCCAGAATGTACAATGGTTATGCAGGTGGTTTTGAAGTGCAGGTAATTCTCGCGGGGAACGCGTTCACCGCCGGGAAGATCATATTTGCAGCAGTCCCACCAAATTTTCCAACTGAAGGCTTAAGTCCTAGCCAGGTCACTATGTTCCCCCACATAATAGTAGATGTTAGACAATTAGAACCTGTGCTGATTCCCTTACCCGATGTCAGGAATAATTTCTATCATTACAATCAGTCAAATGACTCCACCATTAAGTTGATAGCAATGTTGTATACACCACTTAGGGCTAATAATGCTGGGGATGATGTTTTCACAGTTTCGTGCCGAGTTCTCACGAGACCATCCCCCGATTTTGATTTTATATTTTTAGTGCCACCCACAGTTGAGTCAAGAACTAAACCATTCTCTGTCCCAGTTTTAACTGTTGAGGAGATGACCAATTCAAGATTCCCCATTCCTTTGGAAAAGTTGTTCACGGGCCCCAGCAGTGCCTTTGTTGTTCAACCACAAAACGGCAGGTGCACAACTGATGGCGTGCTCCTAGGCACCACCCAACTGTCTCCTGTCAACATCTGCACCTTCAGAGGGGATGTCACCCACATCACAGGTAGTCGCAACTACACAATGAATTTGGCTTCTCAAAATTGGAACAACTATGACCCAACAGAAGAAATCCCAGCCCCTCTAGGAACTCCGGATTTTGTGGGGAAGATTCAAGGCATGCTCACCCAAACCACAAGAACAGATGGTTCAACGCGCGGCCACAAAGCTACAGTGTACACTGGGAGCGCCGACTTTGCTCCAAAACTGGGTAGAGTTCAATTTGAAACTGACACAGACCATGATTTTGAAGCTAATCAAAACACAAAGTTCACCCCAGTCGGTGTCATCCAAGATGGTAGCACCACCCACCGGAACGAACCCCAACAGTGGGTGCTCCCAAGTTACTCAGGCAGAAATACTCACAATGTACATCTGGCCCCCGCTGTAGCCCCCACCTTTCCGGGTGAGCAACTTCTCTTCTTCAGATCCACCATGCCCGGATGCAGCGGGTACCCCAACATGGATTTGGACTGTCTGCTCCCCCAGGAATGGGTGCAGTACTTCTACCAAGAGGCAGCCCCAGCACAATCTGATGTGGCTCTGCTAAGATTTGTGAATCCAGACACAGGTAGGGTTTTGTTTGAGTGCAAGCTTCACAAATCAGGCTATGTTACAGTGGCTCACACTGGCCAACATGATTTGGTTATCCCCCCCAATGGTTACTTTAGATTTGATTCCTGGGTCAACCAGTTCTACACGCTTGCCCCCATGGGAAATGGAACGGGGCGTAGACGTGTAGTATAATGGCTG |
| MG214988.1 CHN/2017/GII.4 Sydney(P31) | ATGAAGATCCATTTGAAACAATGATACCACACTCCCAGAGACCCATACAATTGATGTCCTTGCTGGGCGAGGCTGCACTCCACGGCCCGGCATTCTACAGCAAAATTAGCAAATTAGTCATTGCAGAGTTGAAGGAAGGTGGCATGGATTTTTACGTACCCAGACAAGAGCCAATGTTCAGATGGATGAGATTCTCAGATCTGAGCACGTGGGAGGGCGATCGCAATCTGGCTCCCAGTTTTGTGAATGAAGATGGCGTCGAGTGACGCCAACCCATCTGATGGGTCCGCAGCCAACCTCGTCCCAGAGGTCAACAATGAGGTTATGGCTCTGGAGCCCGTTGTTGGTGCCGCCATTGCGGCACCTGTAGCGGGCCAACAAAATGTAATTGACCCCTGGATTAGAAACAATTTTGTACAAGCCCCTGGTGGAGAATTTACAGTATCCCCTAGAAACGCTCCAGGTGAAATACTATGGAGCGCGCCCTTGGGCCCTGATCTAAATCCCTACTTGTCTCATTTGGCCAGAATGTACAATGGTTATGCAGGTGGTTTTGAAGTGCAGGTAATTCTCGCGGGGAACGCGTTCACCGCCGGGAAGGTCATATTTGCAGCAGTCCCACCAAATTTTCCAACTGAAGGCTTGAGCCCCAGCCAGGTCACTATGTTCCCCCATATAGTAGTAGATGTTAGGCAACTAGAACCTGTGTTGATTCCCTTACCCGATGTTAGGAATAATTTCTATCATTACAATCAATCAAATGACCCCACCATTAAGTTGATAGCAATGTTGTATACACCACTTAGGGCTAATAATGCTGGGGATGATGTCTTCACAGTTTCTTGCCGAGTTCTCACGAGACCATCCCCCGATTTTGACTTCATATTTCTAGTGCCACCCACAGTTGAGTCAAGAACTAAACCATTCTCTGTCCCAGTTTTGACTGTTGAGGAGATGACCAATTCAAGATTCCCCATTCCTTTGGAAAAGTTGTTCACGGGTCCCAGCAGTGCCTTTGTCGTCCAACCACAAAACGGTAGGTGCACGACTGATGGCGTGCTCCTAGGCACCACCCAACTGTCTCCTGTCAACATCTGCACCTTCAGAGGAGATGTCACCCATGTTACAGGTAGTCATAACTATACAATGAATTTGGCTTCTCAAAATTGGAGCAACTACGACCCAACAGAAGAAATCCCAGCCCCTCTAGGGACTCCAGACTTTGTGGGGAAGATTCAAGGCATGCTTACCCAAACCACAAGGACAGATGGCTCAACACGCGGCCACAAAGCCACAGTGTACACTGGGAGCGCCGACTTTGCTCCAAAACTGGGTAGAGTTCAATTTGAAACTGACACAAACAATGATTTTGAAGCTAACCAAAACACAAAGTTCACCCCAGTTGGTGTCATCCAAGATGGTGGCACCACCCACCGCAATGAACCCCAACAGTGGGTGCTCCCAAGTTACTCAGGCAGGAACACTCCTAATGTGCATCTGGCCCCCGCTGTAGCCCCCACTTTTCCGGGTGAGCAACTCCTCTTCTTCAGATCCACCATGCCCGGATGCAGCGGGTACCCCAACATGGATTTGGATTGTCTGCTCCCCCAGGAATGGGTGCAGTACTTCTACCAGGAGGCAGCCCCAGCACAATCTGATGTGGCTCTGCTAAGATTTGTGAATCCAGACACAGGTAGGGTTTTGTTTGAATGTAAGCTTCATAAATCAGGCTATGTTACAGTGGCTCACACTGGCCAACATGATTTGGTTATCCCCCCCAATGGTTATTTTAGATTTGATTCCTGGGTCAACCAGTTTTACACGCTTGCCCCCATGGGAAATGGAGCGGGGCGTAGACGTGCACTATAATGGCTG |
| MW559994.1 KEN/2019/GII.4 Sydney(P31) | ATGAAGATCCATCTGAAACAATGATACCACACTCCCAAAGACCCATACAATTGATGTCCTTGCTAGGCGAGGCTGCACTCCACGGCCCCGCATTTTATAGCAAAATTAGCAAATTGGTTATTGCAGAGTTGAAGGAAGGTGGCATGGATTTTTACGTGCCCAGACAAGAGCCAATGTTCAGATGGATGAGATTCTCAGATCTGAGCACGTGGGAGGGCGATCGCAATCTGGCTCCCAGTTTTGTGAATGAAGATGGCGTCGAGTGACGCCAACCCATCTGATGGGTCCGCAGCCAACCTCGTCCCAGAGGTCAACAATGAGGTTATGGCTCTGGAGCCCGTTGTTGGTGCCGCCATAGCGGCACCTGTAGCGGGCCAGCAAAATGTAATTGACCCCTGGATTAGAAATAATTTTGTACAAGCCCCTGGTGGAGAGTTTACAGTATCTCCTAGAAACGCTCCAGGTGAAATACTATGGAGCGCGCCCTTAGGCCCTGATTTAAATCCCTACCTATCCCATTTGGCCAGAATGTACAATAGTTATGCAGGTGGCTTTGAAGTGCAGGTAATTCTCGCGGGGAACGCGTTCACCGCCGGGAAGGTCATATTTGCAGCAGTCCCACCAAATTTTCCAACTGAAGGCTTAAGTCCCAGCCAGGTCACCATGTTCCCCCATATAGTAGTAGATGTTAGGCAACTAGAACCTGTGTTGATTCCCTTACCCGATGTTAGGAACAATTTTTACCACTACAATCAATCAAATGACCCCACTATCAAGTTGATAGCAATGTTGTATACACCACTTAGAGCTAATAATGCTGGGGATGATGTCTTCACAGTTTCTTGCCGGGTCCTCACGAGACCATCCCCCGATTTTGATTTCATATTTTTGGTGCCACCCACAGTTGAGTCAAGAACTAAGCCATTCTCTATCCCAGTTTTAACTGTTGAGGAGATGACCAATTCAAGATTCCCCATTCCTTTGGAGAAGTTATTCACGGGCCCCAGCAGTGCCTTTGTTGTTCAACCACAAAACGGCAGGTGCACGACTGATGGCGTGCTCCTAGGCACCACCCAACTGTCTCCTGTCAACATCTGTACCTTCAGAGGGGATGTCACCCATATCACAGGTAGTCATAACTACACAATGAATTTGGCTTCTCAAAATTGGAACAATTATGACCCAACAGAAGAAATCCCAGCCCCTCTAGGAACTCCAGATTTTGTGGGGAAGATCCAAGGCATGCTCACCCAAACCACAAGGACAGATGGCTCAACACGCGGCCACAAAGCTACAGTGTACACCGGGAGCGCCGACTTCGCTCCAAAACTGGGTAGAGTTCAATTTGAAACTGACACAAACCATGACTTTGAAGCTAACCAAAACACAAAGTTCACCCCAGTCGGTGTCATCCAAGATGGTAGCACCACCCACCGAAATGAACCCCAACAGTGGGTGCTCCCAAGTTATTCAGGCAGAAACACTCATAATGTACATCTGGCCCCCGCTGTGGCCCCCACTTTTCCAGGTGAGCAGCTTCTCTTCTTCCGATCCACCATGCCCGGATGCAGCGGGTACCCCAACATGGATCTGGATTGTCTCCTCCCCCAGGAATGGGTGCAGCACTTCTACCAAGAGGCAGCCCCAGCACAATCTGATGTGGCCCTGCTGAGATTTGTGAACCCAGACACAGGTAGGGTTTTGTTTGAGTGTAAACTCCACAAATCAGGTTATGTTACTGTGGCTCACACTGGCCAACATGATTTGGTTATCCCCCCCAATGGCTATTTTAGGTTTGATTCCTGGGTCAACCAGTTCTACACGCTTGCCCCCATGGGAAATGGAGCGGGGCGTAGACGTGTAGTATAATGGCTG |
| MG786781.1 THA/2015/GII.4 Sydney(P31) | ATGAAGATCCATTTGAAACAATGATACCACACTCCCAAAGACCCATACAATTGATGTCCTTGCTGGGCGAGGCTGCACTCCACGGCCCGGCATTCTATAGCAAAATTAGCAAATTAGTCATTGCAGAGTTGAAGGAAGGTGGCATGGACTTTTACGTACCCAGACAAGAGCCAATGTTCAGATGGATGAGATTCTCAGATCTGAGCACGTGGGAGGGCGATCGCAATCTGGCTCCCAGTTTTGTGAATGAAGATGGCGTCGAGTGACGCCAACCCATCTGATGGGTCCGCAGCCAACCTCGTCCCAGAGGTCAACAATGAGGTTATGGCTCTGGAGCCCGTTGTTGGTGCCGCCATTGCGGCACCTGTAGCGGGCCAACAAAATGTAATTGACCCCTGGATTAGAAATAATTTTGTACAAGCCCCTGGTGGAGAGTTTACAGTATCCCCTAGAAACGCTCCAGGTGAAATACTATGGAGCGCGCCCCTGGGCCCTGATCTAAATCCCTACCTATCCCATTTGGCCAGAATGTACAATGGTTATGCAGGTGGTTTTGAAGTGCAGGTAATTCTCGCGGGGAACGCGTTCACCGCCGGGAAGGTCATATTTGCAGCAGTCCCACCAAATTTTCCAACTGAGGGCTTGAGCCCCAGCCAGGTCACTATGTTCCCCCATATAGTAGTAGATGTTAGGCAATTAGAACCTGTGTTGATTCCCTTACCCGATGTTAGAAATAATTTCTATCATTACAATCAATCAAATGATCCCACCATTAAGTTGATAGCAATGTTGTACACACCACTTAGGGCTAATAATGCTGGGGATGATGTCTTCACAGTTTCTTGCCGAGTTCTCACGAGACCATCCCCCGATTTTGATTTCATATTTCTAGTGCCACCCACAGTTGAATCAAGAACTAAACCATTCTCTGTCCCAGTTTTAACTGTTGAGGAGATGACCAATTCAAGATTCCCCATCCCTTTGGAAAAGTTGTTCACGGGTCCCAGCAGTGCCTTTGTTGTCCAACCACAAAACGGTAGGTGCACGACTGACGGCGTGCTCCTAGGCACCACCCAACTGTCTCCTGTCAACATCTGCACCTTCAGAGGAGATGTCACCCATATCACAGGTAGTCGCAACTACACAATGAATTTGGCTTCTCAAAATTGGAGCAATTATGACCCAACAGAAGAAATCCCAGCCCCTCTAGGAACTCCAGATTTTGTGGGGAAGATTCAAGGCATGCTCACCCAAACCACAAGGACAGATGGCTCAACACGCGGCCACAAAGCCACAGTGTACACTGGGAGCGCCGACTTTGCTCCAAAACTGGGTAGAGTTCAATTTGAAACTGACACAGACCATGATTTTGAAGCTAACCAAGACACAAAGTTCACCCCAGTTGGTGTCATCCAAGATGGTAGCACCACCCACCGAAATGAACCCCAACAGTGGGTGCTCCCAAGTTACTCAGGCAGAAATACTCCTAATGTGCATCTGGCCCCCGCTGTAGCCCCCACTTTTCCGGGTGAGCAACTTCTCTTCTTCAGATCCACCATGCCCGGATGCAGCGGGTACCCCAACATGGATTTGGACTGTCTGCTCCCCCAGGAATGGGTGCAGTACTTCTACCAAGAGGCAGCCCCAGCACAATCTGATGTGGCTCTGCTAAGATTTGTGAATCCAGACACAGGTAGGGTTTTGTTTGAGTGTAAGCTTCATAAATCAGGCTATGTCACAGTGGCTCACACTGGCCAGCATGATTTGGTTATCCCCCCCAATGGTTATTTTAGGTTTGATTCCTGGGTCAACCAGTTTTACACGCTTGCCCCCATGGGAAATGGAACGGGGCGTAGACGTGCACTATAATGGCTG |
| MG763422.1 CHN/2017/GII.4 Sydney(P16) | ATGAAGACCCCAATGAGACAATGATACCCCATTCTCAAAGACCCGTACAGCTCATGGCACTGCTTGGTGAAGCCTCTCTTCACGGACCCTCTTTCTACAGTAGAATCAGTAAATTGGTCATAACTGAGCTCAAAGAAGGTGGGATGGACTTTTACGTGCCAAGGCAGGAACCCATGTTCAGGTGGATGAGGTTTTCTGACTTGAGCACGTGGGAGGGCGATCGCAATCTGGCTCCCAATTTTGTGAATGAAGATGGCGTCGAGTGACGCCAACCCATCTGATGGGTCCGCAGCCAACCTCGTACCAGAGGTCAACAATGAGGTTATGGCTTTGGAGCCCGTTGTTGGTGCCGCTATTGCGGCACCTGTAGCGGGCCAACAAAATGTAATTGACCCCTGGATTAGAAATAATTTTGTACAAGCCCCTGGTGGGGAGTTTACAGTATCCCCTAGAAACGCTCCAGGTGAAATACTATGGAGC |
| MN996297.1 CHN/2018/GII.4 Sydney(P16) | ATGAAGACCCCAATGAGACAATGATACCCCATTCTCAAAGACCCATACAGCTCATGGCACTGCTTGGTGAAGCCTCTCTTCACGGACCCTCTTTCTACAGTAGAATCAGTAAACTGGTCATAACTGAACTCAAAGAAGGTGGGATGGACTTTTACGTGCCAAGGCAGGAACCCATGTTCAGGTGGATGAGGTTTTCTGACTTGAGCACGTGGGAGGGCGATCGCAATCTGGCTCCCAATTTTGTGAATGAAGATGGCGTCGAGTGACGCCAACCCATCTGATGGGTCCGCAGCCAACCTCGTACCAGAGGTCAACAATGAGGTTATGGCTTTGGAGCCCGTTGTTGGTGCCGCTATTGCGGCACCTGTAGCGGGCCAACAAAATGTAATTGACCCCTGGATTAGAAATAATTTTGTACAAGCCCCTGGTGGGGAATTTACAGTGTCCCCTAGAAACGCTCCAGGTGAAATACTATGGAGCGCGCCCCTAGGCCCTGACCTAAATCCCTACCTATCCCATTTAGCCAGAATGTACAATGGTTATGCAGGTGGTTTTGAAGTGCAGGTAATTCTCGCGGGGAACGCGTTCACTGCCGGGAAGATTATATTTGCAGCAGTCCCACCAAATTTTCCAACTGAAGGCTTAAGTCCTAGCCAGGTCACTATGTTCCCCCACATAATAGTAGATGTTAGACAGCTAGAACCCGTGCTGATCCCCTTACCCGATGTTAGGAATAATTTCTATCATTACAATCAGTCAAATGACTCCACTATTAAGTTGATAGCAATGTTGTATACACCACTTAGGGCTAATAATGCTGGAGATGATGTTTTCACAGTTTCGTGCCGAGTTCTTACGAGACCATCCCCCGATTTTGATTTCATATTTTTGGTGCCACCCACAGTTGAGTCAAGAACTAAACCATTCTCTGTCCCAATTTTAACTGTTGAGGAGATGACCAATTCAAGATTCCCCATCCCTTTGGAAAAGTTGTTCACAGGCCCCAGTAGTGCCTTTGTTGTTCAACCACAAAACGGCAGGTGCACAACTGATGGCGTGCTCCTAGGCACCACCCAACTTTCTCCTGTCAACATCTGCACCTTCAGAGGGGATGTCACCCACATCACAGGTAGTCGCAACTACACAATGAATTTGGCTTCTCAAAATTGGAACAACTATGACCCAACAGAAGAGATCCCAGCCCCTCTAGGAACTCCAGATTTTGTGGGGAAGATTCAAGGCATGCTCACCCAAACCACAAGGACAGATGGTTCAACACGCGGCCACAAAGCTACAGTTTACACTGGGAGCGCCGACTTTGCTCCAAAATTGGGTAGAGTTCAATTTGAAACTGACACAGACCATGATTTTGAAGCTAATCAAAACACAAAGTTCACCCCAGTCGGTGTCATCCAAGATGGTAGCACCACCCACCGAAACGAACCCCAACAGTGGGTGCTCCCAAGTTACTCAGGCAGAAACACTCACAATGTACATTTGGCCCCCGCTGTAGCCCCCACCTTTCCGGGTGAGCAACTTCTCTTCTTCAGATCCACCATGCCCGGATGCAGCGGGTACCCCAACATGGATTTGGACTGTCTGCTCCCCCAGGAATGGGTGCAGTACTTCTACCAAGAGGCAGCCCCAGCACAATCTGATGTGGCTCTGCTAAGATTTGTGAATCCAGACACAGGTAGGGTTTTGTTTGAGTGCAAGCTCCACAAATCAGGCTATGTTACAGTGGCTCACACTGGCCAACATGATTTGGTTATCCCCCCCAACGGTTACTTTAGGTTTGATTCCTGGGTCAACCAGTTCTACACACTTGCCCCCATGGGAAATGGAACGGGGCGTAGACGTGTAGTGTAATGGCTG |
| MH569710.1 CHN/2018/GII.4 Sydney(P16) | ATGAAGACCCCAATGAGACAATGATACCCCATTCCCAAAGACCTATACAGCTCATGGCACTGCTTGGTGAAGCCTCTCTTCACGGACCCTCTTTTTACAGTAAAATCAGTAAGTTGGTCATAACTGAACTCAAAGAAGGTGGGATGGACTTTTACGTGCCAAGGCAGGAACCCATGTTCAGGTGGATGAGGTTTTCTGACTTGAGCACGTGGGAGGGCGATCGCAATCTGGCTCCCAATTTTGTGAATGAAGATGGCGTCGAGTGACGCCAACCCATCTGATGGGTCCGCAGCCAACCTCGTACCAGAGGTCAACAATGAGGTTATGGCTTTGGAGCCCGTTGTTGGTGCCGCTATCGCGGCACCTGTAGCGGGCCAACAAAATGTAATTGACCCCTGGATTAGAAATAATTTTGTACAAGCCCCTGGTGGAGAGTTTACAGTGTCCCCTAGAAACGCTCCAGGTGAAATACTATGGAGCGCGCCCCTAGGCCCTGACCTAAATCCCTACCT |
| MW661264.1 CAN/2019/GII.4 Sydney(P16) | ATGAAGATCCCAATGAGACAATGATACCCCATTCTCAAAGACCCATACAGCTCATGGCACTGCTTGGTGAAGCCTCTCTTCACGGACCCTCTTTCTACAGTAGAATTAGTAAATTGGTCATAACTGAACTCAAAGAAGGTGGGATGGACTTTTACGTGCCAAGGCAGGAACCCATGTTCAGGTGGATGAGGTTTTCTGACTTGAGCACGTGGGAGGGCGATCGCAATCTGGCTCCCAATTTTGTGAATGAAGATGGCGTCGAGTGACGCCAACCCATCTGATGGGTCCGCAGCCAACCTCGTACCAGAGGTCAACAATGAGGTTATGGCTTTGGAGCCCGTTGTTGGTGCCGCTATCGCGGCACCTGTAGCGGGCCAACAAAATGTAATTGACCCCTGGATTAGAAATAATTTTGTACAAGCCCCTGGTGGGGAGTTTACAGTGTCCCCTAGAAACGCTCCAGGTGAAATACTATGGAGCGCGCCCCTAGGCCCTGACCTAAATCCCTACCTATCCCATTTGGCCAGAATGTACAATGGTTATGCAGGTGGTTTTGAAGTGCAGGTAATTCTCGCGGGGAACGCGTTCACCGCCGGGAAGATTATATTTGCAGCAGTCCCACCAAATTTTCCAACTGAAGGCTTAAGTCCTAGCCAGGTCACTATGTTCCCCCATATAATAGTAGATGTTAGACAACTAGAACCTGTGCTGATTCCCTTACCTGATGTTAGGAATAATTTTTATCATTACAATCAGTCAAATGATTCCACTATCAAGTTGATAGCAATGTTGTATACACCACTTAGGGCTAATAATGCAGGAGATGATGTTTTCACAGTTTCGTGCCGAGTTCTCACGAGACCATCCCCCGATTTTGATTTCATATTTTTAGTGCCACCCACAGTTGAGTCAAGAACTAAACCATTCTCTGTCCCAGTTTTAACTGTTGAGGAGATGACCAATTCAAGATTCCCCATCCCTCTGGAAAAGTTGTTCACAGGCCCCAGCAGTGCCTTTGTTGTTCAACCACAAAACGGCAGGTGCACAACTGATGGCGTGCTCCTAGGCACCACCCAACTTTCTCCTGTCAACATCTGCACCTTCAGAGGGGATGTCACCCACATCACAGGCAGTCGCAACTACACAATGAATTTGGCTTCTCAAAATTGGAACAACTATGACCCAACAGAAGAAATCCCAGCCCCTCTAGGAACTCCAGATTTTGCAGGGAAGATTCAAGGCATGCTCACCCAAACCACAAGGACAGACGGTTCAACACGCGGCCACAAAGCTACAGTGTACACTGGGAGCGCCGACTTTGCTCCAAAACTGGGTAGAGTTCAATTTGAAACTGACACAGACCATGATTTTGAAGCTAATCAAAACACAAAGTTCACCCCAGTCGGTGTCATCCAAGATGGCAGCACCACCCACCGAAACGAACCCCAACAGTGGGTGCTCCCAAGTTACTCAGGCAGAAATACTCACAATGTACATTTGGCCCCCGCTGTAGCCCCCACCTTTCCGGGTGAGCAGCTCCTCTTTTTCAGATCCACCATGCCCGGATGCAGCGGGTACCCCAACATGGATTTGGACTGTCTGCTCCCCCAGGAATGGGTGCAGTACTTCTACCAAGAGGCAGCCCCAGCACAATCTGATGTGGCTCTGCTAAGATTTGTGAATCCAGACACAGGTAGGGTTTTGTTTGAGTGCAAGCTTCACAAATCAGGCTATGTTACAGTGGCCCACACTGGCCAACATGATTTGGTTATCCCCCCCAATGGTTACTTTAGGTTTGATTCCTGGGTCAACCAGTTCTACACGCTTGCCCCCATGGGAAATGGAACGGGGCGTAGACGTGCAGTATAATGGCTG |
| MT735394.1 NZL/2017/GII.4 Hong Kong(P31) | ATGAGGATCCATCTGAAACAATGATACCACACTCCCAAAGACCCATACAATTGATGTCACTGCTGGGCGAGGCTGCACTCCATGGCCCGGCATTCTATAGCAAAATTAGCAAGTTAGTTATTGCAGAGTTGAAGGAAGGTGGCATGGATTTTTACGTGCCCAGACAAGAGCCAATGTTCAGATGGATGAGATTCTCAGATCTGAGCACGTGGGAGGGCGATCGCAATCTGGCTCCCAGTTTTGTGAATGAAGATGGCGTCGAATGACGCCAACCCATCTGATGGGTCCACAGCCAACCTCGTCCCAGAGGTCAACAATGAGGTTATGGCTTTGGAGCCCGTTGTAGGCGCCGCTATTGCGGCACCTGTGGCGGGCCAACAAAATTTAATTGACCCCTGGATTAGAAATAATTTTGTACAAGCCCCTGGTGGAGAGTTCACAGTGTCCCCCAGAAACGCTCCAGGTGAAATACTATGGAGCGCGCCTTTGGGCCCCGATTTGAACCCTTATCTTTCTCATTTGGCCAGAATGTACAATGGTTATGCAGGTGGCTTTGAGGTGCAGGTAATCCTTGCGGGGAACGCGTTCACCGCCGGGAAAATCATATTTGCAGCAGTCCCACCAAATTTCCCAACTGAAGGCTTGAGCCCCAGCCAGGTTACCATGTTTCCCCATGTAATAGTAGATGTTAGGCAATTAGAACCTGTGTTGATCCCTTTACCTGATGTTAGGAATAATTTCTATCATTATAATCAATCAAATGATTCTACCATTAAATTGATAGCAATGCTGTATACACCACTTAGGGCAAATAATGCTGGGGATGATGTCTTCACGGTCTCTTGTCGGGTCCTTACGAGGCCATCCCCTGATTTTGATTTCATATTTCTGGTACCACCAACAGTTGAGTCAAGAACTAAACCTTTTACTGTCCCAATCCTAACCGTTGAGGAAATGACCAATTCAAGATTCCCCATTCCCTTGGAGAAGCTGTATACGGGTCCCAGCACTGCTTTTGTTGTTCAGCCACAAAATGGTAGATGCACGACTGATGGCGTGCTCTTGGGCACTACCCAGCTGTCTGCTGTCAACATCTGTACCTTTAGAGGAGATCTCACCCACATTGCAGGCACTCGCCAATTCACAATGCGTCTGGCCTCTCCGAACTGGAACAACTATGATCCAACAGAAGAAATCCCAGCCCCCCTGGGTACCCCAGATTTCGTGGGGAAGATTCAAGGCATGCTCACCCAAACCACAAAAGGAGATGGCTCGACCCGCGGCCACAAAGCCATAGTGTCCACTGGGGCTGCCGACTTTGCCCCAAAATCAGGCAACATTCGATTCGGCACTGACACAGAAGATGATCTCCAATCTGGCACAAACACGAAATTCACCCCAATTGGCGTCGTCCAAGATGGTGAAAACCCCCACTTTAGTGAACCCCAACAATGGGTGCTCCCAAGTTACACAGGTAGAACTGGACATAATATGCATTTGGCCCCCGCTGTTGCCCCCACTTACCCGGGTGAGCAACTCCTTTTCTTCAGGTCCACCATGCCCGGATGCAGCGGGTACCCCAACTTGGATATAGACTGCTTGCTCCCCCAGGAATGGGTGCAGCACTTCTACCAAGAAGCAGCTCCAGCACAATCTGATGTGGCTTTGCTAAGATTTGTGAATCCAGACACAGGTAGGGTTTTGTTTGAGTGCAAGCTTCATAAGTCAGGATATGTTACAGTAGCTCATACTGGTCCTCATGATCTAGTTATCCCCCCCAATGGTTATTTTAGATTTGATTCCTGGGTCAACCAGTTTTACACACTTGCCCCCATGGGAAATGGAACGGGGCGCAGACGTGTATTATAATGGCTG |
| U07611.2 USA/1971/GII.1(P1) | ATGAGGATCCATCTGAAACAATGATACCACATTCCCAAAGGCCCATACAGTTGATGTCTCTGCTAGGTGAAGCTGCATTGCACGGTCCAGCATTCTACAGCAAAATCAGTAAACTAGTCATTGCAGAGTTGAAGGAAGGTGGCATGGACTTTTACGTGCCCAGGCAAGAGCCGATGTTCAGATGGATGAGATTCTCAGACCTGAGCACGTGGGAGGGCGATCGCAATCTGGCTCCCAGTTTTGTGAATGAAGATGGCGTCGAATGACGCCGCCCCATCTAATGATGGTGCAGCCGGTCTCGTACCAGAGGTCAACAACGAGACGATGGCCCTCGAACCGGTGGCTGGGGCTTCTATAGCCGCCCCTCTAACCGGTCAAAATAATGTGATAGACCCCTGGATTAGAATGAACTTTGTCCAAGCCCCAAATGGAGAATTCACAGTGTCTCCCCGCAATTCTCCTGGTGAAATCTTGCTAAATTTGGAATTAGGCCCTGAATTAAATCCATTCTTAGCACACCTTTCAAGAATGTATAATGGTTATGCCGGCGGGGTTGAAGTGCAGGTACTACTCGCTGGGAACGCGTTCACAGCGGGAAAACTGGTGTTTGCAGCAATCCCCCCGCACTTCCCTCTTGAGAATCTGAGTCCTGGACAAATTACAATGTTCCCTCATGTGATTATTGATGTTAGAACATTAGAACCTGTGCTTTTGCCCCTTCCAGATGTTAGAAATAATTTCTTTCATTACAATCAGCAGCCCGAGCCCCGTATGAGACTTGTAGCTATGTTGTATACTCCTCTTAGATCTAATGGTTCTGGTGATGATGTGTTCACAGTTTCTTGCAGGGTTCTCACCCGCCCTTCTCCAGATTTTGATTTTAATTATTTGGTTCCCCCAACTGTGGAGTCTAAAACTAAACCATTCACCCTGCCAATCCTAACTATTGGAGAATTGTCAAATTCTAGATTCCCAGTTCCAATAGATGAATTGTACACCAGCCCCAATGAAGGAGTGATCGTGCAGCCCCAAAATGGCAGATCAACACTTGATGGTGAATTGTTGGGCACCACGCAACTCGTGCCCTCAAACATCTGTGCGCTACGAGGGCGCATTAACGCCCAGGTGCCAGATGATCACCATCAATGGAACCTACAGGTAACAAACACAAATGGGACTCCTTTCGACCCCACCGAAGACGTCCCTGCACCACTGGGCACACCGGATTTCCTGGCGAATATCTATGGAGTCACCAGCCAGAGAAACCCCAACAACACTTGCCGTGCCCATGATGGGGTTTTGGCAACTTGGAGCCCCAAATTTACACCCAAGTTAGGATCTGTGATTTTGGGCACTTGGGAAGAAAGTGATCTTGATCTCAATCAGCCCACAAGGTTCACACCTGTTGGTCTGTTTAACACTGACCACTTTGATCAGTGGGCCTTGCCTAGTTATTCTGGAAGATTAACCCTAAACATGAATTTGGCACCCTCTGTTTCCCCCCTCTTTCCAGGTGAACAGCTACTTTTCTTCAGGTCCCATATACCACTCAAAGGAGGTACCTCTGATGGTGCCATTGATTGTCTACTCCCCCAGGAATGGATTCAGCATTTTTATCAGGAGTCAGCCCCATCGCCCACGGACGTGGCTCTAATTAGATACACCAATCCTGACACAGGCCGCGTTTTGTTTGAAGCTAAACTGCACAGGCAAGGATTCATCACAGTGGCAAACTCTGGTTCTAGGCCTATTGTTGTCCCTCCGAATGGCTATTTTAGGTTTGATTCTTGGGTTAATCAATTCTATTCTCTCGCCCCCATGGGAACTGGGAACGGGCGCAGAAGAGTGCAGTAATGGCTG |
| LC209457.1 JPA/2015/GII.2(P2) | ATGAAGACCCCTTTGAGTCAATGATCCCACATTCCCAGAGACCCATACAACTCATGTCACTCTTGGGCGAAGCAGCACTGCATGAACCATCATTTTACAGCAAGATCAGCAAGCTTGTGATATCTGAATTGAAAGAAGGTGGAATGGATTTTTACGTGCCCAGACAAGAACCTATGTTTAGGTGGATGAGATTCTCAGATTTGAGCACGTGGGAGGGCGATCGCAATCTTGCTCCCAGTCTTGTGAATGAAGATGGCGTCGAATGACGCCGCTCCATCTACTGATGGTGCAGCCGGCCTCGTGCCAGAAAGTAATAATGAGGTCATGGCTCTTGAACCCGTGGCTGGTGCCGCCTTGGCAGCCCCAGTCACCGGTCAAACAAATATTATAGACCCTTGGATTAGAGCAAATTTTGTCCAGGCCCCCAATGGTGAATTTACAGTCTCTCCCCGTAATGCACCTGGTGAAGTGCTATTGAATCTAGAGTTGGGTCCAGAATTAAATCCTTATCTGGCACATTTAGCAAGAATGTACAACGGGTATGCCGGTGGGATGGAGGTGCAGGTCATGTTGGCTGGGAACGCGTTCACAGCCGGCAAGTTGGTCTTCGCCGCTGTACCACCTCATTTCCCGGTTGAAAACCTTAGTCCACAGCAAATTACCATGTTCCCTCATGTGATTATAGATGTAAGGACCTTGGAACCTGTTTTATTACCACTCCCTGACGTTAGAAATAATTTCTTCCATTATAACCAGAAAGATGATCCTAAGATGAGAATTGTGGCTATGCTTTACACCCCCCTTAGGTCTAATGGTTCAGGTGATGATGTATTCACAGTCTCCTGCAGGGTGTTGACTAGACCCTCCCCTGATTTTGATTTCACATACCTGGTGCCACCAACAGTAGAATCTAAAACAAAACCATTCACCCTCCCAATTCTCACACTTGGGGAACTTTCCAATTCTAGATTTCCAGTGTCCATAGATCAGATGTACACCAGCCCCAATGAAGTCATATCAGTGCAGTGTCAAAATGGTAGGTGCACACTGGATGGGGAGCTCCAAGGAACAACACAACTCCAAGTCAGTGGCATTTGTGCTTTCAAGGGAGAAGTAACCGCCCACTTGCATGACAACGATCACCTATATAACATCACCATCACAAACTTGAATGGGTCCCCTTTTGATCCCTCCGAGGATATCCCTGCCCCCTTGGGCGTACCTGACTTTCAGGGAAGGGTCTTTGGTATCATCTCCCAAAGAGACAAACACAATTCCCCTGGACATAATGAACCAGCAAATAGGGGACATGACGCTGTGGTCCCTACTTACACAGCACAGTACACTCCAAAATTGGGTCAGATTCAAATTGGCACATGGCAGACCGACGATCTCACAGTCAGCCAACCAGTGAAATTCACCCCAGTTGGGCTCAATGATACTGAACATTTTAATCAATGGGTGGTTCCTAGATATGCTGGTGCTCTAAATCTTAATACAAATCTTGCCCCTTCTGTTGCTCCAGTATTTCCAGGGGAGCGCCTACTCTTTTTCAGATCATATATCCCCCTCAAGGGTGGTTATGGAAATCCAGCCATTGATTGTCTACTACCACAAGAGTGGGTGCAACATTTCTACCAGGAGGCAGCCCCTTCAATGAGTGAGGTGGCCCTTGTCAGATACATCAACCCGGATACTGGTCGAGCACTGTTCGAGGCCAAGCTCCACAGAGCTGGTTTCATGACAGTCTCAAGCAACACTAGTGCCCCGGTGGTTGTGCCTGCCAACGGGTACTTTAGATTTGATTCTTGGGTGAACCAATTCTATTCTCTCGCCCCCATGGGAACTGGAAATGGGCGTAGAAGGGTTCAATAATGGCTG |
| MK764040.1 US/2016/GII.2(P2) | ATGAAGACCCCTTTGAGTCAATGATTCCACACTCCCAGAGACCCATACAACTCATGTCACTCTTGGGTGAGGCGGCACTGCATGAACCGTCATTTTACAGCAAGATCAGCAAGCTTGTGATATCTGAATTGAAAGAAGGTGGAATGGATTTTTACGTGCCCAGGCAAGAACCCATGTTTAGGTGGATGAGATTCTCGGATTTGAGCACGTGGGAGGGCGATCGCAATCTTGCTCCCAGTCTTGTGAATGAAGATGGCGTCGAATGACGCCGCTCCATCTACTGATGGTGCAGCCGGCCTCGTGCCAGAAAGTAATACTGAGGTCATGGCTCTTGAACCCGTGGCTGGTGCCGCTTTGGCAGCCCCAGTCACTGGTCAAACAAATATTATAGACCCTTGGATTAGAGCAAATTTTGTCCAGGCCCCCAATGGTGAATTTACAGTCTCCCCCCGTAATGCCCCTGGTGAAGTGCTACTAAATCTGGAGTTGGGTCCAGAATTAAATCCTTATCTGGCACACTTAGCAAGAATGTACAACGGGTATGCCGGTGGGATGGAGGTGCAGGTCATGTTGGCTGGGAACGCGTTCACAGCCGGCAAGTTGGTCTTCGCCGCCGTGCCACCTCACTTCCCGGTTGAAAACCTTAGTCCACAACAAATTACCATGTTCCCTCATGTGATTATAGATGTGAGGACTTTGGAACCTGTCTTATTACCACTCCCTGATGTTAGAAATAATTTCTTTCATTACAATCAGAAGGATGATCCCAAGATGAGAATTGTGGCTATGCTTTATACCCCCCTTAGATCAAATGGCTCAGGTGATGATGTGTTCACAGTCTCCTGTAGGGTGTTAACTAGGCCCTCCCCTGACTTTGACTTCACATATCTGGTGCCACCAACAGTGGAGTCTAAAACAAAGCCATTCACCCTCCCAATCCTCACACTTGGGGAACTTTCCAATTCCAGATTCCCAGTGTCTATAGACCAGATGTACACCAGCCCTAATGAAGTTATATCAGTGCAGTGTCAAAATGGTAGGTGCACATTGGACGGGGAGCTCCAAGGGACAACACAACTCCAGGTCAGTGGCATTTGTGCTTTCAAGGGAGAGGTAACCGCCCACTTGCATGACAATGATCACCTATACAATATCACCATCACAAACTTGAATGGATCCCCTTTTGACCCCTCTGAGGACATCCCTGCCCCTCTGGGCGTACCTGACTTTCAGGGAAGGGTCTTTGGTATTATCTCCCAAAGGGACAAACACAATAGCCCTGGACATAATGAACCAGCAAACAGGGGACACGACGCTGTGGTCCCTACTTACACAGCACAGTATACTCCAAAATTGGGTCAGATTCAAATTGGCACATGGCAGACTGACGATCTCACTGTCAACCAACCAGTAAAATTCACCCCAGTTGGACTCAATGATACTGAACATTTTAACCAATGGGTGGTCCCCAGATATGCTGGTGCCCTAAACCTCAACACAAACCTTGCCCCTTCTGTTGCCCCAGTATTTCCAGGGGAGCGCCTGCTCTTTTTCAGATCATACATCCCCCTCAAGGGTGGCTATGGAAATCCAGCCATTGATTGCCTACTACCACAAGAGTGGGTGCAACACTTCTACCAGGAAGCAGCCCCTTCTATGAGTGAGGTGGCCCTTGTCAGGTACATCAACCCGGACACTGGTCGGGCACTGTTTGAGGCCAAGCTCCACAGAGCTGGTTTCATGACGGTCTCAAGTAACACCAGTGCCCCGGTGGTTGTACCTGTCAATGGATACTTTAGGTTTGACTCTTGGGTGAACCAATTTTATTCTCTCGCCCCCATGGGAACTGGAAATGGGCGTAGAAGGGTTCAATAATGGCTG |
| NC039476.1 CHN/2016/GII.2(P16) | ATGAAGACCCCAATGAGACAATGATACCCCATTCTCAAAGACCCATACAGCTCATGGCACTGCTTGGTGAAGCCTCTCTTCACGGACCCTCTTTCTACAGTAAAATCAGTAAATTGGTCATAACTGAACTCAAAGAAGGTGGGATGGACTTTTACGTGCCAAGGCAGGAACCCATGTTCAGGTGGATGAGGTTTTCTGACTTGAGCACGTGGGAGGGCGATCGCAATCTGGCTCCCAATTTTGTGAATGAAGATGGCGTCGAATGACGCCGCTCCATCTACTGATGGTGCAGCCGGCCTCGTGCCAGAAAGTAACAATGAGGTCATGGCTCTTGAACCCGTGGCTGGTGCCGCCTTGGCAGCCCCGGTCACCGGTCAAACAAATATTATAGACCCTTGGATTAGAGCAAATTTTGTCCAGGCCCCCAATGGTGAATTTACAGTCTCTCCCCGAAATGCCCCTGGTGAAGTGCTACTGAATCTAGAGTTGGGTCCAGAATTAAATCCTTATCTGGCACATTTAGCAAGAATGTACAATGGGTATGCCGGTGGGATGGAGGTGCAGGTCATGTTGGCTGGGAACGCGTTCACAGCCGGCAAGTTGGTCTTCGCCGCCGTGCCACCCCACTTCCCGGTTGAAAACCTTAGCCCACAGCAAATCACCATGTTCCCTCATGTGATCATAGATGTGAGAACCTTGGAACCTGTTTTATTACCACTCCCTGATGTTAGGAATAACTTCTTCCATTATAACCAGAAAGATGATCCCAAGATGAGAATTGTGGCTATGCTTTATACCCCCCTCAGGTCTAATGGCTCAGGTGATGATGTGTTTACAGTCTCCTGTAGAGTGTTGACTAGACCTTCCCCTGACTTTGACTTCACATACCTGGTGCCACCAACAGTGGAGTCTAAAACAAAGCCATTCACCCTCCCAATCCTCACACTTGGGGAACTTTCCAATTCTAGGTTCCCAGTGTCCATAGACCAGATGTACACCAGCCCTAATGAAATTATATCAGTGCAGTGTCAAAATGGTAGGTGCACACTGGACGGGGAGCTCCAAGGGACAACACAACTCCAAGTCAGTGGCATTTGTGCTTTCAAAGGTGAAGTGACCGCCCACTTGCATGACAATGATCACCTATATAATGTCACCATCACAAACTTGAATGGGTCCCCTTTTGATCCCTCCGAGGATATCCCTGCCCCTCTGGGTGTGCCTGACTTCCAGGGTAGGGTTTTTGGTATCATCTCCCAAAGAGATAAACACAATAGTCCTGGGCATAATGAACCAGCAAACAGGGGACACGACGCTGTGGTCCCTACTTACACAGCACAGTACACTCCAAAACTTGGACAAATTCAAATTGGCACATGGCAAACTGACGACCTTACAGTCAACCAACCAGTCAAATTCACCCCAGTTGGACTCAATGACACTGAACACTTTAACCAATGGGTGGTCCCTAGGTATGCTGGTGCCCTAAACCTCAATACAAACCTTGCCCCTTCTGTTGCTCCAGTATTTCCAGGAGAGCGCCTGCTCTTCTTCAGATCATACATTCCCCTCAAGGGCGGTTATGGAAACCCAGCCATTGATTGCCTACTACCACAAGAGTGGGTGCAACACTTCTATCAGGAAGCAGCCCCTTCAATGAGTGAGGTGGCCCTCGTCAGATACATCAACCCGGACACTGGTCGGGCACTGTTTGAGGCCAAGCTCCACAGAGCTGGTTTCATGACAGTCTCGAGCAACACCAGTGCCCCGGTGGTTGTGCCTGCCAACGGGTACTTCAGATTTGATTCTTGGGTGAACCAATTTTATTCTCTCGCCCCCATGGGAACTGGGAATGGGCGTAGAAGGGTTCAATAATGGCTG |
| MK073886.1 USA/2016/GII.3(P12) | ATGAAGATCCATCTGAAACAATGATACCACACTCCCAGAGGCCCATACAATTGATGTCCTTGCTGGGAGAGGCAGCACTCCACGGCCCAGCATTCTACAGCAAAATCAGTAAACTGGTCATTGCAGAGTTGAAGGAGGGTGGCATGGATTTTTACGTGCCAAGACAAGAGCCAATGTTCAGATGGATGAGATTCTCGGATCTGAGCACGTGGGAGGGCGATCGCAATCTGGCTCCCAGTTTTGTGAATGAAGATGGCGTCGAATGACGCCACTCCATCTAATGATGGTGCCGCCGGCCTCGTCCCAGAGATCAACAATGAGGCAATGGCGCTAGAACCAGTGGCGGGTGCAGCGATAGCAGCACCCCTCACCGGCCAGCAAAATATAATTGATCCCTGGATTATGAATAATTTTGTGCAAGCACCTGGTGGTGAGTTCACAGTGTCTCCTAGGAATGCCCCTGGTGAAGTGCTCCTCAATTTGGAATTGGGCCCAGAGATAAACCCCTATCTGGCCCATCTTGCTAGAATGTATAATGGTTATGCAGGTGGGTTTGAAGTGCAGGTGGTCCTGGCTGGAAATGCGTTTACAGCAGGAAAGATAATCTTTGCAGCTATACCCCCTAACTTCCCAATTGACAATCTAAGTGCAGCACAGATCACAATGTGTCCACACGTGATTGTGGATGTCAGGCAGTTGGAACCGGTCAACCTCCCGATGCCTGACGTTCGTAACAACTTCTTCCACTACAACCAAGGGTCTGATTCGAGATTGCGCTTGGTTGCAATGCTGTATACACCTCTTAGGGCAAATAACTCTGGGGATGATGTTTTCACTGTGTCTTGTAGAGTGCTGACTAGACCTAGTCCTGAATTTTCATTTAACTTCCTTGTGCCCCCCACTGTGGAATCAAAGACAAAACCCTTTACCCTCCCAATTCTGACTATCTCTGAAATGTCTAATTCTAGGTTTCCAGTGCCGATTGACTCTCTGCACACCAGCCCAACTGAGAATATTGTTGTCCAGTGTCAAAATGGACGCGTCACTCTTGATGGTGAGTTGATGGGCACCACTCAGCTCTTACCTAGTCAAATCTGTGCTTTCAGGGGCGTGCTCACTAGATCAACAAGCAGGGCTAGTGACCAGGCCGACACAGCAACCCCTAGATTGTTTAATTATTATTGGCACATACAATTGGATAATCTAAATGGGACTCCTTATGATCCTGCAGAAGACATACCAGGCCCCCTAGGGACACCAGATTTCCGAGGCAAAGTCTTTGGCGTGGCCAGCCAAAGAAACCCCGACAGTACAACTAGAGCACATGAAGCAAAGGTGGACACAACAGCTGGTCGCTTCACCCCAAAACTAGGCTCATTAGAGATATCCACTGAATCTGGTGACTTTGACCAAAACCAACCAACAAGATTCACCCCAGTTGGCATTGGGGTTGACAACGAGGCAGACTTCCAACAATGGTCCTTACCAGACTACTCTGGCCAGTTCACTCACAACATGAACTTAGCCCCAGCTGTTGCTCCCAACTTCCCTGGTGAGCAGCTCCTTTTCTTCCGCTCACAGTTACCATCTTCTGGTGGGCGATCCAATGGGATTCTAGACTGCCTGGTCCCCCAAGAATGGGTCCAGCACTTCTACCAAGAATCAGCCCCCGCCCAAACCCAGGTGGCTCTGGTTAGATATGTCAACCCTGACACTGGTAGAGTGTTGTTTGAGGCCAAGCTGCACAAATTAGGTTTCATGACTGTAGCTAAGAATGGTGACTCTCCAATAACTGTCCCCCCAAATGGATACTTTAGGTTTGAATCTTGGGTGAACCCATTTTATACACTTGCCCCCATGGGAACTGGGAACGGGCGTAGAAGAGTTCAATAATGGCTG |
| LN854569.1 NLD/2014/GII.3(P21) | ATGAAGACCCCTTTGAAACAATGATACCACACTCCCAAAGACCCATACAACTGATGTCATTACTTGGTGAAGCTGCATTGCATGGTCCATCATTCTACAGTAAAGTCAGCAAATTGGTCATCTCAGAACTGAAAGAGGGTGGAATGGATTTTTACGTGCCCAGACAAGAACCAATGTTCAGGTGGATGAGATTCTCAGATTTGAGCACGTGGGAGGGCGATCGCAATCTGGCTCCCAGTTTTGTGAATGAAGATGGCGTCGAATGACGCCGCTCCATCTAATGATGGTGCCGCCGGCCTCGTCCCAGAGATCAACAATGAGGCAATGGCGCTAGAGCCAGTGGCGGGTGCAGCGATAGCAGCACCCCTCACTGGTCAGCAAAATATAATTGATCCCTGGATTATGAATAATTTTGTGCAAGCACCTGGTGGTGAGTTTACAGTGTCCCCCAGAAATTCCCCTGGTGAAGTTCTTCTTAATTTGGAATTGGGCCCAGAAATAAATCCCTATTTGGCCCATCTTGCTAGAATGTATAATGGTTATGCAGGTGGATTTGAAGTGCAGGTGGTCCTAGCTGGAAATGCGTTTACAGCAGGAAAGATAATCTTTGCAGCTATTCCCCCCAACTTTCCAATTGACAATTTAAGTGCAGCACAGATCACTATGTGCCCACATGTGATTGTAGATGTCAGACAGTTGGAACCAGTCAACCTCCCGATGCCTGACGTTCGCAATAACTTCTTTCATTATAATCAAGGGTCTGATTCGAGGTTACGCCTAATTGCAATGCTATATACACCTCTTAGAGCAAACAATTCTGGGGATGATGTTTTTACTGTGTCTTGTAGAGTGCTAACTAGACCTAGTCCTGACTTCTCATTCAATTTCCTTGTGCCACCTACTGTGGAGTCAAAGACAAAACCCTTCTCCCTCCCTATCCTGACTATTTCTGAAATGTCTAATTCTAGGTTCCCAGTACCAATTGATTCTCTGCACACCAGTCCTACTGAGAATATTGTTGTTCAGTGCCAAAATGGGCGCGTCACCCTTGATGGTGAGTTGATGGGCACCACCCAACTCTTGCCTAGCCAAATCTGTGCTTTCAGGGGAGTTCTCACCAGATCAACAAGCAGGGCCAGTGACCAGGCCGATACAGCAACCCCTAGATTGTTTAATTATTATTGGCATATACAATTGGATAATCTAAATGGAACCCCTTATGATCCTGCAGAAGACATACCAGGCCCCCTAGGGACACCAGATTTCCGTGGCAAAGTCTTTGGCGTGGCCAGCCAGAGAAATCTTGATACCACGACTAGGGCACATGAAGCAAAGATAGACACCACATCTGGCCGCTTCACCCCAAAGCTAGGCTCATTAGAAATATCCACTGAATCTAGTGATTTTGATCAAAGCCAACCAACAAGATTCACCCCAGTTGGCATTGGAGTTGACCATGAGGCAGACTTTCAACAATGGACCCTACCCGACTACGCTGGTCAGTTCACACACAACATGAACTTAGCACCAGCTGTTGCTCCCAACTTCCCTGGTGAGCAGCTCCTTTTCTTCCGCTCACATTTGCCATCTTCTGGTGGGCGATCTAACGGGATTCTAGACTGCCTGGTCCCCCAAGAATGGGTACAGCACTTCTACCAAGAGTCAGCCCCCTCTCAGTCTCAAGTGGCCCTGGTTAGGTATGTTAACCCTGACACTGGTAGAGTGTTATTTGAGGCCAAGCTACACAAATTGGGTTTCATGACTATAGCCAAGAATGGTGATTCTCCAATAATTGTTCCTCCAAATGGATACTTTAGGTTTGAATCTTGGGTGAACCCCTTTTACACACTTGCCCCCATGGGAACTGGGAATGGGCGTAGAAGGATTCAATAATGGCTG |
| KY424347.1 USA/1971/GII.6(P6) | ATGAAAACCCATATGAAAGTATGGTCCCCCACTCTCAGCGGGCCACACAGCTCATGGCCCTCCTTGGTGAAGCTTCATTGCACGGGCCCCAGTTTTACAAGAAGGTCAGCAAGATGGTTATCAGTGAGATCAAGAGTGGTGGTCTGGAGTTTTATGTGCCCAGACAAGAGGCCATGTTCAGGTGGATGAGATTCTCTGACCTCAGCACATGGGAGGGCGATCGCAATCTTGCTCCCGAGGGTGTGAATGAAGATGGCGTCKAAYGACGCTKCTCCATCGAATGATGGTGCTGCCAACCTCGTACCAGAGGCCAACAATGAGGTTATGGCACTTGAACCGGTGGTGGGAGCTTCAATCGCAGCTCCTGTTGTCGGTCAGCAAAATATAATTGACCCCTGGATTAGAGAAAATTTTGTCCAAGCACCACAGGGCGAGTTCACTGTTTCACCAAGGAACTCGCCTGGTGAGATGCTCTTAAACCTTGAGTTGGGCCCAGAGCTTAACCCCTATCTAAGTCATTTGTCCCGCATGTATAACGGGTATGCTGGTGGCATGCAGGTTCAGGTGGTCCTGGCTGGGAATGCGTTCACAGCTGGGAAAATCATCTTTGCCGCCGTACCACCACATTTCCCCGTAGAAAATATCAGTGCAGCCCAGATAACTATGTGCCCCCATGTAATTGTTGATGTGAGACAACTTGAACCAGTGCTTCTGCCCCTCCCTGACATAAGGAACAGATTCTTCCATTACAATCAGGAGAACACCCCCCGGATGAGACTTGTAGCTATGCTTTACACACCTCTGAGAGCCAACTCTGGTGAGGATGTGTTTACTGTATCTTGCAGGGTTTTGACTCGTCCTGCTCCAGATTTTGAGTTCACTTTCTTGGTGCCACCAACTGTTGAATCAAAGACTAAGCCTTTTACACTGCCTATATTAACTCTTGGTGAATTGTCCAATTCTAGGTTCCCAGCCCCAATAGATATGTTATACACTGACCCAAATGAGGGGATTGTAGTCCAACCACAAAATGGTAGGTGCACTCTTGATGGCACTCTGCAGGGTACCACACAATTAGTCCCCACCCAGATATGTGCTTTCAGGGGCACGCTAATTGGCCAAACCTCAAGATCTTCAGACTCAACTGATTCAGCTCCCCGAAGAAGGGATCACCCACTCCACGTGCAATTAAAGAATCTTGATGGCACGCAGTATGACCCAACTGATGAGGTGCCAGCGGTCCTTGGTGCCATTGATTTCAAGGGGGCCGTCTTTGGAGTAGCCAGCCAGAGGGATGTTTCAGGGACTCAAGTGGGGGCAACTCGTGCTCATGAAGTGCACATCAACACAACAGATCCCAGGTACACACCAAAACTGGGATCCATTCTCATGCACTCGGAGTCGGACGACTTTGTGACTGGGCAGCCAATCCAATTCACACCCGTGGGAATGGGTGATAACGACTGGCACCAGTGGGAGTTGCCCGATTACTCTGGACACCTAACCCTGAATATGAATCTTGCCCCAGCAGTCGCGCCTGCCTTCCCAGGTGAGAGGATCCTTTTCTTTAGATCAATAGTCCCATCTGCCGGCGGCTATGGGTCTGGACAAATAGATTGTCTCATACCACAGGAGTGGGTCCAGCATTTCTATCAGGAAGCTGCACCATCCCAGTCTGCTGTGGCTCTCATCAGGTATGTCAACCCTGACACGGGCAGAAACATCTTTGAGGCAAAATTGCACAGGGAAGGCTTCATCACTGTGGCAAATTCTGGTAACAACCCCATTGTTGTCCCCCCTAATGGGTACTTTAGGTTTGAAGCTTGGGTGAATCAATTTTACACTTTGACCCCCATGGGAACTGGTCAGGGGCGCAGGAGAAACCAATAATGGCTA |
| MT731279.1 USA/2018/GII.6(P7) | ATGAGAACCCATATGAGAGCATGGTCCCTCATTCCCAGCGGGCCACACAGCTTATGGCCCTTCTCGGTGAGGCTTCACTGCATGGCCCCCAGTTTTACAAGAAGGTCAGCAAGATGGTCATTAACGAAATCAAAAGTGGTGGTCTGGAATTCTATGTGCCCAGACAAGAGGCCATGTTCAGATGGATGAGATTCTCTGACCTCAGCACATGGGAGGGCGATCGCAATCTTGCTCCCGAAGGTGTGAATGAAGATGGCGTCGAATGACGCTGCTCCATCGAATGATGGTGCTGCCAACCTCGTACCAGAGGCCAACAATGAGGTTATGGCACTTGAGCCGGTGGTGGGGGCTTCAATCGCAGCTCCTGTCGTCGGCCAACAAAACATAATTGACCCCTGGATTAGAGAAAATTTTGTTCAAGCACCACAGGGTGAGTTCACCGTCTCGCCAAGGAACTCGCCTGGTGAGATGCTATTAAATCTTGAATTAGGCCCAGAACTCAACCCTTACCTAAGTCACCTGTCCCGTATGTATAATGGGTATGCTGGTGGCATGCAGGTTCAGGTGGTCCTAGCTGGGAATGCGTTCACAGCTGGGAAAATCATCTTTGCCGCTGTGCCACCACATTTCCCCGTGGAAAATATCAGTGCAGCTCAAATAACTATGTGCCCCCATGTGATTGTTGATGTGAGACAACTTGAGCCAGTACTCCTACCCCTTCCTGATATAAGGAATAGGTTCTTTCATTACAATCAGGAGAACACTTCCCGGATGAGACTTGTGGCCATGCTTTACACCCCCCTGAGGGCCAACTCTGGTGAAGATGTGTTTACTGTCTCTTGTAGGGTCTTAACCCGTCCTGCCCCTGATTTTGAATTTACTTTCTTGGTGCCGCCAACTGTTGAATCAAAGACTAAACCTTTTACATTACCTATATTAACTCTTGGTGAGCTATCTAATTCCAGATTTCCAGCCCCAATAGATATGTTGTACACTGATCCAAATGAGGGAATTGTGGTCCAACCACAAAATGGTAGGTGCACTCTTGATGGCACTTTGCAAGGCACCACACAACTGGTCCCCACCCAAATTTGTGCTTTCAGGGGCACACTAATTGGCCAAACATCAAGATCTTCAGACTCAACCGACTCAGCCCCTCGGAGGAGGGATCACCCACTCCATGTGCAATTAAAGAACCTTGATGGCACGCAGTATGACCCTACTGATGAAGTGCCAGCAGTCCTCGGTGCCATTGATTTTAAGGGGACTGTTTTTGGGGTGGCCAGTCAGAGGGACGTGTCAGGAGGTCAGGTGGGAGCAACTCGAGCCCATGAAGTGCACATCAACACAACCGATCCTAGGTATACACCAAAACTAGGGTCCATTCTCATGCACTCAGAGTCGGATGACTTCGTGACTGGACAGCCGGTCCGCTTCACACCCATAGGAATGGGCGACAACGACTGGCACCAGTGGGAGCTGCCCGACTATTCTGGACACCTAACCCTAAACATGAACCTTGCCCCAGCAGTTGCTCCTGCATTCCCGGGTGAGAGGATTCTTTTCTTCAGATCAATTGTCCCGTCTGCTGGTGGCTACGGCTCTGGGCAAATAGATTGCCTCATACCACAGGAGTGGGTTCAGCATTTCTACCAAGAAGCTGCACCATCCCAATCTGCCGTGGCACTCATCAGGTATGTCAACCCTGACACAGGCAGAAACATCTTTGAGGCTAAATTGCATAGGGAAGGTTTCATCACAGTGGCTAATTCTGGCAACAACCCCATTGTTGTTCCCCCTAATGGGTATTTTAGGTTTGAGGCTTGGGTGAATCAATTTTACACTTTGACCCCCATGGGAACTGGTCAGGGGCGTAGGAGGAATCAATAATGGCCA |
| MT712147.1 USA/2014/GII.8(P8) | ATGAGGATCCCGCTGAAACAATGATACCACACTCCCAAAGACCCATACAGTTGATGTCCCTATTAGGTGAAGCTGCATTACATGGCCCATCATTCTACAGCAAAATCAGCAAATTGGTCATCTCAGAGTTAAAAGAAGGTGGCATGGACTTTTACGTGCCAAGGCAAGAGCCAATGTTTCGATGGATGAGGTTCTCAGACTTGAGCACGTGGGAGGGCGATCGCAATCTGGCTCCCAATTTTGTGAATGAAGATGGCGTCGAATGACGCCGCTCCATCTAATGATGGTGCAGCCGGTCTCGTACCAGAGGTCAACAATGAGACCATGGCACTTGAACCGGTGGCTGGGGCTTCAATAGCCGCCCCACTCACCGGTCAAAATAATGTTATAGACCCCTGGATTAGATTAAACTTTGTGCAAGCTCCCAATGGAGAGTTTACAGTTTCACCCCGCAATTCGCCTGGGGAAGTCCTACTAAACTTGGAATTAGGCCCTGAACTAAATCCATATTTGGCACACCTTTCCAGAATGTACAATGGTTATGCAGGTGGAGTTGAGGTGCAAGTACTACTGGCTGGGAACGCGTTCACGGCTGGAAAACTGGTATTCGCCGCAGTTCCCCCTCATTTTCCATTAGAAAACATAAGCCCTGGCCAAATAACCATGTTCCCTCATGTAATTATTGATGTTAGGACTTTAGAACCAGTCCTATTGCCTCTTCCAGATGTTAGGAACAATTTCTTTCACTACAATCAGCAAAATGAACCGAGGATGAGGCTTGTGGCAATGCTCTATACCCCCCTTAGGTCTAATGGTTCTGGTGATGATGTATTTACTGTCTCCTGTAGAGTGCTTACTCGACCCTCTCCTGATTTTGATTTCAATTATTTGGTACCCCCTACAGTTGAATCTAAAACTAAACCCTTCACACTCCCCATCTTGACCATAGGGGAGTTAACCAATTCCAGATTCCCTGTGCCTATAGATGAACTCTACACTAGCCCCAATGAGAGTTTGGTGGTACAACCCCAGAACGGGAGATGCGCGCTGGATGGGGAGTTGCAGGGTACTACTCAGCTCCTCCCCACGGCGATCTGCTCGTTCAGGGGTCGGATTAATCAGAAAGTGAGTGGAGAGAACCATGTTTGGAATATGCAAGTCACCAACATTAACGGAACCCCTTTTGATCCAACAGAGGATGTTCCAGCTCCTCTAGGTACACCAGACTTTTCTGGCAAGCTCTTTGGCGTCCTAAGCCAGAGGGACCATGATAATGCCTGTAGGAGCCATGATGCAGTGATTGCAACTAATTCTGCTAAATTTACCCCAAAATTGGGCGCTATACAAATTGGCACATGGGAACAAGATGATGTACACATCAACCAACCCACCAAATTTACTCCAGTTGGCTTGTTTGAAAGTGAAGGTTTTAATCAGTGGACACTCCCTAATTATTCTGGAGCCTTAACACTCAACATGGGGTTGGCCCCCCCTGTGGCCCCCACATTTCCTGGCGAGCAAATTCTTTTCTTCAGATCCCACATTCCTCTTAAAGGAGGTGTGGCAGACCCAGTTATTGATTGTCTCTTGCCCCAAGAGTGGATCCAACACCTTTATCAGGAGTCAGCCCCTTCACAAACAGATGTAGCACTGATCAGATTCACAAATCCAGACACAGGGCGTGTCCTATTTGAAGCAAAATTGCACAGGAGTGGCTACATCACAGTGGCCAACACTGGTAGCAGACCAATTGTGGTGCCAGCTAATGGTTACTTCAGGTTTGACTCTTGGGTTAATCAGTTTTATTCTCTCGCCCCCATGGGAACTGGGAATGGGCGCAGAAGGGTGCAGTAATGGCTG |
| HQ449728.1 USA/2010/GII.12(P33) | ATGAGGATCCCGCTGAAACAATGATACCACACTCCCAAAGACCCATACAGTTGATGTCCCTATTAGGTGAAGCTGCATTACATGGCCCATCATTCTACAGCAAAATCAGCAAATTGGTCATCTCAGAGTTGAAAGAAGGTGGTATGGACTTTTACGTGCCAAGGCAAGAGCCAATGTTTCGATGGATGAGGTTCTCAGACTTGAGCACGTGGGAGGGCGATCGCAATCTGGCTCCCAATTTTGTGAATGAAGATGGCGTCGAATGACGCCGCTCCATCTAATGATGGTGCAGCCGGTCTCGTACCAGAGGTCAACAATGAGACCATGGCACTTGAACCGGTGGCTGGGGCTTCAATAGCCGCCCCACTCACCGGTCAAAATAATGTTATAGACCCCTGGATTAGATTAAACTTTGTGCAGGCCCCCAATGGAGAGTTTACAGTTTCACCCCGCAATTCGCCTGGGGAAGTCCTACTAAACTTGGAATTAGGCCCTGAACTAAATCCATATTTGGCACACCTTTCCAGAATGTACAATGGTTATGCAGGTGGAGTTGAGGTGCAAGTACTACTGGCTGGGAACGCGTTCACGGCTGGAAAACTGGTATTCGCCGCAGTTCCCCCTCATTTTCCATTAGAAAACATAAGCCCTGGCCAAATAACCATGTTCCCTCATGTAATTATTGATGTTAGGACTCTAGAACCAGTCCTATTGCCTCTTCCAGATGTTAGGAACAATTTCTTTCACTACAATCAGCAAAACGAACCAAGGATGAGACTTGTGGCAATGCTCTATACCCCCCTTAGATCTAATGGTTCTGGTGATGATGTATTCACTGTCTCCTGTAGAGTGCTTACTCGACCCTCTCCTGATTTTGATTTCAATTATTTGGTACCCCCTACAGTTGAATCTAAAACTAAACCCTTCACACTCCCTATCTTGACCATAGGGGAGTTAACCAATTCCAGATTCCCTGTGCCTATAGATGAACTCTACACTAGCCCCAATGAGAGTTTGGTGGTACAACCCCAGAACGGGAGATGCGCGCTGGATGGGGAGTTGCAGGGTACTACTCAGCTCCTCCCCACGGCGATCTGCTCGTTCAGGGGTCGGATTAATCAGAAAGTGAGTGGAGAGAACCATGTTTGGAATATGCAAGTCACCAACATTAACGGAACCCCTTTTGATCCAACAGAGGATGTTCCAGCTCCTCTAGGTACACCAGACTTTTCTGGCAAGCTCTTTGGCGTCCTAAGCCAGAGGGACCATGACAATGCCTGTAGGAGCCATGATGCAGTGATTGCAACTAATTCTGCTAAATTCACCCCAAAATTGGGCGCTATACAAATTGGCACATGGGAACAAGATGATGTACACATCAACCAACCCACCAAATTTACTCCAGTTGGCTTGTTTGAAAGTGAAGGTTTTAATCAGTGGACGCTCCCCAATTATTCTGGAGCCTTAACACTCAACATGGGGTTGGCCCCCCCTGTGGCCCCCACATTTCCTGGCGAGCAAATTCTTTTCTTCAGATCCCACATTCCTCTTAAAGGAGGTGTGGCAGACCCAGTTATTGATTGTCTCTTGCCCCAGGAGTGGATCCAACACCTTTATCAGGAGTCAGCCCCTTCACAAACAGATGTAGCACTGATCAGATTCACAAATCCAGACACAGGGCGTGTCCTATTTGAAGCAAAATTGCACAGGAGTGGCTACATCACAGTGGCCAACACTGGTAGCAGACCAATTGTGGTGCCAGCTAATGGTTACTTCAGGTTTGACTCTTGGGTTAATCAGTTTTATTCTCTCGCCCCCATGGGAACTGGGAATGGGCGCAGAAGGGTGCAGTAATGGCTG |
| GU594162.1 JPN/2008/GII.14(P7) | ATGAGAACCCGTACGAGAGCATGGTCCCTCACTCCCAGCGGGCCACACAGCTTATGGCCCTTCTTGGTGAGGCTTCACTGCATGGTCCCCAGTTTTACAAGAAAGTTAGCAAGATGGTTATTAATGAGATTAAGAGTGGTGGTCTGGAATTTTATGTGCCCAGACAGGAGGCCATGTTCAGGTGGATGAGATTTTCTGACCTCAGCACATGGGAGGGCGATCGCAATCTTGCTCCCGAAGGTGTGAATGAAGATGGCGTCGAATGACGCTACTCCATCTGATGATGGTGCAGCCGGCCTCGTGCCAGAGATCAACAATGAGGTTATGGCTCTTGAACCCGTCGCTGGGGCCTCCATCGCAGCCCCCGTAGTCGGCCAACAGAATATAATTGATCCCTGGATTAGAAATAATTTTGTACAAGCCCCTGCTGGTGAATTCACAGTTTCCCCTAGAAACTCTCCTGGAGAACTTCTACTTGATTTGGAATTGGGTCCTGAACTCAATCCCTATCTTGCACATTTGGCCAGGATGTACAATGGGCATGCAGGAGGAATGGAAGTGCAGATAGTACTGGCTGGGAATGCGTTCACAGCTGGTAAGATCCTATTTGCTGCCATCCCGCCTAGCTTCCCCTATGAAAATTTGTCACCCGCTCAATTGACTATGTGCCCCCATGTGATAGTGGATGTGAGGCAATTAGAACCAGTGCTTCTGCCAATGCCAGATATAAGAAATGTTTTCTACCACTATAACCAGAACAATAGTCCCAAACTTAGGCTTGTAGCTATGCTTTATACCCCTCTGAGGGCTAACAACTCAGGTGATGACGTGTTCACGGTGTCTTGCCGCGTTTTAACACGTCCTTCTCCAGATTTCCAGTTCACCTTTCTGGTCCCACCTACAGTTGAGTCTAAAACTAAGAATTTCACCCTTCCCGTTCTTAGAGTCTCAGAGATGACAAACTCAAGGTTCCCCGTTGTTTTAGACCAAATGTATACAAGCAGGAATGAAAACATCATTGTCCAACCCCAAAACGGCAGGTGCACGACTGACGGTGAGCTGCTTGGCACTACCATCTTGCAGTCTGTGTCTATTTGCAACTTTAAAGGAACAATGCAGGCAAAGCTGAATGAAGAACCACGATACCAACTACAACTCACCAACTTGGATGGGTCACCCATAGATCCAACAGATGATATGCCTGCCCCTCTTGGCACACCAGACTTCCAGGCCATGTTATATGGCGTTGCAAGCCAACGCTCTTCCATAGACAATGCCACCAGGGCACATGATGCACAGATTGACACTGCGGGTGACACATTCGCCCCGAAAATTGGCCAGGTTCGTTTTAAATCAAGCTCTAATGATTTTGACCTACATGACCCTACAAAATTCACACCTATTGGTGTCAATGTGGATGATCAGCACCCTTTTAGACAGTGGTCCCTGCCAAACTATGGTGGTCACCTTGCCCTGAACAACCATTTAGCCCCCGCTGTGACACCACTCTTTCCTGGCGAGCAGATTTTGTTCTTCAGGTCATACATTCCGAGCGCCGGAGGCCACACAGACGGTGCTATGGACTGCTTGCTGCCCCAAGAGTGGGTAGAGCACTTCTACCAGGAAGCGGCTCCTTCCCAATCTGACATTGCACTGGTAAGGTTCATCAACCCTGATACAGGAAGAGTGCTCTTTGAAGCTAAATTGCATAAACAAGGTTTCCTCACAATTGCAGCATCTGGAGATCACCCTATTGTGATGCCCACTAATGGTTACTTTAGGTTTGAAGCTTGGGTCAACCCTTTCTACACTCTCGCCCCCGTGGGAACTGGGTCTGGGCGCAGGAGGATTCAATAATGGCTG |
| MN447470.1 USA/2008/GII.16(P16) | ATGAAGACCCCAATGAAACAATGATACCCCACTCTCAAAGACCCATACAGCTCATGGCACTGCTTGGCGAGGCTTCCCTCCATGGACCCTCTTTCTATAGCAAGATTAGTAAATTGGTCATAACTGAACTCAAAGAAGGTGGGATGGACTTTTACGTGCCAAGGCAGGAACCCATGTTCAGGTGGATGAGGTTTTCTGACTTGAGCACGTGGGAGGGCGATCGCAATCTGGCTCCCAGTTTTGTGAATGAAGATGGCGTCGAATGACGCCGCTCCATCTAATGATGGTGCAGCCGGTCTCGTACCAGAGGTCAACAACGAGACAATGGCACTAGAGCCAGTTGCAGGAGCTTCAATTGCTGCCCCTTTAACTGGTCAAAACAATGTAATAGACCCCTGGATTAGATTAAATTTTGTACAAGCCCCCAATGGTGAGTTCACGGTTTCCCCCCGAAACTCGCCTGGTGAAATTTTGTTAAATTTGGAGTTAGGGCCTGAATTAAATCCATACTTAGCCCATTTAGCAAGGATGTATAATGGTTATGCAGGTGGGGTTGAGGTGCAGGTACTGCTGGCTGGAAATGCGTTTACAGCAGGCAAACTTGTATTTGCAGCAATCCCCCCTCATTTTCCAGTAGATAATTTGAGCCCTGGCCAGATTACTATGTTCCCCCATGTGATTATTGATGTTAGAACACTTGAACCTGTCTTGTTACCATTACCTGATGTTAGAAATAATTTCTTTCACTATAATCAACAGAGTGATCAGCGCATGAGACTTGTTGCTATGCTTTATACCCCTCTTAGATCTAATGGTTCAGGTGATGATGTCTTCACTGTGTCATGCAGGGTCCTAACCAGGCCATCCCCTGATTTTGATTTTAATTATCTGGTTCCTCCAACAGTTGAATCTAAAACTAAACCCTTCTCAGTCCCAGTGCTAACCCTTAATGAGCTGACCAACTCTCGTTTTCCTGTTCCGATTGATGCCATGTACACCAGCCCTAATGACTCAATTGTGGTCCAACCTCAAAATGGAAGGGCAACCATAGACGGTGAGTTGCTTGGCACCACTCAACTCATACCCAGTGGTATCTGTTCATTTAGGGGAAAAATCACAACACATTTGTCAGATGATAGGCACTTGTGGAACATCCAAGTCTCCAATTTGAATGGCACTCCGTTTGACCCAACAGACGACGTGCCCGCACCATTGGGTATGCCAGACTTCTCTGGGCAAATATTTGGGGTGGTTAGTCAAAGGGACACAGGCACCAACCCCGCAAACAGAGCTCATGACGCTGTTCTTGCAACCTACAGCGCCAAATACACACCTAAGCTCGGCTCAGTCCAAATTGGAACATGGGACACCGAGGATTTGCTTGAAAGACAGCCTGTCAAGTTCACGCCCGTTGGTCTCAATGAAATAGGGCAAGACAAGCATTTCGACCAATGGGTGTTGCCAAATTATTCTGGTGCCCTGGGACTCAACATGCACCTGGCTCCTGCTGTATCCCCACTCTTTCCCGGAGAGAGATTATTGTTCTTTAGATCATATATACCTCTAAAAGGTGGCCATGGGGACCCATTTATTGATTGTCTTGTGCCACAAGAGTGGATACAACATTTTTACCAAGAATCAGCCCCAGCACAATCCTCGGTGGCTCTACTGAGGTATGTGAATCCAGACACAGGTCGCACTCTATTTGAGGCCAAATTACACAAGGAAGGGTTTATCACCGTCTCTAGCACGGAAAATAGGCCTGTGATTGTCCCCCCAAATGGTTACTTTAGGTTTGACTCTTGGGTTAACCAATTTTACTCATTAGCCCCCATGGGAACTGGCAATGGGCGCAGAAGGATGCAGTAATGGCTG |
| KU561249.1 CHN/2015/GII.17(P17) | ATGAAGACCCCAGTGAGACCATGATACCACACGCACAAAGACCTGTGCAGCTCATGGCACTACTAGGAGAATCCTCCCTACATGGACCCTCATTTTACAGCAAGGTTAGCAAATTAGTCATATCTGAACTTAAAGAGGGAGGAATGGATTTTTATGTGCCCAGACAAGAGTCAATGTTCAGATGGATGAGGTTCTCAGATCTAAGCACATGGGAGGGCGATCGCAATCTGGCTCCCAGTTTTGTGAATGAAGATGGCGTCGAATGACGCCGCTCCATCTAATGATGGTGCTGCTGGTCTCGTACCAGAGGGCAACAACGAGACCCTTCCCCTAGAACCAGTTGCGGGCGCAGCTATAGCCGCACCCGTCACTGGCCAAAATAACATAATTGACCCCTGGATTAGAACAAATTTTGTGCAAGCACCAAATGGAGAGTTCACAGTGTCACCCAGAAACTCTCCTGGAGAAATTTTATTAAACTTAGAGTTGGGCCCTGATTTGAACCCTTATTTGGCTCATTTGTCAAGGATGTACAATGGGTATGCTGGTGGAGTGGAAGTTCAGGTTCTCCTGGCAGGGAACGCGTTCACTGCCGGAAAGATCCTCTTCGCCGCCGTCCCGCCAAATTTCCCAGTGGAATTCTTAAGCCCAGCCCAGATCACAATGCTCCCACATTTAATAGTAGATGTTAGGACTCTTGAACCAATTATGATCCCACTCCCTGATGTTAGGAATACATTCTTCCATTATAGTAACCAGCCTAACAGCCGCATGAGATTAGTGGCTATGCTCTATACCCCACTCAGATCTAATGGCTCAGGTGATGATGTCTTTACTGTCTCTTGCAGGGTTTTGACTAGGCCTACTCCTGATTTTGAGTTCACTTATTTAGTGCCACCTTCTGTTGAATCTAAAACTAAGCCTTTTTCCTTACCTATTTTAACCCTTTCTGAGCTCACAAATTCGAGGTTCCCAGTCCCCATCGATTCGCTTTTCACCGCCCAGAATAATGTGTTGCAGGTGCAGTGTCAAAATGGCAGGTGTACACTTGATGGTGAGTTACAAGGCACAACCCAGTTGCTCCCATCTGGCATCTGTGCATTCAGAGGACGGGTGACAGCACAAATTAACCAACGTGACAGGTGGCACATGCAACTGCAAAACCTCAATGGTACAACATATGACCCAACTGATGATGTGCCAGCCCCGCTGGGTACACCTGACTTCAAGGGCGTCGTGTTTGGGATGGTAAGCCAAAGAAATGTGGGTAATGATGCGCCTGGCTCAACCAGAGCCCAACAGGCGTGGGTTTCAACCTATAGCCCCCAATTTGTCCCCAAATTAGGTTCTGTCAATCTTAGAATTAGTGATAATGATGATTTCCAATTCCAGCCGACAAAATTCACACCAGTGGGCGTCAATGATGACGATGATGGCCACCCGTTCAGACAATGGGAACTACCAAACTATTCAGGGGAGCTTACCTTGAATATGAATCTTGCCCCCCCAGTTGCTCCAAATTTTCCTGGTGAACAATTGTTATTCTTCAGATCTTTCGTGCCATGCTCAGGAGGTTACAACCAAGGTATTATAGATTGTCTTATTCCCCAAGAATGGATCCAACACTTCTATCAGGAATCAGCACCCTCCCAGTCAGACGTGGCCCTAATCAGGTATGTCAACCCCGATACGGGACGTACACTGTTTGAAGCAAAATTGCACAGATCTGGTTACATTACTGTGGCTCACTCTGGAGACTATCCTCTTGTTGTTCCGGCTAATGGACACTTTAGATTTGATTCTTGGGTAAATCAGTTTTACTCACTCGCCCCAATGGGAACTGGGAATGGGCGAAGGAGGGCTCAGTAATGGCTG |
| MN453358.1 PER/2011/GII.23(P23) | ATGAGGACCCCAATGAAACAATGATCCCACATTCCCAGAGGCCAGTGCAATTGATGTCCCTGCTTGGTGAGGCGGCCCTACACGGACCGACATTCTACAAGAGAGTTAGCAAGTTGGTCATCACTGAATTAAAGGAAGGTGGAATGGATTTCTATGTGCCCAGACAAGAACCTATGTTTAGATGGATGAGATTCTCTGACTTGAGCACGTGGGAGGGCGATCGCAATCTGGCTCCCGAAAGTGTGAATGAAGATGGCGTCGAATGACGCCGCTCCATCTAATGATGGTGCAGCCGGTCTCGTACCAGAGATCAACAACGAGACAATGGCTTTGGAACCAGTGGCAGGAGGAGCAATTGCAGCCCCACTAACTGGACAAACAAATTTCATTGATCCCTGGATTAGAGGTAATTATGTGCAAGCACCAAATGGTGAATTTACAGTGTCCCCCAGAAATTCGCCCGGTGAAATTTTGCTTAATTTAGAATTAGGCCCTGAACTCAACCCATTCTTGTCCCATCTATCTAGAATGTACAATGGGTATGCTGGCGGTATTGATGTCCAAGTCATCATGGCAGGCAATGCCTTCACCGCTGGTAAGATCATCTTTGCAGCAATACCTCCCCATTTCCCCGTGGAAAATCTTAGTCCCCCTCAGATCACTATGTTCCCTCATATTATAGTGGATGTCAGGACCCTTGAACCCATAAATATCCCTGTACCAGATGTTAGGAACAACTTCTTCCATTATAATCAAAACAATGAGTCTCGCATGAGACTGGTGGCCATGTTGTATACCCCTCTTAGGTCTAACGGGTCTGCTGAAGATGTTTTCACAGTGTCATGTAGGGTCTTAACAAAACCATCTGCAGACTTTGAATTTAATTATCTAGTCCCCCCAACTGTAGAGTCAAGGACTAAACCCTTCACAATACCAATACTCACAATTGGGGAAATGACAAATTCAAGATTCCCAGTGCCTATTGACATGCTCCATACTAGTCCAACAGAAAACATTGTTGTTCAACCACAAAATGGCAGGTGCACCATAGATGGTGAGTTGCTTGGCACCACTCAACTGATCACTTCCAATATCTGTTCATTCAGAGGATCCATCTCAGGGCATGAAGGCAATGATGACGAACACCTGTGGCACTTCAACATCACAAACCCCAATGGCACTCCATTTGACCCCACTGAAGATGTGCCTGCCCCCCTCGGAACCCCAGATTTCAGGGGTCAATTGTACGGTGTCATCTCCCAGCGAGACAGAGAGGGAACACCCTCGGCGAACAATCAGAAGGCAAACAGGTCCCACCAGGGAGTGATTTCAACAATCTCACCAAAATTTGTCCCTAAGTTAGGTTCAGTCATGATTGGCACATGGACTACTGATGATATCAAGAACCAGCCATCCAAGTTCGCCCCAATTGGCCTCAATGATGATGATAATTTTAAACAATGGGAACTGCCAAATTATTCTGGGGCGCTCACCCTCAACATGGGGTTGGCACCTAGTGTTTTCCCCACCTATCCTGGTGAACAAATCCTTTTCTTCCGCTCCTATCTGCCAATGAAGGGAGGGTACGGTAGTCCATTTATTGACTGCCTAGTACCTCAAGAATGGGTCGCTCACTTCTACCAGGAGTCAGCACCCGCACAAACAGATGTGGCCCTAATCAGGTATGTAAACCCCGAAACAGGACGTGTCCTTTTCGAGGCCAAATTGCACAGACAGGGGTTCATTACCGTTTCCAAGAGCGGCGACAGCCCCATCAATGTGCCTGCCAATGGTTATTTTAGGTTTGATTCTTGGGTTAATCAGTTTTACTCTCTTGCCCCCATGGGAACTGGGAACGGGCGCAGAAGGATTCAGTAATGGCTG |
| MN453359.1 PER/2013/GII.24(P24) | ATGAAGACCCTAGTGAAACAATGATACCACATTCCCAGAGGCCTGTGCAGCTGATGTCCCTACTAGGTGAAGCAGCCCTACATGGACCAGCTTTCTACAAAAAGGTCAGCAAATTGGTTATCACTGAACTCAAAGAAGGTGGAATGGATTTCTATGTGCCCAGGCAAGAGCCTATGTTTAGATGGATGAGGTTCTCCGATCTGAGCACGTGGGAGGGTGATCGCAATCTAGCTCCCGAGGGTGTGAATGAAGATGGCGTCGAATGACGCTGCTCCATCTAATGATGGTGCTGCCAACCTCGTACCAGAGGCCAACAAAGAGGTTATGGCATTGGAACCAGTGGCGGGCGGGGCAATCGCCGCACCCCTCACTGGTCAAACAAATATAATTGACCCCTGGATCATGAATAATTTTGTGCAAGCACCAAATGGTGAATTTACAATATCTCCCAGAAATTCCCCAGGAGAAGTGTTGTTAAATCTAGAGTTAGGACCAGACCTCAACCCTTTCCTTGCCCATTTGTCTAGAATGTATAATGGATATGCGGGGGGAGTGGAGGTGCAAGTGATTATGGCTGGCAACGCTTTCACCGCGGGAAAAGTCATCTTCGCAGCTGTACCACCGCACTTCCCAGTTGACAACCTCAGCCCCCCTCAGGTGACTATGTTTCCACATGTTATAGTTGATGTTAGGACCTTTGAGCCAATCCTACTTCCCCTTCCAGATGTGAGAAATAGCTTCTATCATTATAATCAGGTGAATGATTCAAGGATGAGGTTGATTGCTATGCTGTATACCCCACTGAGATCAAATGGTTCTTCCGATGATGTTTTCACTGTATCCTGTAGAGTTTTGACTAGGCCCACCCCAGATTTTGAGTTCAACTACCTGGTCCCACCTACTGTCGAATCAAGAACAAAACCCTTCAGTGTGCCCATCCTAACTATTGGAGAAATGACAAATTCAAGGTTTCCACTGCCCATAGACATGCTGTACACCAGCCCAACTGAAAACATAGTAGTTCAGCCCCAAAATGGTAGGTGCACGTTGGAAGGAGAACTACTGGGCACAACCCAGTTAGTGACCCCGAACATCTGCGCGCTACGTGGGGAGATCAGGGGGCATGAAGGCAGTGGTGACAACCACAAATGGCATTTTATGGTGAGGAGCCCCAATGGGGCAGCTTTTGACCCAACTGAGGATGTGCCCGCCCCCCTAGGCACCCCTGATTTCATAGGTGATGTGTTTGGAGTCCTCTCACAAAGAAACAGAAACACTGACAGTGGTCAGTCTGGTCCTGCAAACAGATCACATGATGCTGTTGTTTCCACAAGGGACAGCAGATTCACTCCAAAACTAGGATCTGTGATGATAGCCACCTGGGAGACCAGTGACATCCAAGACCAGCCCACCAGATTCACTCCAGTAGGCCTTGAAAACCCTGATCATTACAACCAATGGCAATTACCAAATTATTCAGGGGCATTAACTCTAAACATGGGGCTGGCCCCATCTGTGTTTCCAACCTACCCCGGAGAGCAAATCCTCTTCTTCCGCTCGTACATCCCACTTAAGGGCGGATATGGAGATTCCCACATTGATTGCCTGGTACCACAGGAATGGATCCAACATTTCTACCAAGAATCAGCCCCCTCACAAACTGATGTGGCATTAATCAGGTATGTGAACCCCGAAACAGGACGTGTCCTGTTCGAGGCCAAGTTGCATAGGCAAGGTTACATAACAGTTGCAAGGAGTGGCAGCAGTCCCATCAATGTCCCGGCCAATGGCTATTTCAGATTCGACTCCTGGGTCAACCAATTCTACTCACTCGCCCCCATGGGAACTGGAAACGGGCGCAGAAGGATCCAATAATGGCAG |
| LC342059.1 JPN/1999/GII.12(PNA7) | ATGAAGACCCTTTTGAAACAATGATACCACATTCCCAAAGACCCATCCAGCTAATGTCGCTCTTGGGCGAAGCAGCCCTACATGGCCCATCATTCTACAGTAAAATCAGCAAATTGGTCATCACAGAGTTAAAGGAAGGTGGCATGGATTTTTACGTGCCCAGGCAAGAGCCCATGTTCAGGTGGATGAGGTTCTCAGACTTGAGCACGTGGGAGGGCGATCGCAATCTGGCTCCCAGTTTTGTGAATGAAGATGGCGTCGAGTGACGCCGCTCCATCTAATGATGGTGCAGCCGGTCTCGTACCAGAGGCTAACAATGAGACCATGGCACTTGAACCGGTGGCTGGGGCTTCAATAGCCGCCCCACTCACCGGCCAAAACAATGTTATAGACCCCTGGATTAGATTAAATTTTGTGCAGGCTCCCAATGGAGAGTTCACGGTTTCACCCCGTAACTCGCCTGGGGAAATCCTACTAAATTTGGAATTGGGCCCTGAACTGAATCCATACCTGGCACACCTTTCTAGAATGTACAATGGTTATGCAGGTGGGGTTGAGGTGCAAGTACTACTGGCTGGGAACGCGTTCACAGCTGGAAAACTGGTGTTTGCCGCAGTTCCCCCTCACTTTCCATTAGAAAATATAAGCCCTGGCCAAATAACTATGTTCCCTCATGTAATTATTGATGTTAGGACTTTAGAACCAGTTCTTTTGCCCCTCCCAGATGTCAGGAATAGTTTCTTTCATTACAATCAGCAAAATGAATCGCGGATGAGACTTGTAGCAATGCTTTATACTCCTCTTAGATCTAATGGCTCTGGTGATGATGTGTTCACTGTCTCTTGCAGGGTGCTTACTCGACCTTCCCCTGATTTTGATTTTAACTATTTGGTTCCCCCTACTATTGAGTCTAAAACTAAACCCTTCACACTCCCCATCTTGACTATAGGGGAGCTAACCAATTCCAGGTTCCCTGCGCCCATAGATGAGCTCTACACCAGTCCCAATGAGAGTTTGGTGGTGCAGCCCCAAAACGGGAGATGCGCGCTGGACGGGGAGTTGCAGGGCACGACTCAGCTCCTCCCCACGGCGATCTGCTCGTTCAGGGGTCGAATCAACCAGAAGGTAAGTGGAGAGAACCATGTCTGGAATATGCAGGTCACCAACACCAACGGAACCCCTTTTGATCCAACAGAAGATGTCCCGGCCCCTCTAGGCACTCCAGACTTCTCTGGCAAGCTCTTTGGTGTTTTGAGCCAGAGGGACCACGATAATGCATGTAGGAGCCATGATGCAGTGATTGCAACCAACTCTGCCAAATTCACCCCAAAATTGGGCGCTATACAAATTGGCACATGGGAAGAAGATGATGTACACATCAACCAACCTACCAAATTTACTCCAGTTGGCTTATTTGAACATGAAGATTTCAGTCAGTGGACACTCCCCAACTATTCTGGTGCCTTAACACTTAATATGGGATTAGCCCCTCCTGTTGCCCCCACCTTTCCTGGTGAACAAATTCTCTTCTTTAGATCCCACATTCCTCTTAAAGGAGGTGTAGCAGACCCAGTTATTGATTGTCTTTTGCCCCAGGAGTGGATTCAACATCTTTACCAAGAGTCAGCCCCTTCACAATCTGATGTAGCACTGATTAGGTTCACAAATCCAGACACAGGACGTGTTCTATTTGAGGCAAAATTACATAGGAGTGGTTACATTACAGTGGCCAACACTGGTAGCAGACCGATTGTGGTACCAGCTAACGGTTACTTCAGATTTGACTCTTGGGTTAATCAGTTCTATTCTCTCGCCCCCATGGGAACTGGGAACGGGCGCAGGAGGGTGCAGTAATGGCTG |
| M87661.2 USA/1968/GI.1(P1) | ATTCAGATCCATCAGAGACTCTAGTGCCACACACTCAAAGAAAAATACAGTTGATTTCACTTCTAGGGGAAGCTTCACTCCATGGTGAGAAATTTTACAGAAAGATTTCCAGCAAGGTCATACATGAAATCAAGACTGGTGGATTGGAAATGTATGTCCCAGGATGGCAGGCCATGTTCCGCTGGATGCGCTTCCATGACCTCGGATTGTGGACAGGAGATCGCGATCTTCTGCCCGAATTCGTAAATGATGATGGCGTCTAAGGACGCTACATCAAGCGTGGATGGCGCTAGTGGCTGGTACCGGAGGTTAATGCGACCCTCTTGCAATGGATCCTGTAGCAGGTTCTTCGACAGCAGTCGCGACTGCTGGACAAGTTAATCCTATTGATCCCTGGATAATTAATAATTTTGTGCAAGCCCCCCAAGGTGAATTTACTATTTCCCCAAATAATACCCCCGGTGATGTTTTGTTTGATTTGAGTTTGGGTCCCCATCTTAATCCTTTCTTGCTCCATCTATCACAAATGTATAATGGTTGGGTTGGTAACATGAGAGTCAGGATTATGCTAGCTGGTAATGCCTTTACTGCGGGGAAGATAATAGTTTCCTGCATACCCCCTGGTTTTGGTTCACATAATCTTACTATAGCACAAGCAACTCTCTTTCCACATGTGATTGCTGATGTTAGGACTCTAGACCCCATTGAGGTGCCTTTGGAAGATGTTAGGAATGTTCTCTTTCATAATAATGATAGAAATCAACAAACCATGCGCCTTGTGTGCATGCTGTACACCCCCCTCCGCACTGGTGGTGGTACTGGTGATTCTTTTGTAGTTGCAGGGCGAGTTATGACTTGCCCCAGTCCTGATTTTAATTTCTTGTTTTTAGTCCCTCCTACGGTGGAGCAGAAAACCAGGCCCTTCACACTCCCAAATCTGCCATTGAGTTCTCTGTCTAACTCACGTGCCCCTCTCCCAATCAGTAGTATGGGCATTTCCCCAGACAATGTCCAGAGTGTGCAGTTCCAAAATGGTCGGTGTACTCTGGATGGCCGCCTGGTTGGCACCACCCCAGTTTCATTGTCACATGTTGCCAAGATAAGAGGGACCTCCAATGGCACTGTAATCAACCTTACTGAATTGGATGGCACACCCTTTCACCCTTTTGAGGGCCCTGCCCCCATTGGGTTTCCAGACCTCGGTGGTTGTGATTGGCATATCAATATGACACAGTTTGGCCATTCTAGCCAGACCCAGTATGATGTAGACACCACCCCTGACACTTTTGTCCCCCATCTTGGTTCAATTCAGGCAAATGGCATTGGCAGTGGTAATTATGTTGGTGTTCTTAGCTGGATTTCCCCCCCATCACACCCGTCTGGCTCCCAAGTTGACCTTTGGAAGATCCCCAATTATGGGTCAAGTATTACGGAGGCAACACATCTAGCCCCTTCTGTATACCCCCCTGGTTTCGGAGAGGTATTGGTCTTTTTCATGTCAAAAATGCCAGGTCCTGGTGCTTATAATTTGCCCTGTCTATTACCACAAGAGTACATTTCACATCTTGCTAGTGAACAAGCCCCTACTGTAGGTGAGGCTGCCCTGCTCCACTATGTTGACCCTGATACCGGTCGGAATCTTGGGGAATTCAAAGCATACCCTGATGGTTTCCTCACTTGTGTCCCCAATGGGGCTAGCTCGGGTCCACAACAGCTGCCGATCAATGGGGTCTTTGTCTTTGTTTCATGGGTGTCCAGATTTTATCAATTAAAGCCTGTGGGAACTGCCAGCTCGGCAAGAGGTAGGCTTG |
| MG599789.1 GBR/2016/GI.2(P2) | ATTCAGACCCTTTTGAGACCCTGGTGCCACACCAACAGAGGAAGGTCCAACTAATATCATTATTGGGTGAGGCCTCACTGCATGGTGAAAAGTTTTACAGGAAGATTTCAAGTAAAGTCATCCAGGAAATTAAAACAGGGGGCCTTGAAATGTATGTGCCAGGATGGCAAGCCATGTTCCGTTGGATGCGGTTCCATGACCTTGGTTTGTGGACAGGAGATCGCAATCTCCTGCCCGAATTTGTAAATGATGATGGCGTCTAAGGACGCCCCTCAAAGCGCTGATGGCGCAAGCGGCTGGTGCCGGAGGTTAATACGACCCCTTACCCATGGAACCTGTGGCTGGGCCAACAACAGCCGTAGCCACTGCTGGGCAAGTTAATATGATTGATCCCTGGATTGTTAATAATTTTGTCCAGTCACCTCAAGGTGAGTTCACAATCTCTCCTAACAACACCCCCGGTGATATTTTGTTTGATTTACAACTAGGTCCACATCTAAACCCTTTCTTGTCACATTTGTCTCAAATGTATAATGGCTGGGTTGGGAACATGAGGGTCAGAATCCTCCTTGCTGGGAATGCATTCTCAGCTGGGAAGATTATAGTTTGTTGTGTCCCCCCTGGCTTTACATCTTCTTCTCTCACCATAGCTCAGGCTACATTGTTTCCCCATGTAATAGCTGATGTGAGAACCCTTGAGCCAATAGAAATGCCCCTCGAGGATGTACGCAACGTCCTCTATCACACCAATGACAATCAACCAACAATGCGGCTGGTGTGTATGCTATACACGCCGCTCCGCACTGGTGGGGGGTCTGGTAGTTCTGATTCCTTTGTAGTTGCTGGCAGGGTTCTCACAGCCCCTAGTAGCGACTTCAGTTTCTTGTTCCTTGTCCCGCCTACCATAGAACAGAAGACTCGGGCTTTCACTGTGCCTAATATCCCCTTGCAAACCTTGTCCAATTCTAGGTTTCCTTCCCTCATCCAGGGGATGATTCTGTCCCCCGATGCATCCCAAGTGGTTCAATTCCAAAATGGGCGTTGCCTTATAGATGGTCAACTCTTAGGCACTACACCCGCTACATCAGGACAGCTATTCAGAGTAAGAGGAAAGATAAATCAGGGAGCCCGCACACTTAACCTCACAGAGGTGGATGGTAAACCATTCATGGCATTTGATTCCCCTGCACCTGTGGGGTTCCCCGATTTTGGAAAATGTGATTGGCACATGAGAATCAGCAAAACCCCAAACAACACAAGTTCAGGTGACCCCATGCGCAGTGTCAGCGTGCAAACCAATGTGCAGGGTTTTGTGCCACATCTGGGAAGTATACAATTTGATGAAGTGTTCAATCATCCCACAGGTGACTACATTGGCACCATTGAATGGATTTCCCAGCCATCCACACCCCCTGGAACAGATATCAATCTGTGGGAGATCCCCGATTATGGATCATCCCTTTCCCAAGCAGCTAATCTGGCCCCCCCAGTATTCCCCCCTGGATTTGGTGAGGCCCTTGTATACTTTGTTTCTGCTTTCCCGGGCCCTAATAACCGCTCAGCCCCGAATGATGTACCCTGTCTTCTCCCTCAAGAGTACATAACCCACTTTGTCAGTGAACAAGCCCCAACGATGGGTGACGCAGCTTTACTGCATTATGTCGACCCTGATACCAACAGGAACCTTGGGGAGTTCAAGCTATACCCTGGGGGTTACCTCACCTGTGTACCAAACGGGGTAGGTGCCGGGCCTCAACAGCTCCCCCTCAATGGTGTTTTTCTCTTTGTTTCTTGGGTGTCTCGTTTTTACCAGCTTAAGCCTGTGGGAACAGCCAGTACGGCAAGAGGTAGGCTTG |
| MH218659.1 GBR/2015/GI.3(P3) | ATGAGGACCCATTTGAGACACTGGTTCCACATTCACAGAGAAAGGTCCAATTAATATCCTTGCTAGGTGAAGCAGCACTCCACGGTGAAAAGTTCTACAGGAAGATAGCTGGTAGGGTCATTCAAGAAGTCAAAGAGGGAGGGCTTGAAATCTACATTCCTGGTTGGCAGGCCATGTTCCGCTGGATGCGATTCCATGATCTAAGTTTGTGGACAGGAGACCGCGATCTCCTGCCCGATTATGTAAATGATGATGGCGTCTAAGGACGCCCCAACAAACATGGATGGCACCAGTGGTTGGTACCAGAGGCAAACACGAGCCTATATCAATGGAGCCCGTGGCTGGGGCAGCAACAGCTGCCGCAACTGCTGGCCAAGTTAATATGATTGACCCCTGGATAATGAACAATTATGTACAAGCCCCCCAAGGTGAATTTACCATATCGCCTAATAACACACCAGGTGATATTTTGTTTGATTTACAATTAGGCCCCCACCTTAACCCTTTCTTATCTCATTTGGCCCAAATGTATAATGGCTGGGTTGGCAATATGAAAGTAAAGGTTCTATTGGCTGGTAATGCTTTTACGGCTGGTAAAATAATTATTAGTTGCATACCCCCTGGCTTTGCCGCACAAAATATTTCTATTGCCCAGGCCACCATGTTCCCCCATGTCATAGCTGATGTTAGGGTTTTGGAACCCATTGAGGTGCCATTGGAAGATGTGAGGAATGTGCTCTTCCACAACAATGACAACACGCCAACTATGCGGTTGGTGTGCATGCTATACACCCCCTTGCGGGCCAGCGGTAGCTCGTCTGGAACTGACCCCTTTGTGATTGCTGGGCGTGTTTTGACATGCCCAAGTCCTGATTTTAGCTTTTTGTTTTTGGTCCCCCCCAATGTAGAGCAAAAGACTAAACCTTTCAGTGTCCCAAACCTTCCATTGAATACCCTTTCAAATTCGAGAGTCCCTTCTCTAATCAAATCAATGATGGTGTCCAGGGACCATGGGCAGATGGTTCAGTTCCAAAATGGTAGGGTCACCCTAGATGGGCAGCTGCAAGGCACCACACCCACATCAGCTAGTCAGCTGTGCAAAATCAGGGGCAGTGTCTTCCATGCCAATGGTGGGGATGGGTATAACTTAACAGAATTGGATGGGAGCCCATACCACGCATTTGAGAGCCCCGCACCAATAGGGTTTCCAGATCTAGGTGAATGTGATTGGCACATGGAGGCTTCTCCTACCACCCAATTTAATACTGGAGAACTTATAAAACAAATCAATGTTAAACAAGAGTCAGCATTTGCCCCCCACCTTGGCACCGTACAGGTAGACGGCCTGGATGGTGTGAATGTCAACACCAACATGATAGCTAAATTAGGATGGGTGTCTCCTGTCACTGATGGACACAAAAAGGAAGTCGACCCGTGGGTCATTCCACGCTATGGTTCAACCCTGACTGAGGCCGCCCAATTAGCCCCCCCAATATATCCCCCAGGCTTTGGTGAGGCCATTGTGTTTTTCATGTCAGATTTTCCCATTACCCATGGCACCAATGGTTTGAGTGTGCCTTGCACTATACCCCAAGAATTTGTCACCCATTTTGTCAATGAACAGGCCCCTACTAGAGGGGAAGCAGCCCTACTACACTATCTGGACCCTGATACTAATAGAAATCTTGGTGAGTTTAAATTATATCCTGATGGCTTCATGACATGTGTGCCTAACTCCAGTGGCACTGGTCCACAAACTCTTCCAATCAATGGCGTCTTTGTCTTTGTGTCCTGGGTTTCTAGATTTTATCAATTAAAGCCTGTGGGAACAGCCGGCCCGGCTCGTAGGCTTG |
| MK073892.1 USA/2016/GI.3(P13) | ATGATGATCCATTTGAAACCTTGGTCCCACACACACAGAGGAAGGTCCAATTAATATCCCTATTAGGTGAGGCCGCACTCCATGGTGAGAAATTCTATAGAAAGATAGCCAGTAGGGTGATCCAGGAGGTTAAAGAAGGAGGATTGGAAATTTACATCCCTGGGTGGCAGGCCATGTTCCGCTGGATGCGATTCCATGATTTGAGTTTGTGGACAGGAGACCGCGATCTCCTGCCCGATTATGTAAATGATGATGGCGTCTAAGGACGCCCCAACAAACATGGATGGCACCAGTGGTTGGTACCAGAGGCAAATACGAGCCTATATCAATGGAGCCAGTGGCTGGGGCAGCGACAGCTGCCGCAACCGCTGGTCAAGTTAATATGATTGACCCCTGGATTATGAATAATTATGTGCAAGCCCCACAAGGTGAATTCACCATATCACCAAACAACACACCAGGTGATATTCTATTTGATTTACAATTAGGCCCTCACCTCAACCCCTTTTTATCCCATTTGGCTCAAATGTACAATGGATGGGTAGGTAACATGAAAGTGAAAGTTTTATTGGCAGGTAATGCTTTCACGGCAGGAAAAATTATTATTAGTTGTATACCCCCTGGTTTTGTCTCACAGAATATATCTATAGCTCAAGCTACAATGTTCCCCCACGTTATAGCTGATGTCAGGGTCCTGGAGCCCATTGAGGTCCCATTGGAGGATGTGAGGAATGTGTTGTTCCACAATAACGATAACACACCAACCATGAGACTGGTATGCATGCTCTACACCCCCTTGCGTGCCAGCGGAAGCTCATCTGGGACAGACCCCTTTGTGATTGCTGGGCGTGTGTTGACTTGCCCTAGTCCTGATTTTAGCTTCTTATTCTTAGTGCCCCCCAATGTAGAACAAAAGACTAAACCTTTTAGTGTCCCAGATCTCCCACTCAATGTTCTATCAAATTCAAGGGTGCCGTCACTGATAAAATTCATGATGGTGTCACAGGACCACGGACAGATGGTACAATTTCAGAATGGCAGGGTCACACTGGATGGACAGCTCCAGGGCACCACACCAACATCGGCCAGCCAGCTCTGCAAGATGCGGGGGACGGTTTACCATGCCTCCGGTGGTCAAGGGCTCAATTTAACTGAGATTGATGGCACCCCCTATCATGCATTTGAAAGCCCAGCTCCCATTGGTTTCCCTGACATAGGAGACAGCGATTGGCACATCAACGCTTCACCCGCCACCGCCTTCGGCAGTGGCGAAAGCGTCAAAAGACTAGATATGGAACAGGGCTCTTCATTTGCCCCACATTTGGGTACTGTACACTATACTAATGCCAGCTATGAAGCCAATGCTGATTTGATCTGCTCATTGGAGTGGCTATCCCCGCCTAGCGGAGGCACTCCAAACAAAGTCAATCCTTGGGCCATCCCACGGTATGGTTCAACTTTGACTGAAGCTGCTCAGCTGGCCCCACCAATTTACCCCCCTGGCTTTGGAGAAGCTATAGTTTTCTTTATGTCTGACTTTCCTATAGCAAATGGTCAAGATGGGTTAAGAGTGCCCTGCACTATACCCCAAGAATTTGTAACCCATTTTGTCAATGAACAAGCACCAACCCGTGGGGAAGCTGCCCTTCTTCATTATGTTGATCCCGACACACACCGCAATTTGGGTGAGTTTAAGTTATACCCTGAAGGATTTATGACTTGTGTACCAAATTCCAGTGGTAGTGGTCCCCAAACGCTGCCTATCAATGGAGTCTTCACTTTTGTCTCTTGGGTTTCTCGTTTCTATCAACTAAAGCCTGTGGGAACGACCGGACCGGTTCGTAGGCTAG |
| MH443711.1 CHN/2018/GI.5(P12) | ATGATGACCCAAATGAAACCCTGATACCCCACCCACAAAGAAAGGTTCAGCTTATATCTCTGCTTGGCGAGGCCTCACTACATGGGGAGAAGTTTTACAGGAAGATAGCTAGTAAGGTTATCCAGGAAATTAAAACTGGTGGTCTTGAAATGTACGTGCCAGGTTGGCAGGCAATGTTCCGCTGGATGCGCTTCCATGATCTTGGGTTGTGGACAGGAGATCGCAATCTTCTGCCTGAATTCGTAAATGATGATGGCGTCTAAGGACGCTACACCAAGCGCAGATGGCGCGAATGGCTCGTGCCGGAGGTTAATAAGAACCACTGCCTCTTGATCCTGTGGCTGGGGCTTCTACTGCCTTAGCTACTGCTGGTCAAGTTAATATGATTGATCCTTGGATTTTTAATAATTTTGTCCAAGCCCCTCAGGGCGAATTCACAATCTCCCCCAATAATACCCCCGGTGATATATTGTTTGATTTGCAATTGGGACCTCATCTCAATCCCTTCCTTGCCCATTTGTCACAAATGTATAATGGTTGGGTTGGTAACATGCGAGTCAGGGTCATCCTGGCAGGTAACGCATTTACCGCCGGTAAAGTGATAATTTGCTGTGTCCCCCCTGGCTTCCAATCTAGGACCCTATCAATTGCTCAGGCTACCCTCTTTCCTCATGTGATTGCTGATGTTCGTACATTAGAACCACTTGAAATCCCACTGGAGGATGTTAGAAACACACTGTACCATAATAATGACAGCCAGCCGACAATGCGCTTGTTGTGCATGCTGTACACGCCACTTCGCACTGGTGGTAGTTCTGGTGGCACAGACGCTTTTGTCGTCGCAGGGAGAGTTTTAACTTGCCCAAGTCCAGACTTCAATTTTCTCTTCCTGGTTCCCCCAACTGTGGAACAAAAGACACGGCCTTTTAGTGTCCCCAATATCCCACTCCAGAATCTTTCCAACTCCCGAGTTCCTAGCCTAATCCAAGGCATGGTTCTCTCTAATGACCATGCACAAACGGTCCAATTCCAGAATGGCAGGTGTACTACTGACGGTCACCTGCTGGGGACCACCCCTGTTTCTGCTGGTCAGCTTATGAAATTCAGAGGTAAGGTAACTTCAGGTTCAAAAGTACTAAATTTGACAGAACTAGATGGTTCACCATTTCTGGCCTTTGAGGCCCCAGCCCCTGCTGGATTCCCTGACCTTGGAAAGTGTGATTGGCACGTCGAGATGAGTCTCTATCAAGATAATAATCATGCAGACCCTATTGTGTTGCATGCCATTGAACCCAACTCATCTTCTTTTGTCCCGCACCTTGGTAGTGTTTCCTTTAAAGAGAATGTTAATGTAGCTGGTGATTATGTTTGTACCATACAATACACTTCACCCCCCTCTGATCCCCAAAATCCTAATGCTGATGTTGATTTCTGGTCTATCCCTGATTATGGTTCCAACTTGGCTGAGGCATCACAGCTCGCCCCAGTTGTTTACCCCCCTGGCTTCGGTGAGGCTATTGTCTACTTCATGTCGCGCATCCCCGGCTGGAATAGGACTAATAGGCTCAATCTAGTCCCTTGCCTGTTGCCCCAAGAGTTCATTGGACATTTCGTCAGTGAACAAGCCCCCGCAATTGGTGAGGCTGCCCTCTTACATTATGTTGACCCAGACACCAATCGGAACCTGGGTGAATTTAAATTGTACCCTGAGGGTTTTATTACTTGTGTCCCTAATGGCACAGGCCCTCAGCAGCTTCCCCTAAATGGTGTCTTTGTCTTTTCTTCCTGGGTGTCAAGATTTTATCAACTCAAGCCTGTGGGAACGGCCTCTTTGGCCAGAGGTAGGCTTG |
| MK236611.1 IRL/2016/GI.6(P11) | ACTCAGACCCCTTTGAAACCTTGGTGCCCCACCAGCAAAGAAAAATCCAGCTGATATCACTGTTAGGTGAGGCCTCATTGCATGGTGAAAAATTCTACAGGAAGATCTCAAGTAAAGTCATCCAAGAGATTAAGACAGGGGGTCTTGAAATGTATGTGCCAGGGTGGCAAGCCATGTTCCGCTGGATGCGGTTCCACGACCTTGGCCTGTGGACAGGAGATCGCAATCTCCTGCCCGAATTCGTAAATGATGATGGCGTCTAAGGACGCCCCAACATCCCCTGATGGCGCCAGTGGCTGGTACCGGAGGCTAATACGAGCAAATTTCAATGGACCCTGTTGCGGGTGCTTCAACAGCAGTCGCGACAGCTGGGCAAGTTAACATGATTGACCCATGGATTTTCAATAACTTCGTCCAGGCACCTCAAGGAGAATTCACTATTTCCCCTAATAATACCCCCGGTGATATTTTGTTTGATTTACAACTAGGACCCCACCTTAACCCATTTCTAGCCCATCTCTCACAGATGTACAATGGTTGGGTCGGCAATATGCGTGTGCGCATACTGTTGGCCGGGAATGCCTTCACAGCTGGAAAGATAATCATTTGCTGTGTCCCCCCTGGTTTTGATGCCAGAATACTCACAATAGCTCAAGCAACTCTCTTCCCACACTTAATTGCTGATGTAAGGACCCTTGAGCCTGTGGAGCTTCCTTTGGAGGATGTGCGCAATGTTCTCTTCCACAACAGTAGCCAACCGCAGCCAACAATGCGGTTGGTTGCTATGTTGTACACTCCCCTCCGCACTGGTGGTGGTTCCGGAGGCACTGATGCCTTTGTGGTCGCGGGTAGGGTGCTTACGTGCCCCGCCCCCGACTTTAGTTTTCTGTTTCTAGTCCCCCCTTCCGTTGAACAAAAGACCAGAGTTTTCAGTGTCCCCAACATACCCCTGAAGGATCTCTCAAATTCCCGTGTCCCTGTGCCAGTGCAGGGCATGTTTATGTCCCCAGATGTTAATCAGTCAGTCCAGTTTCAGAATGGACGCTGCCAAATTGATGGTCAACTCCAAGGCACCACTCCAGTCTCGCTCAGCCAACTCTGCAAGATTAGGGGTAAAACTTCTAGCAATGCTAGGGTGCTTAACTTAAGTGAGGTGGATGGCACACCCTTCATCCCACTTGAATCACCAGCGCCAGTTGGTTTTCCCGACTTGGGAGGTTGTGATTGGCATGTAAATTTCACTTTCCAGGCTCAAAATCAGGACCCATCTCAAAGTGTAACCTTTGCAACTAATGATGCTAGCTTTGTCCCCTATCTAGGTAGTATCTCTCCTCACAATGGGGGAGATTTTCATGCAGGTGACATCATAGGTAGCCTTGGTTGGATTTCAGCCCCGTCTGATAATTCACAACTTAATGTTTGGACAATACCAAAGTATGGATCTAGTCTCCCAGATGTCACTCATCTTGCCCCTGCTGTGTTCCCCCCAGGCTTTGGGGAGGTGATCTTGTACTTCTATTCTACCTTCCCAGGTTCTGGACAATCCAGCCAACTTCAAGTCCCATGCTTGTTGCCTCAGGAGTTTATCACCCACTTCTGTAACGAACAGGCTCCCATCGCTGGGGAGGCTGCCCTCCTCCACTACGTGGACCCTGATACGGGGCGAAATTTGGGAGAATTCAAACTTTACCCTGATGGGTTTATGACCTGTGTCCCCAATAGTGTTAGTAGTGGCCCTCAAACCCTTCCTATCAATGGAGTCTTTGTCTTTGTTTCATGGGTATCTAGATTCTATCAACTCAAGCCTGTGGGAACGGCCTCAGCGGCTAGAAGGCTTG |
| NC 044854.1 JPN/2000/GI.6(PNA1) | ACTCAGACCCTTTTGAAACCTTAGTGCCCCACCAGCAAAGAAAAGTCCAATTGATATCACTGCTGGGTGAGGCCTCATTGCATGGTGAAAAGTTCTACAGAAAAATTTCTAGCAAAGTCATCCAAGAGATCAAGACAGGGGGCCTTGAAATGTACGTACCAGGGTGGCAAGCCATGTTCCGCTGGATGCGGTTCCATGATCTTGGTCTGTGGACAGGAGATCGCAATCTCCTACCCGAATTTGTAAATGATGATGGCGTCTAAGGACGCCCCAACATCCCCTGATGGCGCCAGTGGCTGGTACCGGAGGCTAATACGAGCAAATTTCAATGGACCCTGTTGCGGGTGCTTCAACAGCAGTCGCAACAGCTGGGCAAGTTAATATGATTGACCCATGGATTTTCAATAACTTCGTCCAGGCACCCCAAGGAGAATTTACTATTTCCCCTAATAATACCCCCGGTGACATTTTGTTTGATTTACAATTAGGACCCCACCTTAATCCATTTCTAGCCCACCTCTCACAAATGTATAATGGTTGGGTCGGCAATATGCGTGTACGTATATTGTTGGCAGGGAATGCTTTTACAGCTGGTAAGATAATCATTTGCTGTGTCCCCCCTGGCTTTGATGCTAGAATACTTACAATAGCCCAAGCAACTCTCTTTCCACACTTGATTGCTGATGTTAGGACTCTTGAGCCTGTGGAGCTCCCTCTGGAGGACGTGCGCAACGTTCTCTATCACAACAGCAGCCAGCCGCAGCCAACAATGCGGTTGGTAGCTATGTTGTACACTCCCCTCCGCACTGGTGGTGGCTCCGGAGGTACTGATGCCTTTGTGGTTGCGGGCAGGGTGCTCACGTGCCCCGCCCCTGATTTCAGCTTTCTGTTTCTCGTCCCCCCCTCCGTTGAGCAGAAGACTAGGGTTTTTAGTGTCCCCAACATACCCCTGAAAGACCTTTCAAATTCTCGTGTCCCTGTGCCTATACAGGGCATGTTCATGTCCCCAGATGTCAATCAGTCAGTCCAGTTCCAGAACGGGCGCTGCCAGATCGATGGTCAACTCCAGGGCACTACCCCTGTCTCGCTCAGTCAACTTTGCAAGATTAGGGGTAAGACTTCAAGCAATGCCAGGGTACTCAACTTAAGTGAGGTGGATGGCACACCCTTTATCCCTCTTGAGTCTCCAGCACCAGTTGGTTTTCCCGACTTAGGAGGTTGTGACTGGCATGTAAACTTCACTTTCCAGGCTCAGAATCAGGACCCATCTCAAAGTGTGACCTTTGCAACTAATGATGCTAGCTTTGTTCCCTACTTAGGCAGCATTTCTCCTCACAATGGGGGGGATTTTCATGCAGGTGACATCATAGGCAGCCTTGGCTGGATTTCAGCCCCGTCTGACAATACACAACTTAATGTCTGGACAATACCAAAGTATGGGTCTAGTCTCCCAGATGTCACTCACCTTGCACCTGCTGTGTTCCCCCCAGGCTTTGGGGAGGTGATTCTGTACTTCTACTCTACATTCCCAGGTTCTGGACAACCTAGTCAGCTTCAAGTGCCATGCCTGTTGCCTCAGGAGTTCATCACCCACTTCTGCAATGAACAGGCTCCCATCGCTGGGGAAGCTGCTCTCCTCCATTACGTGGACCCTGACACGGGACGGAACCTGGGGGAATTCAAACTCTATCCTGATGGGTTCATGACCTGTGTCCCCAATAGTGTTAGTAGTGGCCCTCAAACCCTTCCTATCAATGGAGTCTTTGTCTTTGTTTCATGGGTATCCAGATTTTATCAACTCAAGCCTGTGGGAACGGCCTCAGCGGCTAGAAGGCTTG |
| KX907730.1 USA/2014/GI.7(P7) | ATGATGACCCCTTCGAAACTCTGATACCCCATCAACAAAGGAAAATTCAATTGATATCCCTGCTTGGTGAGGCTGCACTCCATGGAGAGAAATTTTACAGGAAGATTGCTAATAGGGTGATACAAGAAGTTAAGGAGGGAGGGCTTGAACTCTACATACCAGGTTGGCAGGCCATGTTTCGCTGGATGCGATTCCATGACTTGAGCATGTGGACAGGAGATCGCAATCTACTGCCCGATTATGTAAATGATGATGGCGTCTAAGGACGCCCCCTCAAACATGGATGGCACCAGTGGTTGGTTCCAGAGGTTAATGCGAACCTCTACCCCTTGAACCGGTGGTGGGCGCCGCAACTGCGGTGGCCACCGCTGGACAGGTTAATATGATAGACCCCTGGATTATGAATAATTTTGTCCAAGCCCCTGAAGGCGAATTTACTATTTCACCCAACAACACCCCTGGAGATATTTTATTTGATTTGCGTCTGGGACCACACCTTAACCCCTTTCTCCTGCATTTGTCCCAGATGTATAATGGATGGGTTGGGAATATGAGAGTTAGGGTCATGCTTGCAGGGAACGCGTTCTCTGCTGGTAAGATCATAATTTGTTGTGTCCCACCTGGCTTTGAATCCCAGAACATATCAATTGGACAAGCCACAATGTTTCCCCATGTCATTGCTGATGTGAGAGTCCTGGAGCCCATAGAGGTCCCCCTTGATGATGTGCGCAATGTCCTGTTCCACACAAATGAGAACCGCCCAACCATGCGACTGCTCTGTATGTTGTACACACCTCTCCGTGCTGGAGGAGCTTCATCAGGCACAGACCCTTTTGTCATTGCTGGCCGTGTGTTAACTTGCCCTGCTCCTGATTTCAATTTTCTGTTTCTTGTGCCCCCTAGTGTAGAACAAAAGACCAGGCAATTGACTATACCCAATATACCCTTGAACAATTTGGCTAACTCCAGAGTGCCTGCAATGATAAACAAGATGACAGTCTCTGCTGATCAGAATCAGGTGGTCCAGTTTCAAAATGGCCGTTGCACACTTGAAGGGCAACTCTTAGGCACAACCCCTGTTTCAGCCAATCAAGTTGCCCGTATACGTGGCAAAGTCTTTTCCACCAACTCCGGGACGGGGCTTAATTTAACTGAAGTAGATGGAACCCCCTACCATGCATTTGAGAGCCCGGCCCCTCTCGGTTTTCCTGACATTGGTAATTGTGATTGGCACGTGTATGCCTTTAAGGTAAATCAAAACACAGGGGACCCCATGTACAGGCTGGACATAACACAAGGAAACTCATTTGCGCCGCATCTTGGCTCCATTGAGTTTAGTTCTGAAAACCACCCATCGGGGGACCAACTAGGCACACTTACTTGGATCTCCCCTCTTAACAATGCTTCTAGGGTTGATCCCTGGAAAATCCCAACATATGGGTCGACATTGACTGAGTCCACCAACTTGGCACCACCCATCTTTCCACCAGGTTTTGGTGAGGCTATAGTTTATTTTATGTCAGATTTTCCTATTGTGTCAGGTAATACTGCCCAAATCCCCTGCACCCTGCCCCAAGAATTTGTTTCATCCTTTGTGGAGCAGCAGGCACCGATCCGAGGTGAAGCTGCTCTATTGCATTATGTGGACCCAGATACCCATCGCAATCTGGGAGAATTCAAATTGTACCCTGATGGGTTCATCACTTGTGTGCCTAACACGGGTGGCGGCCCCCAAAATCTCCCATCGAATGGTGTCTTTGTTTTCTCTTCTTGGGTTTCTAGATATTATCAATTAAAGCCTGTGGGAACAACCGGTCCGGTTAGGAGGCTTG |
| MH130046.1 BGD/2011/GI.7(PNA2) | ATGATGATCCATTCGAAACTCTGGTTCCACATTCGCAGAGAAAAGTCCAGTTGGTGTCTTTGTTAGGTGAAGCTGCTCTCCATGGTGAAAAGTTTTATAGGAAAATAGCCAGCAGAGTTATTCAAGAAGTCAAGGAAGGGGGGCTTGAGATCTACATACCCGGGTGGCAGGCCATGTTCCGCTGGATGCGGTTCCATGATTTGAGTTTGTGGACAGGAGATCGCGATCTCTTGCCCGATTATGTAAATGATGATGGCGTCTAAGGACGCCCCTTCAAACATGGACGGCACTAGTGGTTGGTACCAGAGGCAAATACGATCCTATTCCTTTAGAACCTGTAGTGGGGGCTGCAACTGCAGCCGCTACGGCAGGTCAAGTTAATATGATTGACCCCTGGATTATGAATAATTTTGTACAGTCACCAGAGGGCGAGTTCACAATTTCCCCAAACAACACGCCTGGTGACATTTTATTTGATTTACAATTGGGCCCCCATTTGAATCCATTCCTGCAGCATCTATCACAAATGTACAATGGCTGGGTTGGCAACATGAGGGTTAGAATTTTGCTAGCAGGGAATGCGTTTTCTGCTGGCAAGATAATAATCTGTTGTGTGCCACCTGGCTTTACCGCCCAGAATATCTCTATTGCTCAAGCCACAATGTTCCCACATGTCATTGCTGATGTTCGAGTTTTAGAGCCCATTGAGATTCCCCTTGATGATGTTCGGAATGTGCTCTTCCACACAAATGAGAATAGACCCACAATGCGCTTATTGTGCATGCTCTATACCCCATTGCGGGCCGGGGGCGCGTCTGCGGGCACGGATCCGTTCGTGATTGCAGGGCGCGTCTTAACGTGCCCTTCTCCAGACTTTAATTTCTTGTTTCTAGTCCCCCCCAGTGTTGAACAGAAAACTAGGCAATTAACTGTCCCCAACATCCCATTAAACAATCTATCAAATTCTAGAGTGCCCGCCATGATCAATAAGATGGCTATTTCCCCAGATGCTAATCAGGTTGTCCAATTTCAAAATGGCAGGTGCACTACAGATGGTCAGCTGTTGGGCACAACCCCAATCTCTGCGAGTCAAGTGGCCCGCATCCGCGGCAAAGTCTTTTCTACTTCATCAGGCAAAGGATTGAATCTGTCTGAGCTGGATGGCAGTCCATACCATGCCTTTGAAAGCCCTGCCCCGGTTGGTTTCCCTGATCTTGGGCATTGTGATTGGCATGTCTCTTCCTTCAAGGCAGATCAAAACTCCAGAGATGATCCCATTTCACGCCTAGATATAAAACAAGGGGCTTCCTTTGCCCCACACCTGGGTTCTATTGAATACACCACTTCGCAAAACCCAGATGGTGATCAGTTAGGCACCCTAACCTGGATCTCATCCCCATCAGAAGATACTCCTGGTCATGGTACAATCAACCTATGGAAGATTCCTTCCTATGGTTCCACAGTCACTGAGTCAGTCCATCTTGCACCCCCAATATACCCTCCAGGGTTTGGGGAGACACTTGTTTATTTTATGTCTAACTTTCCAATTGGGCACACTAGTTCCAGTATGGCACAAGTCCCATGCACCCTCCCTCAGGAATTTGTGGCACACTTTGTTAATGAACAGGCCCCTGTTCGAGGGGATGCAGCATTGCTTCACTATGTGGACCCTGACACACACAGAAATCTTGGTGAGTTCAAATTATACCCTGAAGGTTACATAACCTGTGTGCCAAACACGGGTGGTGGTGGCCCCCAAAGTCTCCCAACCAATGGAGTATTTATCTTCTCATCTTGGGTATCCAGATATTATCAACTTAAGCCTGTGGGAACGGCTGGACCAGCCAGGAGGCTTG |
| KP407450.1 CHN/2008/GI.8(P8)/Huzhou | ATGAAGACCCGTTTGAGACTCTGGTGCCACACTCACAAAGAAAAGTTCAATTGATATCCCTACTGGGTGAGGCCGCCCTACATGGTGAAAAATTCTACAGGAAGATAGCCAGTAGGGTTATCCAAGAAGTTAAGGAAGGGGGTCTGGAGATCTATATCCCTGGGTGGCAGGCCATGTTCCGCTGGATGCGATTCCATGACTTAAGTTTGTGGACAGGAGATCGCGATCTCTTGCCCGATTATGTAAATGATGATGGCGTCTAAGGACGCCCCAACAAACATGGATGGCACCAGTGGTTGGTACCAGAGGCAAATACGAACCTTTACCAATAAAACCAGTGGCCGGGGCTGCGACTGCGGTTGCAACCGCCGGTCAGGTAAATATGATTGACCCCTGGATAATGAACAATTTTGTACAGGCTCCCCAAGGGGAGTTTACCATTTCCCCAAATAATACACCTGGTGATATTTTATTTGATTTGCAATTAGGACCACATCTTAATCCTTTCTTGGCTCATTTGTCTAGAATGTATAATGGTTGGGTTGGGAATATGCAGGTCCGCATAATGCTTGCTGGTAATGCCTTTTCAGCTGGAAAGATTATTGTGTGCTGCATACCGCCGGGTTTTTCTTCTCAATCTATTTCTATTGCTCAAGCTACAATGTTCCCACATGTCATAGCTGATGTCAGAGTGTTAGAACCTATTGATGTACCCTTAGATGATGTTAGAAATGTCCTTTTTCATAATAATGATAACCCCCAAACGATGAGATTGCTTTGCATGTTGTATACACCCCTTCGGTCTGGTGGTACATCTTCAGGTACAGATCCTTTTGTCATTGCTGGTCGTGTCTTGACCTGCCCCACACCTGATTTTAGTTTCTTGTTCCTTGTCCCTCCTGATATCGAGCAAAGAACTAAGCCCTTCAGTGTACCCAATATCCCTATGAACCTGATGTCCAATTCCCGTGTTTCAATGTTGATTGATGGTATGATGGTTTCAAATGATCAGAATCAAGTCCCCCAGTTTCAGAATGGTCGAGTCACTTTAGATGGACAGCTGCAGGGTACCACCACAGTTTCAGCTGCGTGTGTAGCAAGGATGAGGGGGCGCATTTTTAATAATAATGGCAATTATGGTGTGAATCTGACTGAACTGGATGGCAATCCCTATCATGCCTTTGATAGCCCGGCTCCTTTAGGCTTTCCTGACTTTGGGAATTGTGATTTGCACATGACCTTTGTTAAAATTAATCCCAATGAGTTGTCCTCTGGAGACCCTTCTGGCAAAGTGGTCATTCGTTCCTACGATGCCACTTTTGCCCCCCATCTTGGTACTGTCAAACTGGAGAATGATGATGAGCTAGCTCGGTTTGTGGGTAAGGAGGTAGTACTGGAGCTGACGTGGGTTTCCAACAGAGAGGGTGCCACCCTTAATCTATGGGCTGTTCCAAATTATGGTTCCAGTTTGACACAGGCGTCACAGCTTGCCCCACCAATTTACCCCCCTGGGTTTGGTGAGGCTATTGTGTATTTCACCTCTACTTTTCCAACAGTCAGCAACCCAAAAGTCCCATGTACACTTCCTCAAGAATTTGTTTCACATTTTGTCAATGAACAGGCCCCCACACGCGGCGATGCCGCTCTTCTTCATTATGTTGATCCTGACACACACCGTAATCTTGGAGAGTTTAAGATGTACCCTGAGGGCTACATGACATGTGTCCCTAATGCTGGTGGTGGGCCACAAACCTTACCAATCAATGGTGTATTTGTCTTCATTTCTTGGGTTTCTAGATATTACCAGTTAAAGCCTGTGGGAACTGCCGGTGCGGCTAGGAGGCTTG |
| KX907731.1 USA/2016/GI.9(P9) | ATGATGACCCATTTGAAACTTTGGTGCCTCATCAGCAAAGAAAGATACAACTGATTTCCTTGCTTGGGGAGGCTGCACTTCACGGAGAAAAATTCTACAGAAAGATTGCCAACAGAGTGATTCAGGAAGTCAAAGAGGGGGGCCTTGAGCTTTATGTGCCCGGCTGGCAGCCCATGTTCCGCTGGATGCGGTTCCATGATCTGAGCTTGTGGGCAGGAGATCGCAATCTCCTCCCCGATTATGTAAATGATGATGGCGTCTAAGGACGCTACCTCAAACATGGATGGCACCAGCGGATGGTGCCAGAGAATAATAACGAACCAATCAACATGGAACCAGTCGCGGGCGCGGTCACTGCCGCAGCTACGGCTGGCCAGGTGAATATGATTGATCCTTGGATAATGAATAATTATGTACAAGCCCCCCAGGGCGAGTTCACAATTTCGCCCAATAATACTCCTGGTGATATATTGTTTGATCTTCAATTAGGTCCCCATCTAAACCCCTTTCTTGCCCATTTATCCCAAATGTATAATGGATGGGTTGGTAACATGAAAGTCAGGGTGGTGTTGGCTGGCAATGCCTTTTCTGCGGGAAAGATCATAGTGTGCTGTATACCACCTGGATTCTCTGCACCAAACATATCCATAGCCCAGGCAACTATGTTCCCTCATGTTATTGCTGATGTTCGGGTCTTGGAGCCCATTGATATTCCCCTTGATGATGTCAGGAATGTTCTGTTTCATAATAATGATAATGGTAATCAGACTATGAGATTGCTGTGCATGTTGTATACACCACTCCGTAGTGGAGGCACGTCCAGTGGAACTGACCCGTTTGTGATTGCTGGTAGGGTTCTAACCTGCCCTACTCCAGATTTTAATTTCTTGTTCTTGGTTCCCCCAACCGTTGAGCAAAAGACCAAACAGTTTAGTGTGCCTAACCTTCCCCTCAATGTTATGTCTAATTCTAGAGTTCCATCACTCCTAAATGCTATGGTGGTGTCACCAGATCAGGCCCAAGTGGTGCAATTTCAAAATGGCAGGTGCACCCTTGATGGGCAAATGTTGGGCACCACCACTGTATCAGCTAGCTGTGTTGCAAGATTCAGAGGGAAAACTTTTCAAGCCCCTGACAACAGGTTAGGTATCAATCTGGCTGAAATTAGTGGAGAACCATACCATGCATTTGAAAGCCCTGCCCCCCTAGGTTTCCCTGACTTTGGGGATGGAGATTGGCACGTCACTGCAACAAAAGTGACCCCCTCACAGCTTGAGGCTAATGACCCTGTGGTCATGGGGAATGTCCAACCATATAACCCACAGTTTGCTCCTCACCTGGGCACCCTAGTCGTAGAAAACCCTACTCCAGACAATGTGACCACCGGGACTGACTTATTGTTCAACATAACCTGGCTTTCCAATCGTGCCAATAATCGGTTTAACCCTTGGGTTATCCCAAATTATGGTTCAACTCTTACTGAGGCAGCACAGCTGGCACCTTCAATATTTCCCCCTGGTTTTGGTGAAACTATAGTGTATTTCAATTCCACTTTTCCAGCTGTGGGGGCCACCACCCATGCAGCTATACCCTGTCTGCTTCCACAAGAATTTGTTGCCCATTTTGTTAATGAACAGGCCCCCATACGTGGAGAGGCTGCTTTGCTCCACTACATTGACCCTGATACTCACCGTAATCTTGGTGAGTTCAAAATTTATCCAGAGGGCTTTGTAACGTGTGTTCCTAATGTGGGTGGTACGGGGCCACAATCTCTACCTACTAATGGTATCTTTGTCTTTGTCTCTTGGGTGTCAAGGTACTATCAACTCAAGCCTGTGGGAACTGCTGGTCAAGCAAGGCGGCTTG |
